# A framework for counterfactual analysis, strategy evaluation, and control of epidemics using reproduction number estimates

**DOI:** 10.1371/journal.pcbi.1012569

**Published:** 2024-11-20

**Authors:** Baike She, Rebecca Lee Smith, Ian Pytlarz, Shreyas Sundaram, Philip E. Paré

**Affiliations:** 1 School of Electrical and Computer Engineering, Georgia Institute of Technology, Atlanta, Georgia, United States of America; 2 Department of Pathobiology, University of Illinois Urbana-Champaign, Champaign, Illinois, United States of America; 3 Institutional Data Analytics + Assessment, Purdue University, West Lafayette, Indiana, United States of America; 4 Elmore Family School of Electrical and Computer Engineering, Purdue University, West Lafayette, Indiana, United States of America; Stockholms Universitet, SWEDEN

## Abstract

During pandemics, countries, regions, and communities develop various epidemic models to evaluate spread and guide mitigation policies. However, model uncertainties caused by complex transmission behaviors, contact-tracing networks, time-varying parameters, human factors, and limited data present significant challenges to model-based approaches. To address these issues, we propose a novel framework that centers around reproduction number estimates to perform counterfactual analysis, strategy evaluation, and feedback control of epidemics. The framework 1) introduces a mechanism to quantify the impact of the testing-for-isolation intervention strategy on the basic reproduction number. Building on this mechanism, the framework 2) proposes a method to reverse engineer the effective reproduction number under different strengths of the intervention strategy. In addition, based on the method that quantifies the impact of the testing-for-isolation strategy on the basic reproduction number, the framework 3) proposes a closed-loop control algorithm that uses the effective reproduction number both as feedback to indicate the severity of the spread and as the control goal to guide adjustments in the intensity of the intervention. We illustrate the framework, along with its three core methods, by addressing three key questions and validating its effectiveness using data collected during the COVID-19 pandemic at the University of Illinois Urbana-Champaign (UIUC) and Purdue University: 1) How severe would an outbreak have been without the implemented intervention strategies? 2) What impact would varying the intervention strength have had on an outbreak? 3) How can we adjust the intervention intensity based on the current state of an outbreak?

## Author summary

We propose a framework that centers around reproduction number estimates for counterfactual analysis, strategy evaluation, and feedback control of epidemics. The framework introduces a mechanism to quantify the impact of the testing-for-isolation intervention on the basic reproduction number first. It offers a method to reverse engineer the effective reproduction number under alternative strengths of this strategy, enabling the reconstruction of hypothetical spreading scenarios under different strength of intervention. Additionally, quantifying the impact of the testing-for-isolation intervention on the basic reproduction number facilitates the development of a closed-loop feedback control algorithm. This algorithm uses the effective reproduction number both as feedback to assess the severity of the spread and as the control goal to adjust the strength of the intervention. We leverage this framework to: 1) Analyze the effectiveness of the implemented intervention strategy by examining the connection between the testing-for-isolation intervention and the basic reproduction number; 2) Evaluate the impact of varying intervention intensities using a reverse engineering method to generate effective reproduction numbers of hypothetical spreading scenarios under different intervention strengths; and 3) Design a closed-loop feedback epidemic control framework for intervention intensity adaptation through the closed-loop control algorithm. This framework provides new insights into utilizing the reproduction number estimates to analyze hypothetical spreading scenarios under alternative levels of intervention strategies. Additionally, it uses the effective reproduction number as feedback information to design the closed-loop control strategy for dynamically adjusting the strength of the intervention to support epidemic mitigation efforts. Using COVID-19 data from UIUC and Purdue, we illustrate and validate our framework by conducting counterfactual analyses to evaluate the effectiveness of the implemented testing-for-isolation strategies. Additionally, we validate that our feedback control algorithm can effectively adjust the intervention strategy based on the severity of the epidemic.

## 1 Introduction

Since 2019, the COVID-19 pandemic caused by SARS-CoV-2 has significantly affected societal work patterns [1, 2]. Proactive epidemic intervention policies were essential to prevent outbreaks [3, 4]. To assist in evaluating the effectiveness of implemented pandemic intervention strategies and in designing feasible epidemic mitigation policies, spreading models have played an important role in policy-making [5, 6]. However, model uncertainties, introduced by complex transmission behaviors [7], contact-tracing networks [8], time-varying spreading parameters [9], human factors [10], and insufficient data [11], have posed significant challenges for model-based approaches [12]. Meanwhile, model-free approaches such as deep learning and reinforcement learning frameworks require extensive data and real-world trials in an actual epidemic spreading environment [13]. Further, due to the irreversible nature of epidemic spread, it is impossible to replicate the exact same process to test different intervention strategies or verify optimal resource allocation through control system design. Since the reproduction number effectively indicates the severity of disease spread by encapsulating key model information and can be estimated with relatively limited data, we present a framework that focuses on using reproduction number estimates to analyze, evaluate, and design pandemic intervention strategies.

The reproduction number captures the average number of new infected cases generated by a single infected individual [14–17]. Leveraging the reproduction number to evaluate the epidemic spread and assist in policy-making is widely accepted [18–20]. Nowadays, researchers and policy-makers mainly leverage the reproduction number to analyze and predict epidemic spreading processes. Our framework first introduces a mechanism that quantifies the impact of the level of the intervention strategy on the basic reproduction number. This mechanism forms the foundation for two additional methods we propose. The first is to reverse engineer the effective reproduction number under the intervention strategy for a real-world spread to the effective reproduction number under an alternative strength of the intervention strategy for the corresponding hypothetical spread. This method facilitates the analysis of implementing different strengths of the intervention strategy on the same spread, which is irreversible in the real world. The second method, based on the same mechanism, is a closed-loop feedback control algorithm. We use the effective reproduction number as feedback information and the strength of the intervention strategy as the control variable to influence the effective reproduction number and, consequently, the disease spread. This approach is well-suited for managing uncertainties, as feedback control systems are designed to handle such challenges.

The paper is organized as follows. In general, we introduce and validate the framework centered around reproduction number estimates, along with three methods, using COVID-19 spread data and the testing-for-isolation intervention strategies implemented at the University of Illinois Urbana-Champaign (UIUC) and Purdue University. An overview of the framework is provided in Section 2.1, followed by a description of the spread environment and data used for validation in Section 2.2. Section 3 demonstrates how the framework facilitates key analyses, including the quantification of the intervention strategy’s impact on the basic reproduction number (Section 3.1 and Section 5.1), counterfactual scenarios assessing what might have happened without the implemented strategies on both campuses (Section 3.4), and the effects of varying the strength of the testing-for-isolation strategy on the spread (Section 3.5). Additionally, in Section 3.6, we explore whether a closed-loop feedback control algorithm that adjusts the intensity of the strategy based on the effective reproduction number’s indication of the spread’s severity could outperform a fixed open-loop control intervention strategy. We explain the methods in detail, including how to quantify the impact of the testing-for-isolation strategy on the basic reproduction number in Section 5.1, reverse engineer the effective reproduction number for counterfactual analysis in Section 5.3, and use the effective reproduction number as both an observation and a control variable in a closed-loop control design in Section 5.4. Limitations and future directions are discussed in Section 4. Comprehensive analyses are provided in Section 2 in S1 Appendix.

## 2 Background

### 2.1 A framework for counterfactual analysis, strategy evaluation, and control of epidemics

**Fig 1:**
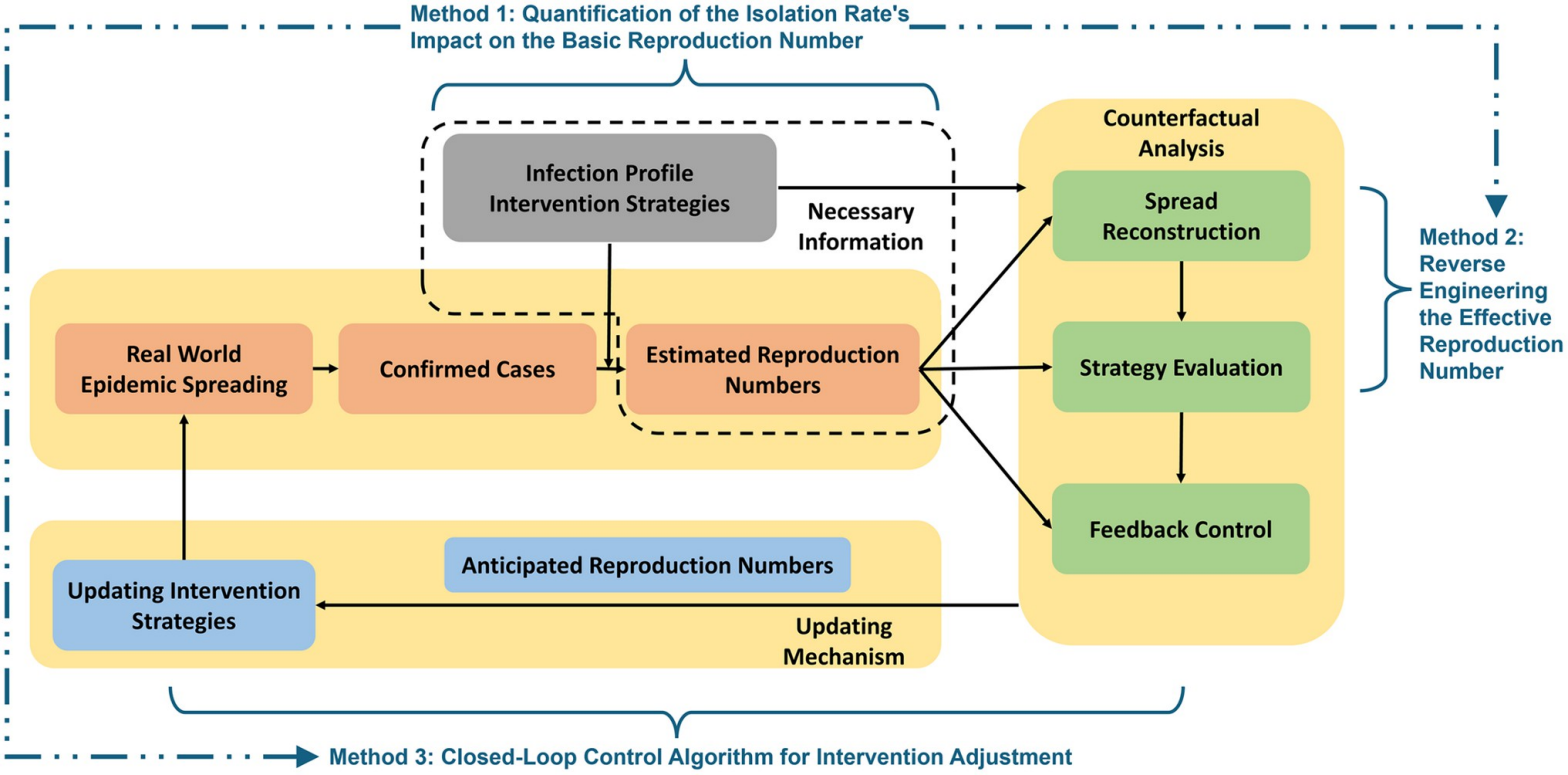
A framework for counterfactual analysis, strategy evaluation, and feedback control of epidemics using reproduction number estimates. The framework consists of three pieces and three methods. The first piece is to leverage real-world spreading data to estimate the effective reproduction number, where we propose a method to quantify the impact of the isolation rate on the basic reproduction number. The second step involves performing counterfactual analysis by introducing a method to reverse engineer the effective reproduction number, enabling simulation of hypothetical spreading scenarios without the implemented intervention strategy or with an alternative strength of intervention. The third component introduces a closed-loop control algorithm that uses the effective reproduction number as feedback to adjust the isolation rate, which in turn influences the effective reproduction number to manage the spread.

The proposed framework is shown in Fig 1. By leveraging data from a real-world epidemic, the first step is to estimate the effective reproduction number. This estimation process relies on 1) the initial infection profile and 2) the quantification of the intervention strategy’s impact on the spread. We explain these concepts in detail later in the paper, when we introduce the first method to quantify the effect of the testing-for-isolation strategy on the basic reproduction number. The methodologies for epidemic reconstruction, intervention strategy evaluation, and feedback control all depend on estimating the effective reproduction number and quantifying the impact of the intervention on the basic reproduction number. Using the estimated effective reproduction number, the framework first reconstructs the spreading process under the same implemented intervention strategies. It then performs a counterfactual analysis, reconstructing hypothetical scenarios to evaluate what would have happened if the intervention strategy had not been implemented or if an alternative strength of the strategy had been applied. We assume that nothing else changes in the hypothetical spreading scenario, meaning that the change in the strength of the intervention strategy does not further impact students’ behavior, viral loads, or other factors. Both analyses use the method of reverse engineering the effective reproduction number. Additionally, quantifying the intervention strategy’s impact on the basic reproduction number forms the foundation of the closed-loop feedback control algorithm, which adjusts the strength of the intervention strategy according to the severity of the spread. The effective reproduction number serves as feedback to gauge the pandemic’s severity, guiding adjustments to the intensity of the intervention strategy.

We consider the intervention strategies in Fig 1 as the testing-for-isolation strategy that removes infected individuals from the population. Inspired by the successful implementation of testing-for-isolation strategies during the COVID-19 pandemic [21], particularly at the University of Illinois Urbana-Champaign and Purdue University, we leverage the data collected by both universities to validate our framework.

### 2.2 Testing-for-isolation strategies at UIUC and Purdue

We leverage the aggregated COVID-19 data collected by UIUC and Purdue to validate the framework and the proposed methods. Plenty of universities, including Cornell University, Emory University, Purdue University, and the University of Illinois Urbana-Champaign, implemented the testing-for-isolation strategy during the COVID-19 pandemic [22–30]. This strategy actively tests a proportion of the university’s population and isolates those who test positive to prevent the infected population from spreading the virus. We collected and studied data from both universities to investigate their testing-for-isolation strategies during the COVID-19 pandemic through close collaboration with the Institutional Data Analytics + Assessment team (IDA+A) at Purdue University and the SHIELD: Target, Test, Tell team (SHIELD) at the University of Illinois Urbana-Champaign. Both teams adopted testing-for-isolation strategies to assess the severity of the pandemic and made necessary adjustments to their plans. These strategies successfully maintained safe operations on the campuses during the pandemic. More discussion on unique methodologies implemented by different universities is in Section 1A in S1 Appendix.

In order to introduce and validate our methods, we specifically focus on an early stage of the pandemic, between Fall 2020 and Spring 2021, when pharmaceutical interventions were not yet available. We consider the policy implemented by UIUC, which tested the entire campus twice a week during Fall 2020. We also consider Purdue University’s policy which tested a proportion of the entire campus weekly. We define *testing* as the process of sampling and confirming whether an individual is infectious. We define *isolation* as the process of removing infectious individuals from the population so that they cannot spread the virus. The testing rate should be greater than or equal to the isolation rate because we can isolate only those who test positive, whereas not everyone who tests positive is ensured to be isolated. For simplicity, we assume uniformly random sampling at both universities. Additionally, since we use daily aggregated data, we do not consider contact-tracing-based analysis that may leverage spatial data or networks. We make simplifying assumptions about the strategies implemented by UIUC and Purdue when validating our proposed framework and methods. The approaches implemented by both universities were considerably more intricate. More detailed information about the testing data, methods, and resources implemented by UIUC and Purdue can be found in Sections 1B and 1C in S1 Appendix.

## 3 Results

In this section, we introduce and validate the proposed framework and methods shown in Fig 1 by studying the epidemic spread on the UIUC and Purdue campuses. We first present the impact of the testing-for-isolation strategy on the infection profile and the basic reproduction number in Section 3.1. Then, we leverage the COVID-19 data from UIUC and Purdue to estimate their effective reproduction numbers in Section 3.2. We show that the estimated effective reproduction numbers can infer the spreading processes at both universities. We leverage the estimated effective reproduction number to reconstruct the spreading processes on the UIUC and Purdue campuses in Section 3.3. By leveraging the connection between the basic and effective reproduction numbers, we extend the mechanism that quantifies the relationship between the testing-for-isolation strategy and the basic reproduction number to also quantify its relationship with the effective reproduction number (see Section 5.3). Further, we leverage this relationship to reverse engineer the effective reproduction number to perform counterfactual analysis on what would have happened if different intensities of the isolation rate had been employed in Sections 3.4 and 3.5. Finally, in Section 3.6, we compare the performance of 1) our proposed closed-loop feedback control algorithm, which can adjust the intensity of the isolation rate based on the severity of the pandemic (i.e., the effective reproduction number), with 2) the fixed isolation rate on the UIUC campus, in the hypothetical spreading scenario obtained from the counterfactual analysis. More details on the methods we propose for analysis can be found in Section 5 and in Section 2 in S1 Appendix.

### 3.1 The impact of the isolation rate on the basic reproduction number

We introduce the method to quantify the isolation rate’s impact on the basic reproduction number in this section. One standard way of characterizing epidemic spread is to use contact tracing data to create an *infection profile*, also known as the generation time interval [31, 32]. The infection profile represents the average time between the onset of symptoms of a primary case and its secondary cases [16, 33]. It is also used to estimate critical epidemiological parameters such as the reproduction number, generation time, and attack rate [31, 32, 34–37]. In epidemiology and infectious disease modeling, the infection profile (or infectivity profile) refers to how infectiousness changes over the course of an individual’s infection. It can be considered as a function of calendar time since infection. Typically, the profile reflects pathogen shedding, with a single peak indicating pathogen growth followed by immune suppression or host death. This profile also indicates the effective contact rate between infectious and susceptible individuals, which can vary [16, 38]. For example, it may increase if a person coughs or sneezes due to respiratory disease, or decrease if they take to bed with illness [34]. Additionally, the profile can change during an epidemic as public health measures, such as the testing-for-isolation strategy in this work, are implemented [39].

Depending on our focus, we can define the infection profile as either a continuous or discrete function. The basic reproduction number is defined as the average number of new cases of an infection caused by one typical infected individual in a population consisting solely of susceptible individuals [15]. Then, the basic reproduction number can be obtained by accumulating the average number of infected cases generated by one infected individual over calendar time since infection, i.e., the infection profile. Building upon the idea that intervention strategies can affect the infection profile, and the infection profile will determine the reproduction number, we first propose a method to quantify the influence of the adopted testing-for-isolation strategy on infection profiles, and further on the basic reproduction number, to lay a foundation for reverse engineering the effective reproduction number (see Section 5.3).

We consider the infection profile as a discrete function, with calendar time measured in days. Epidemics such as COVID-19 can result in both symptomatic and asymptomatic infections. We define infection profiles separately for symptomatic and asymptomatic infections in a nearly susceptible population. Due to the existence of incubation period where an infected individual is not infectious, consider the day when a symptomatic case becomes infectious as day one. We define ${\underline{v}}_{i}\in R_{\geq 0}$ as the average number of infected cases caused by a single symptomatic case on day *i* since day one, *i* ∈ {1, 2, 3, …, *n*}, where $n\in N_{>0}$ is the number of days during which a symptomatic case is infectious. Hence, the infection profile of symptomatic cases is defined as a vector

$$\begin{array}{c} \underline{v}=\left[{\underline{v}}_{1},{\underline{v}}_{2},\cdots ,{\underline{v}}_{n}\right],\underline{v}\in R^{n}. \end{array}$$ (1)

Similarly, we define ${\bar{v}}_{i}\in R_{\geq 0}$ as the average number of infected cases caused by a single asymptomatic case on day *i* since day one, *i* ∈ {1, 2, 3, …, *m*}, where $m\in N_{>0}$ is the number of days during which an asymptomatic case is infectious. The infection profile of asymptomatic cases is defined as a vector

$$\begin{array}{c} \bar{v}=\left[{\bar{v}}_{1},{\bar{v}}_{2},\cdots ,{\bar{v}}_{m}\right],\bar{v}\in R^{m}. \end{array}$$ (2)

Then, the basic reproduction number of symptomatic and asymptomatic cases can be obtained by

$$\begin{array}{c} \underline{R}=\sum_{i=1}^{n}\;{\underline{v}}_{i}\;\text{and}\;\bar{R}=\sum_{i=1}^{m}\;{\bar{v}}_{i}, \end{array}$$ (3)

respectively [33]. For an epidemic with both symptomatic and asymptomatic infected cases, if *the proportion of the symptomatic infection* is *θ* ∈ [0, 1], then, the proportion of the asymptomatic infection will be (1 − *θ*) ∈ [0, 1]. Consequently, the basic reproduction number of the spreading process is given by

$$\begin{array}{c} R=\theta \sum_{i=1}^{n}\;{\underline{v}}_{i}+\left(1-\theta \right)\sum_{i=1}^{m}\;{\bar{v}}_{i}=\theta \underline{R}+\left(1-\theta \right)\bar{R}. \end{array}$$ (4)

**Fig 2:**
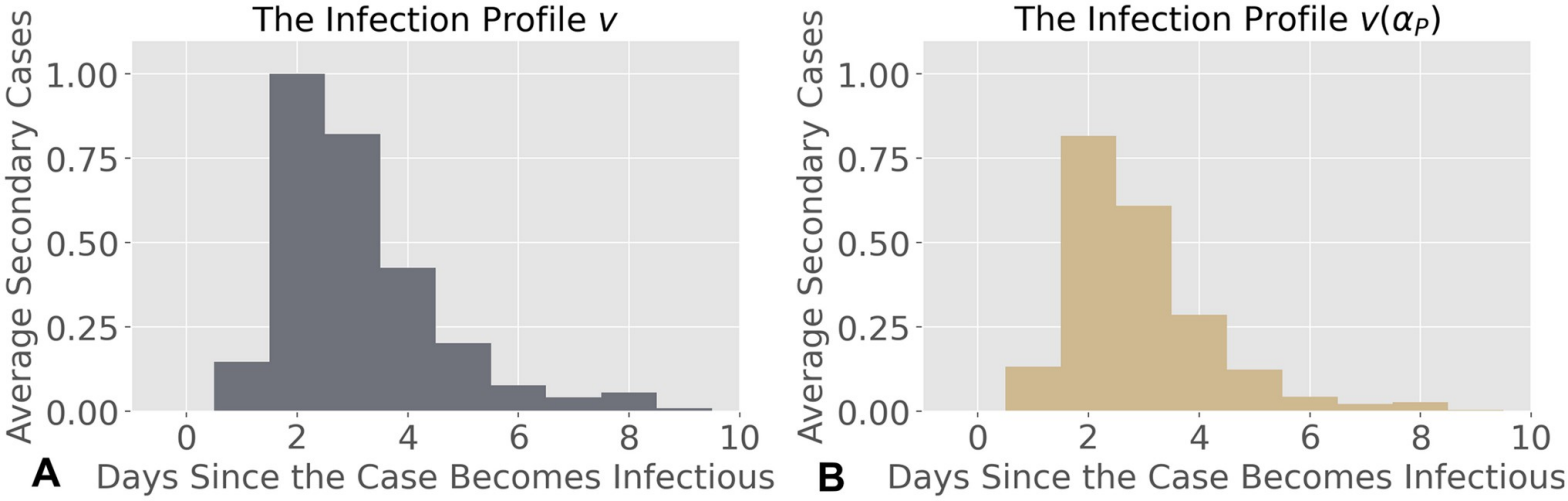
**A)** The infection profile *v* of COVID-19 w/o testing-for-isolation strategies. The infection profile *v* is captured by Eq (5). We leverage the infection profile to capture the spreading process across UIUC and Purdue. **B)** The infection profile *v*(*α*_*P*_) of COVID-19 w/ testing-for-isolation strategies. The infection profile *v*(*α*_*P*_) reflects the impact of the overall isolation rate *α*_*P*_ at Purdue, which reduces the average daily number of infected cases generated by a single infected individual under Purdue’s testing-for-isolation strategy.

We use R to denote the basic reproduction number of a spreading process if the infection profiles $\underline{v}$ and $\bar{v}$ are estimated in a nearly fully susceptible population. In addition, the basic reproduction number R is heavily determined by the ratio of the symptomatic infection *θ*. In this study, we use the same infection profiles for both symptomatic and asymptomatic cases at UIUC and Purdue, as previous research on epidemic spread over the UIUC campus did not differentiate between the infection profiles for these two types of infections [24, 40]. The infection profile is given by

$$\begin{array}{c} \underline{v}=\bar{v}=\left[0.148,1.0,0.823,0.426,0.202,0.078,0.042,0.057,0.009\right], \end{array}$$ (5)

which is shown in Fig 2A.

Different testing-for-isolation strategies, specifically different isolation rates, alter the infection profile in distinct ways. We propose a method to quantify the impact of the daily isolation rate, *α* ∈ [0, 1], on the infection profile (Section 5.1 and Section 2A in S1 Appendix). We implement our method for quantifying the impact of the daily isolation rate on the infection profile with mixed symptomatic and asymptomatic cases (Section 5.1) on the infection profile in Eq (5), according to the testing-for-isolation strategies of UIUC and Purdue, respectively.

We first study the impact of the testing-for-isolation strategy from Purdue on the infection profile in Eq (5). Purdue performed voluntary testing-for-isolation for symptomatic infections and surveillance testing-for-isolation for asymptomatic infections. For simplicity, we consider the daily isolation rate for symptomatic cases, i.e., positive cases that were isolated by voluntary testing, as ${\underline{\alpha }}_{P}=1/7$. Based on the method that quantifies the impact of the daily isolation rate on the infection profile (see Section 5.1), the infection profile of symptomatic cases under the voluntary testing-for-isolation is

$$\begin{array}{c} \underline{v}\left({\underline{\alpha }}_{P}\right)=\left[0.127,0.735,0.518,0.229,0.093,0.031,0.014,0.017,0.002\right]. \end{array}$$

In addition, we consider the daily isolation rate for asymptomatic cases as ${\bar{\alpha }}_{P}=0.3/7$. This condition means that we assume that 30% of the total asymptomatic cases will be tested voluntarily and then isolated throughout the week, evenly split over seven days. We also discuss different isolation rates at Purdue University in Section 2E in S1 Appendix. Based on Eq (7), the infection profile of asymptomatic cases at Purdue is

$$\begin{array}{c} \bar{v}\left({\bar{\alpha }}_{P}\right)=\left[0.147,0.916,0.722,0.356,0.162,0.06,0.03,0.04,0.006\right]. \end{array}$$

Then we generate the basic reproduction number of the combined infection profile of the whole population through Eq (8), shown in Fig 2B. Based on the relationship between the infection profile and the basic reproduction number (Eq (9)), the basic reproduction number under the testing-for-isolation strategy at Purdue is given by $R(\alpha_{P})=2.07$, where $\alpha_{P}=\theta {\underline{\alpha }}_{P}+\left(1-\theta \right){\bar{\alpha }}_{P}$ is defined as the overall isolation rate at Purdue.

The basic reproduction number from the infection profile *v* in Eq (5) is R=2.785. Therefore, the testing-for-isolation strategy implemented by Purdue scales the basic reproduction number by

$$\begin{array}{c} F\left(\alpha_{P}\right)=R\left(\alpha_{P}\right)/R=0.742, \end{array}$$ (6)

where we define $F(\alpha_{P})$ as the *scaling factor* of the basic reproduction number under the overall isolation rate *α*_*P*_. Compared to Purdue, UIUC tested the entire campus twice a week during Fall 2020. We assume that all detected symptomatic and asymptomatic cases can be isolated immediately after testing positive. Therefore, the overall daily isolation rate at UIUC is given by *α*_*I*_ = 2/7. Again, using our proposed method in Section 5.1, we obtain that $R(\alpha_{I})=1.084$, which scales the basic reproduction number down by 38.85%, i.e., $F\left(\alpha_{I}\right)=R\left(\alpha_{I}\right)/R=0.3885$.

Compared to Purdue’s strategy, UIUC’s testing-for-isolation strategy can reduce the basic reproduction number almost twice as much as Purdue’s ($F(\alpha_{P})=0.742$), at the cost of more resources. Therefore, we establish a way to measure the strength of the testing-for-isolation strategy (i.e., the isolation rate) on epidemic spreading processes in terms of modifying the infection profile and the basic reproduction number. We illustrate the results by analyzing the infection profile under Purdue and UIUC’s testing-for-isolation strategies. For further discussion on the testing-for-isolation strategies implemented by Purdue and UIUC, and detailed methods for quantifying their impact on the infection profile and the basic reproduction number, please refer to Section 2C in S1 Appendix.

### 3.2 Estimating the effective reproduction number at UIUC and Purdue

The framework in Fig 1 illustrates that our methods are centered around the reproduction number. Therefore, to leverage the connection between the implemented isolation rate and the basic reproduction number for counterfactual analysis, we first obtain the effective reproduction number from the real-world spreading data at Purdue and UIUC. We leverage the confirmed cases from both universities to estimate the effective reproduction number, as illustrated by the framework depicted within the dashed region in Fig 1. We utilize Bayesian inference techniques and the EpiEstim package [41–43] to estimate the effective reproduction number. Since we leverage existing methods to estimate the effective reproduction number, we only briefly introduce the core idea. Detailed methodologies on estimating the effective reproduction number from confirmed epidemic cases can be found in [19, 33, 41–45], as well as in Sections 2B and 2D in S1 Appendix.

**Fig 3:**
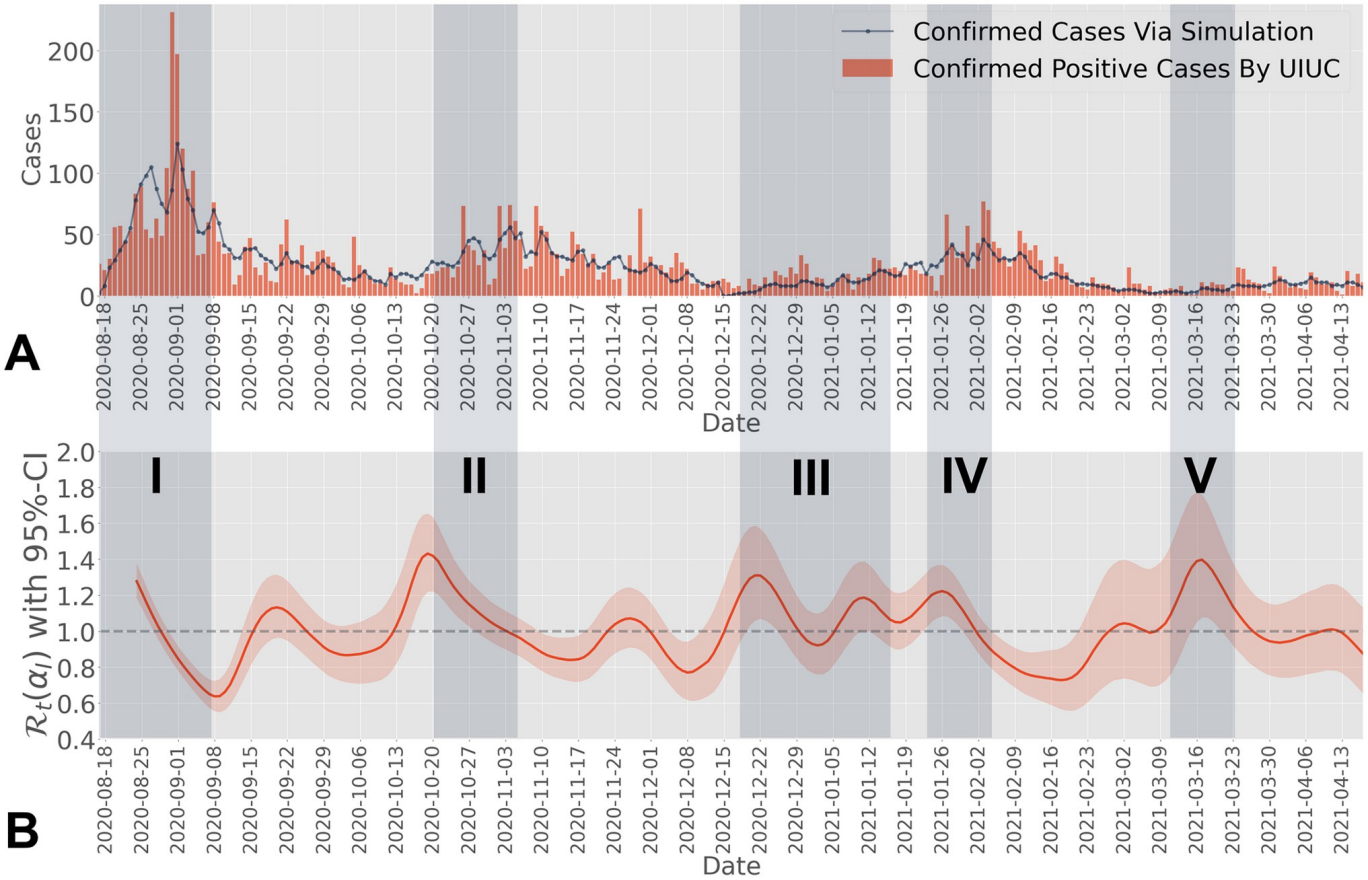
**A)** Daily confirmed cases at UIUC during Fall 2020 and Spring 2021. We mark five shaded areas for five important events across the two semesters, corresponding to the entry screening of Fall 2020 (I), the start of the Big Ten football season (II), Christmas Break (III), the entry screening of Spring 2021 (IV), and Spring Break (V). The simulated spreading process (dotted solid line) accurately captures the spreading trend observed on the UIUC campus during Fall 2020 and Spring 2021, including spikes and weekly confirmed pattern. We simulate the spreading process over Fall 2020 and Spring 2021 separately, since at the beginning of each semester, the entry-screening resets the spreading process. **B)** Estimated effective reproduction number for UIUC from Fall 2020 to Spring 2021. The estimated effective reproduction number (95% confidence interval) is greater than one during multiple periods, particularly at the beginning of Fall 2020, around the middle of October 2020, and the middle of Spring 2021, corresponding to the three events marked by Shaded Areas I, II, and V in Fig 3A. The effective reproduction number aligns with the confirmed cases at UIUC during Fall 2020 and Spring 2021, as shown in Fig 3A, where several mild spikes were observed. There is no estimated effective reproduction number from 2020-08-18 to 2020-08-24 because we use data from the seven-day window between 2020-08-18 and 2020-08-25 to estimate the effective reproduction number for 2020-08-25. We do not have sufficient data to estimate the effective reproduction number prior to 2020-08-25.

We obtain the effective reproduction number for both universities based on the modified serial interval distribution (Section 5.2), accounting for the impact of the testing-for-isolation strategies employed by UIUC and Purdue on the infection profile, respectively. In particular, we leverage the daily confirmed cases from UIUC during Fall 2020 and Spring 2021, ranging from August 18^*th*^ 2020 to April 13^*th*^ 2021 (Fig 3A), to estimate the effective reproduction number $R_{t}\left(\alpha_{I}\right)$ under the overall isolation rate *α*_*I*_, as illustrated in Fig 3B, along with the 95% confidence interval. One key observation during Fall 2020 is that there were two periods where the effective reproduction number (95% confidence interval) was above one, matching the two major events during that semester in the confirmed cases in Fig 3A. The most notable spike during Fall 2020 in Fig 3A was during the period from August 18^*th*^, 2020 to August 31^*st*^, 2020, captured by Shaded Area I. At the beginning of the Fall 2020 semester, when students returned to campus, a significant number of infected cases were identified by the SHIELD team. Additionally, the estimated effective reproduction number experienced a significant spike during mid-October to early November. Research by the SHIELD team at UIUC attributed this increase to the return of the Big Ten football season, when students violated the social distancing policy and began to gather, marked by the Shaded Area II in Fig 3A. There is a delay between the spikes in confirmed cases and the estimated effective reproduction number due to factors such as the incubation period, testing-to-confirmation delay, and the selection of the estimation window, which leverages past data. Further discussion on the impact of these delays on the estimation of the effective reproduction number can be found in Section 2D in S1 Appendix.

**Fig 4:**
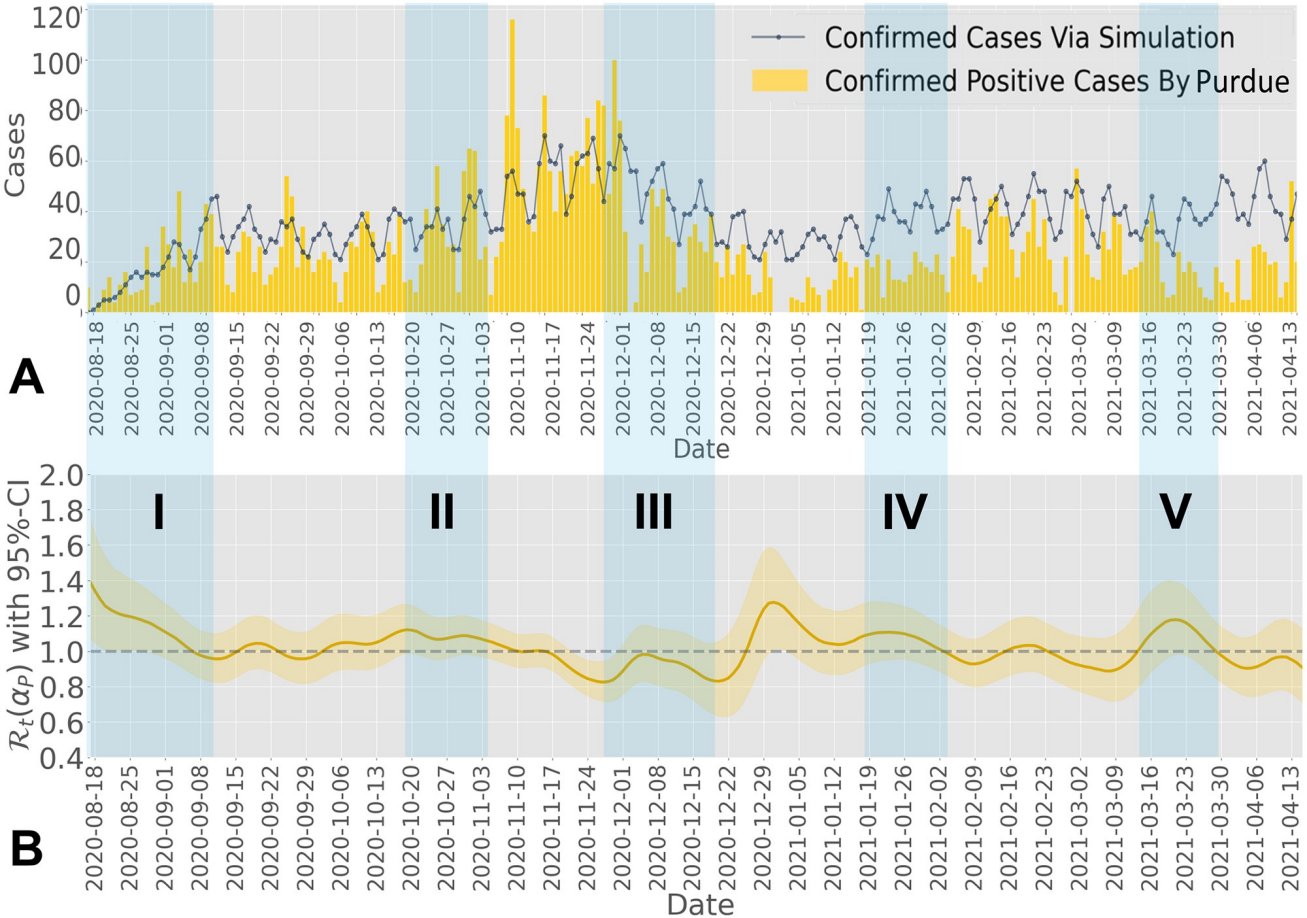
**A)** Daily confirmed cases at Purdue during Fall 2020 and Spring 2021. We mark five shaded areas for five major events across the two semesters at Purdue, corresponding to the entry screening of Fall 2020 (I), the start of the Big Ten football season (II), Thanksgiving Break (III), the entry screening of Spring 2021 (IV), and Spring Break (V). Purdue allowed students to stay home after Thanksgiving break, leading to a significant decrease in confirmed cases, as shown in Shaded Area III. The reconstructed spreading process (dotted solid line) matches the confirmed cases observed on the Purdue campus during Fall 2020 and Spring 2021. Unlike UIUC, where we reset the initial condition at the start of Spring 2021, not resetting it for Purdue results in overestimating daily cases during Spring 2021. **B)** Estimated effective reproduction number for Purdue from Fall 2020 to Spring 2021. The estimated effective reproduction number (95% confidence interval) was around one for most of the time, reflecting that Purdue’s testing-for-isolation strategy avoided potential large outbreaks. Two major spikes were observed in the estimated effective reproduction number (95% confidence interval) at Purdue: one around the beginning of August (I) and the other around the beginning of January (IV). As shown in Fig 4A, these two spikes correspond to the infection process during the Summer and Christmas breaks. Unlike UIUC, Purdue provided sufficient data prior to 2020-08-18, allowing us to estimate the effective reproduction number from 2020-08-18 to 2020-08-24, as shown in Fig 4B. To match the dates in both figures, we did not include data prior to 2020-08-18 in Fig 4A.

Similar to UIUC, we leverage confirmed cases from Fall 2020 to Spring 2021 at Purdue University via the IDA+A team, as shown in Fig 4A, to estimate the effective reproduction number. The confirmed cases include the total number of confirmed symptomatic and asymptomatic cases. The daily confirmed cases through voluntary testing and surveillance testing can be found in Sections 1B and 1C in S1 Appendix. Fig 4A shows five major events by shaded areas: from August 18^*th*^ to September 10^*th*^, from October 13^*th*^ to November 15^*th*^, from November 27^*th*^ to December 20^*th*^, from January 16^*th*^ to February 1^*st*^, and from March 16^*th*^ to March 30^*th*^. These five major events correspond to the entry screening of the Fall 2020 semester (Shaded Area I), the increasing number of gatherings and activities at the start of the Big Ten football season (Shaded Area II), the Thanksgiving Break (Shaded Area III), the entry screening of the Spring 2021 semester (Shaded Area IV), and the return from 2021 Spring break (Shaded Area V), respectively. We leverage the total confirmed positive cases shown in Fig 4A, to estimate the effective reproduction number $R_{t}\left(\alpha_{P}\right)$ under the overall isolation rate *α*_*P*_, as illustrated in Fig 4B. From the estimation, we observe that the estimated effective reproduction number (95% confidence interval) fluctuated around one, and the pattern corresponds to the five major events shown in the shaded areas in Fig 4A. For example, the estimated effective reproduction number (95% confidence interval) dropped below one at the end of November due to Purdue allowing most students to stay home starting from Thanksgiving break until the end of the Fall 2020 semester.

Although UIUC and Purdue implemented different testing-for-isolation strategies, the estimated effective reproduction number reflects similar spreading trends in terms of entry-screening and in-semester spikes. One main reason for this observation is that both universities have a similar size, population behavior, and location. In addition to leveraging the estimated effective reproduction number to analyze the epidemic spread, as illustrated in Figs 3 and 4, we find that the estimated effective reproduction number fluctuated around one at both universities. Since we propose a method to measure the impact of the isolation rate on the basic reproduction number (Section 3.1), we further establish an algorithm to leverage the isolation rate as a control variable to manipulate the effective reproduction number directly (see Section 5.1). The effective testing-for-isolation strategies implemented by both universities inspire our closed-loop feedback control algorithm, where the mitigation goal is to maintain the effective reproduction number at a desired threshold, i.e., less than or equal to one.

### 3.3 Reconstructing epidemic spread at UIUC and Purdue

Through the estimated effective reproduction number, we simulate the spreading process, as shown in the Reconstruction framework in Fig 1. This simulation process, which uses the effective reproduction number to model the spread, forms the basis for leveraging the reverse engineered effective reproduction number to simulate the hypothetical spread. One challenge faced by researchers in modeling, analyzing, and controlling epidemic spreading processes is the fact that such processes are irreversible. We cannot experience the exact same epidemic spreading process under the exact same conditions twice. Therefore, to evaluate the effectiveness of existing intervention strategies and to create the hypothetical spreading scenario to assess the impact of different strengths of the intervention strategies on the epidemic spread, we leverage the methodology introduced by [41, 42] and the estimated effective reproduction number in Section 3.2 to reconstruct the spreading process.

Reconstructing the epidemic spread through the estimated effective reproduction number can be formulated as the inverse process of estimating the effective reproduction number through the confirmed cases. Since we leverage existing methods from [41, 42] to reconstruct the spreading process, and the methodologies for simulating the spreading process from the effective reproduction number are not the main focus of this paper, we only present the results in this section. In Section 2B in S1 Appendix, we leverage the infection profile, the simulated effective reproduction number, delay distributions, and added noisy patterns to show that we can generate both infected cases (without any delays and noise) and confirmed cases (by adding different delays and weekly noisy patterns) to imitate confirmed cases with weekly reporting patterns for a real-world spread. Therefore, we use this method to simulate the confirmed cases at both universities by leveraging the estimated effective reproduction numbers given in Section 3.2. More detailed discussions on how to reconstruct the spreading process using the effective reproduction number, the serial interval distribution, and delay distributions can be found in Section 2B in S1 Appendix and [41, 42].

Through the estimated effective reproduction number in Figs 3B and 4B, we obtain the reconstructed spreading processes on both UIUC and Purdue campuses in the form of daily confirmed cases, depicted by the solid lines in Figs 3A and 4A, respectively. The reconstructed spreading processes can successfully capture the epidemic spreading trend over both campuses, especially the spikes (see Section 2E in S1 Appendix). Building on this reconstruction methodology, in the following sections, we simulate hypothetical spreading scenarios at UIUC and Purdue. These scenarios assume that alternative isolation rates were implemented while nothing else changes in the hypothetical spreading scenario, meaning that the change in the strength of the intervention strategy does not further impact students’ behavior, viral loads, or other factors. Using the reverse engineering method for the effective reproduction number in Section 5.3, we apply varying intensities of isolation rates. Through these simulations, we evaluate the effectiveness of the implemented isolation rates and explore alternative strengths of testing-for-isolation strategies. Note that based on the spreading behavior of COVID-19, we can implement alternative reconstruction methods, such as a Susceptible-Exposed-Infectious-Recovered (SEIR) model, to simulate the spread, where the spreading behavior can be captured by the time-varying parameters in the model.

### 3.4 Evaluating isolation strategies at UIUC and Purdue

Following the framework in Fig 1, along with the estimated effective reproduction numbers from UIUC and Purdue in Section 3.2 and the reconstructed spreading processes in Section 3.3, we illustrate the method for reverse engineering the effective reproduction number with alternative strengths of the implemented testing-for-isolation strategy or without any isolation at all (see Section 5.3). We show that this method allows us to perform counterfactual analysis to evaluate what would have happened if UIUC and Purdue had not implemented their testing-for-isolation strategies, under the assumption that the change in the strength of the intervention strategy does not further impact students’ behavior, viral loads, or other factors.

For the outbreaks at UIUC and Purdue, consider that the only difference between 1) the spread without any testing-for-isolation strategies and 2) the historical spreading process is the implemented isolation rate. Furthermore, we assume the population on the two university campuses is finite and remains fixed during the semester, and there is no loss of immunity after recovery in such a short period. We first evaluate the implemented testing-for-isolation strategy on the UIUC campus. We leverage the reconstruction method that uses the effective reproduction number to simulate the spread, as described in Section 3.3, in order to simulate the hypothetical spreading scenario on the UIUC campus. In this scenario, the effective reproduction number for the spread without the isolation strategy is computed using Eq (13), based on the estimated reproduction number from UIUC in Fig 4A.

When examining the hypothetical spreading scenario on the UIUC campus without any implemented isolation strategies, we consider the worst-case scenario. In this scenario, the implemented daily isolation rate is *α*_*I*_ = 2/7 = 0.286, indicating that the isolation rate is equal to the testing rate, i.e., testing and isolating all positive cases twice a week. Further, without the testing-for-isolation strategy, both symptomatic and asymptomatic cases will behave normally and will not isolate themselves from the population. This worst-case scenario creates a situation where no one on campus takes preventative action against the pandemic. Although no isolation measures are in place, we can still record confirmed cases using the same testing strategy implemented by UIUC, facilitating the estimation of the susceptible population on campus (see Section 5.3). The detailed process on reconstructing the hypothetical spreading process on the UIUC campus without their testing-for-isolation strategy can be found in Section 2E in S1 Appendix.

**Fig 5:**
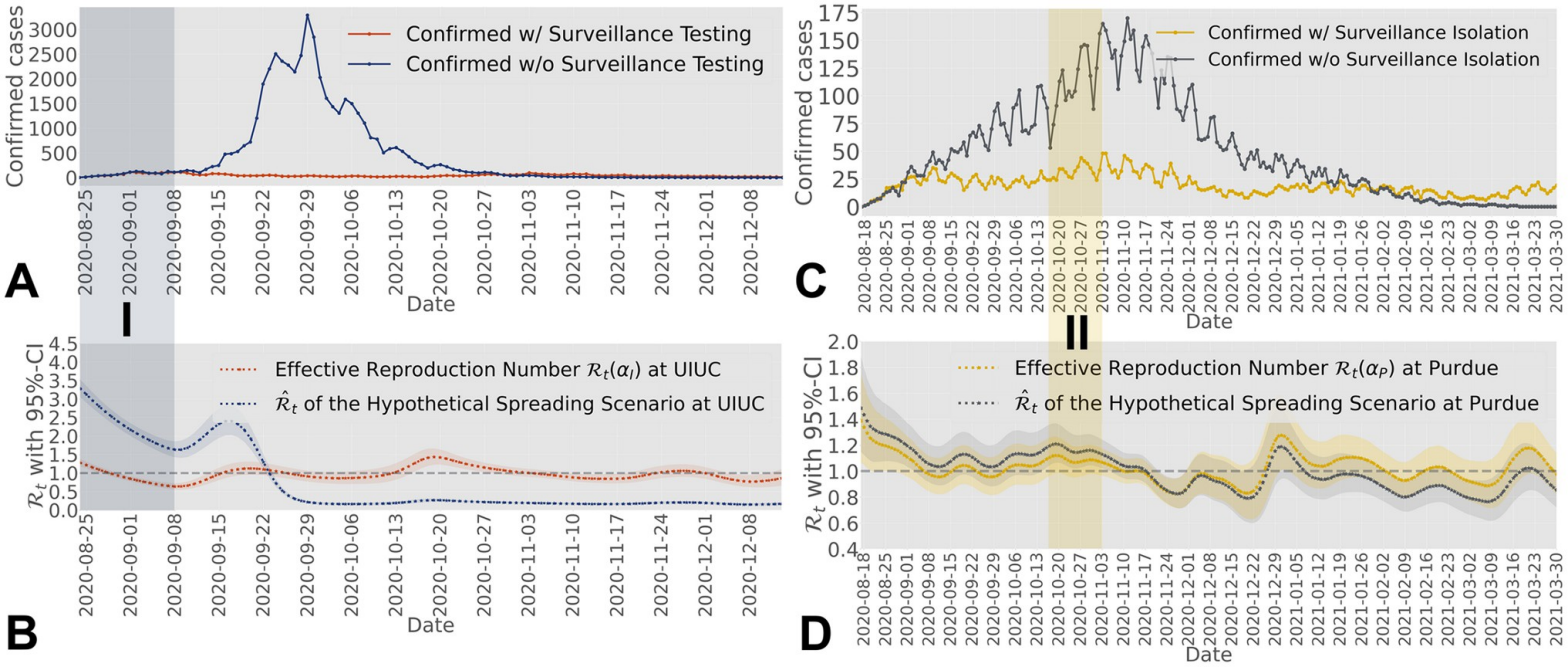
**A)** Daily confirmed positive cases from hypothetical spreading scenario at UIUC during Fall 2020. The solid dark blue line represents the hypothetical spreading scenario without employing any testing-for-isolation strategies. The solid red line represents the simulated spreading process under UIUC’s testing-for-isolation strategy. The hypothetical spreading scenario captures the worst-case scenario where every individual on campus does not take actions against the pandemic. **B)** Reverse engineered effective reproduction number at UIUC. The dashed dark blue line illustrates the reverse engineered effective reproduction number of the hypothetical spreading scenario without the implementation of testing-for-isolation strategies. The dashed red line represents the estimated effective reproduction number obtained from the data at UIUC featuring the implemented testing-for-isolation strategy. **C)** Daily confirmed positive cases from hypothetical spreading scenario at Purdue during Fall 2020 and Spring 2021. Compared to the hypothetical spreading scenario at UIUC, the simulated outbreak at Purdue is less severe since, by assumption, all symptomatic cases are caught and isolated through the voluntary testing-for-isolation strategy. **D)** Reverse engineered effective reproduction number at Purdue. During the first two months of the Fall 2020 semester, the effective reproduction number of the hypothetical spreading scenario without isolation under surveillance testing consistently exceeds the estimated effective reproduction number obtained from the real spreading data on campus. After October 2020, the reduction in the susceptible population leads to the effective reproduction number of the hypothetical spreading scenario becoming smaller than the estimated effective reproduction number of the COVID-19 data from Purdue.

Fig 5A shows that, without any isolation and further with everyone taking no actions against the virus, there would have been a significant outbreak on the UIUC campus during Fall 2020. Around 90% of the total population on campus will be infected during the Fall 2020 semester. Starting from September 2020, as captured by the Shaded Area I in Fig 5A, the confirmed cases of the hypothetical spreading scenario start to grow slowly, since the strict entry-screen caught most of the infected cases. However, even with the pandemic interventions implemented by UIUC, there was a mild spike in mid-September, reflected by the peak value of the estimated effective reproduction number between Shaded Area I and Shaded Area II in Fig 3B. For the hypothetical spreading scenario without any isolation strategies, the effective reproduction number around mid-September would be further scaled up (see Eq (13)), as shown in Fig 5B. Consequently, the effective reproduction number in this hypothetical scenario would rise, causing the number of confirmed cases to continue increasing from mid-September, eventually peaking around the end of September.

Later on, the confirmed cases start to decrease, which is caused by a heavily reduced susceptible population on campus during Fall 2020 (see Eq (13)). Therefore, the campus reaches the herd immunity threshold [46, 47], where the epidemic begins to fade away after a certain portion of the population becomes infected and gains immunity against the virus. Additionally, the peak value is enormous because we consider no isolation or intervention for the infected cases, and the change in the strength of the intervention strategy does not further affect other factors, making it essentially the worst-case scenario. This scenario can be demonstrated by a similar large infection peak in China during Spring 2023 when most COVID-19 interventions were suddenly lifted, allowing the virus to spread freely [48]. More counterfactual analyses are explored by considering different isolation rates on the UIUC campus in Section 2E in S1 Appendix.

After discussing what would have happened without the implemented testing-for-isolation strategies, we compare 1) the effective reproduction number under reverse engineering (Fig 5B blue line) with 2) the estimated effective reproduction number (Fig 5B red line) to illustrate the method in Section 5.3. Fig 5B shows that starting from the beginning of the Fall 2020 semester until late September 2020, the effective reproduction number of the hypothetical spreading scenario ${\hat{R}}_{t}$ is higher than the estimated effective reproduction number $R_{t}\left(\alpha_{I}\right)$, reflected by the exponential growth before the end of September. This phenomenon is determined by the scaling factor $F(\alpha_{I})$ in the reverse engineering method (Eq (13) in Section 5.3). As explained in Eq (13), another determining factor for reverse engineering the effective reproduction number is the ratio between the susceptible populations. Fig 5B illustrates that after a large amount of the population on campus is infected, the infected population starts to decrease. Therefore, from late September 2020, the effective reproduction number of the hypothetical spreading scenario ${\hat{R}}_{t}$ is lower than the estimated effective reproduction number of the real-world spread $R_{t}\left(\alpha_{I}\right)$.

Using the same method from Section 5.3 and the estimated reproduction number, $R_{t}\left(\alpha_{P}\right)$, from Purdue (as shown in Fig 4B), we reconstruct the hypothetical spreading scenario for the Purdue campus without the testing-for-isolation strategies that were implemented. As discussed regarding the impact of Purdue’s testing-for-isolation strategy on the infection profile, all confirmed symptomatic cases will self-report and isolate themselves from the population when they test positive, as they are cautious and willing to be tested. Based on the testing data from Purdue, we have the ratio of the symptomatic infection to be *θ* = 55%. In contrast to UIUC, we focus on the impact of the surveillance testing-for-isolation strategy on asymptomatic cases at the Purdue campus, i.e., the daily isolation rate for asymptomatic infection only, while fixing the daily isolation rate for symptomatic infection at 1/7. In addition, we consider a daily 0.3/7 isolation rate for asymptomatic infection when simulating the spreading process over the Purdue campus. For further discussion on the impact of choosing the isolation rate and the symptomatic ratio, refer to Section 2E in S1 Appendix.

We reconstruct the hypothetical spreading scenario over the Purdue campus without the testing-for-isolation strategy for asymptomatic infections during Fall 2020 and Spring 2021, as illustrated in Fig 5C. Fig 5C shows that without the surveillance testing-for-isolation strategy that generates a daily isolation rate of 0.3/7 for the asymptomatic infected population, and under the condition that all symptomatic cases will self-report and isolate themselves from the population, there would be a larger outbreak. Due to the existence of *θ* = 55% symptomatic population being isolated, the hypothetical outbreak is much milder than that of UIUC. In particular, the confirmed cases of the hypothetical spreading scenario would start to surpass the confirmed cases at Purdue beginning in October, caused by the return of the Big Ten football season (the Shaded Area II in Fig 5C). Additionally, Fig 5D indicates that the effective reproduction number of the hypothetical spread ${\hat{R}}_{t}$ is slightly higher than the estimated effective reproduction number $R_{t}\left(\alpha_{P}\right)$ from the beginning of the Fall 2020 semester until the end of October. We can also explain this phenomenon by the absence of the testing-for-isolation strategy for asymptomatic cases (see Eq (13)).

In this section, we demonstrate the proposed method of reverse engineering the effective reproduction number (as detailed in Section 5.3) through our counterfactual analysis to evaluate the testing-for-isolation strategies implemented by UIUC and Purdue. The evaluation shows that the testing-for-isolation is critical for epidemic mitigation. Without testing-for-isolation, there would have been a huge outbreak, as illustrated by the analysis of the hypothetical spreading scenario over the UIUC campus. Even under the ideal situation where all symptomatic cases are tested voluntarily and isolate themselves, there still would have been an outbreak due to the existence of asymptomatic cases and disturbances such as the Big Ten football season, as illustrated by the hypothetical spreading scenario on the Purdue campus. We further discuss additional evaluations of the implemented testing-for-isolation strategies over the UIUC and Purdue campuses under different scenarios in Section 2E in S1 Appendix.

### 3.5 Open-loop epidemic control

We have evaluated the spreading processes on both the UIUC and Purdue campuses by introducing a method to reverse engineer the effective reproduction number for the hypothetical spreading scenario without the implemented isolation strategy, addressing the Epidemic Reconstruction and Strategy Evaluation phase in Fig 1. To further address the Strategy Evaluation framework, we illustrate the reverse engineering method from Section 5.3 by conducting a counterfactual analysis on the hypothetical spreading scenario under different fixed isolation rates for UIUC and Purdue. The selection of a fixed isolation rate, regardless of the spread, can be viewed as an open-loop epidemic control strategy.

First, we study the worst-case hypothetical spread over the UIUC campus under different isolation rates, as given in Fig 5A. When discussing control strategies, we describe the isolation rates on a weekly basis to align with the universities’ testing-for-isolation policy. In practice, adjusting the isolation rate daily can be challenging. However, for scaling the infection profile and the basic reproduction number in Eq (6), we still utilize the daily isolation rate, which is obtained by dividing the weekly isolation rate by seven. In the hypothetical worst-case scenario, if an infected case is not isolated after testing, the individual will behave normally, as if uninfected, until recovery. We compare the outcomes if we had implemented different fixed isolation rates that are less than 200% weekly on the UIUC campus during Fall 2020. Under the condition that the weekly testing rate is 200%, the fixed weekly isolation rates of the hypothetical spreading scenario are drawn from {0%, 10%, 20%, …, 90%} and {100%, 120%, …, 180%, 200%} in order. Meanwhile, the testing-for-isolation process does not distinguish between symptomatic and asymptomatic cases.

**Fig 6:**
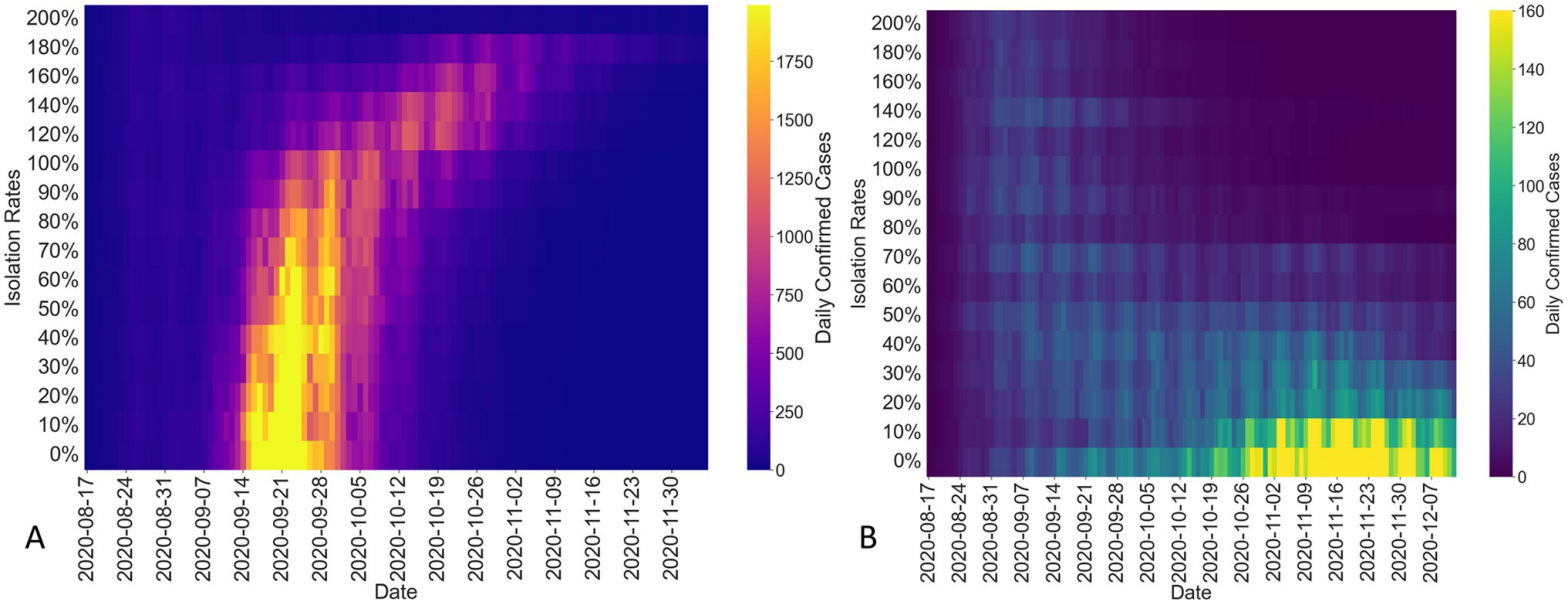
A) Daily confirmed cases over the UIUC campus with different isolation rates. The fixed weekly isolation rates of the hypothetical spreading scenario are drawn from {0%, 10%, 20%, …, 90%} and {100%, 120%, …, 180%, 200%}. Higher isolation rates will result in relatively smoother and flatter curves in terms of outbreak. The shape of the brighter area also indicates that a higher isolation rate will lead to lower spikes, while these lower spikes will also be further delayed. B) Daily confirmed cases over Purdue campus with different isolation rates. The weekly isolation rates for asymptomatic cases are drawn from {0%, 10%, 20%, …, 90%} and {100%, 120%, …, 180%, 200%}. The daily confirmed cases of the hypothetical outbreak at Purdue are significantly lower than those on the UIUC campus, due to the existence of a voluntary testing-for-isolation strategy. Higher isolation rates generate relatively smoother and flatter curves in terms of confirmed cases.

According to the reverse engineering method for the effective reproduction number, used for hypothetical analysis of the spread with alternative isolation rates (Eq (14)), and the framework in Section 3.3, we generate the daily confirmed cases at UIUC shown in the heatmap in Fig 6A. The heatmap is based on the reverse engineered effective reproduction number under different isolation rates and it highlights higher peak infection levels with brighter colors. Fig 6A suggests that higher isolation rates lead to smoother and flatter curves in terms of confirmed cases in the hypothetical spreading scenario, indicating a reduced peak in infection levels. The shape of the brighter area in Fig 6A also indicates that a higher isolation rate will lead to lower spikes, while these lower spikes will also be further delayed. All analyses are based on the hypothetical worst-case scenario at UIUC proposed in Section 3.3 and the assumption that the change in the isolation rate will not affect students’ behavior, viral loads, or other factors. Assessing these fixed isolation rates in a different hypothetical spreading environment at UIUC would yield significantly different results. Therefore, when evaluating intervention strategies such as a fixed isolation rate, it is critical to consider assumptions regarding population dynamics and virus transmission behavior. However, the proposed reverse engineering method for the effective reproduction number remains general, regardless of these assumptions. We present the impact of the isolation rate on the cumulative confirmed cases in Section 2F in S1 Appendix.

In comparison to UIUC, we analyze the effects of varying isolation rates under surveillance testing for the hypothetical spreading scenario on the Purdue campus. We use the hypothetical spreading environment at Purdue during Fall 2020 without the implemented surveillance testing-for-isolation, as shown in Fig 5C, where positive cases confirmed through voluntary testing would self-isolate. Further, based on the data from Purdue, *θ* = 55% of the population was symptomatic. We vary the weekly isolation rate from the surveillance testing for asymptomatic cases from {0%, 10%, 20%, …, 90%} and {100%, 120%, …, 180%, 200%}. Using the same methods and assumptions applied to study alternative isolation rates on the hypothetical spreading scenario on the UIUC campus, we capture the daily confirmed cases for Purdue as shown in the heatmap in Fig 6B. We obtain the confirmed cases based on reconstructing the hypothetical spreading scenarios using the reverse engineered effective reproduction numbers under different isolation rates.

As expected, the daily confirmed cases of the hypothetical spreading scenario at Purdue were much lower than the daily confirmed cases on the UIUC campus, given the extra assumption regarding the population behavior of symptomatic cases at Purdue. Higher isolation rates generate relatively smoother and flatter curves in terms of confirmed cases, illustrating Eq (14). Additionally, Fig 6B indicates that higher isolation rates result in lower spikes during the return of the Big Ten football season, marked by the Shaded Area II in Fig 5C. Fig 6B also shows that there is a noticeable difference between isolation rates for asymptomatic cases below 50% per week and isolation rates higher than 50% of the asymptomatic cases per week. Based on the analysis in this specific example, when the weekly isolation rate for asymptomatic cases is higher than 50%, there would not be any significant outbreak during the Fall 2020 semester at the Purdue campus. The same as the UIUC study, the simulation results are based on certain conditions and assumptions. Changing simulation conditions and assumptions will generate different conclusions. Further detailed analyses and confirmed cases for Purdue during Fall 2020 under different isolation rates can be found in Section 2F in S1 Appendix.

By studying the impact of varying isolation rates on the hypothetical spreading scenario on the UIUC and Purdue campuses, we illustrate the reverse engineering method for the effective reproduction number under alternative strengths of the isolation rate (see Section 5.3). We formulate the problem by considering an open-loop mitigation strategy with a fixed isolation rate and explore hypothetical scenarios for UIUC and Purdue campuses during Fall 2020. Specifically, we identify how the isolation rate influences peak infection levels and timing. Furthermore, we investigate threshold conditions associated with isolation rates that help avoid potential outbreaks. The conditions set for the hypothetical spreading environment, along with the assumptions about population behavior, viral load, and other factors, will affect the simulation results. Although we cannot alter the real-world spreading process that occurred under the implemented testing-for-isolation strategies, our counterfactual analysis offers valuable insights. By using spreading data and the reverse engineering method from Section 5.3, we can determine appropriate isolation rates and threshold conditions. These insights are critical for policy-making in epidemic mitigation.

### 3.6 Closed-loop feedback epidemic control

In this section, we complete the framework in Fig 1 by implementing a feedback control algorithm to adjust the strength of the testing-for-isolation strategy based on varying isolation rate, which is determined by the severity of the spread, captured by the estimated effective reproduction number. We propose this algorithm in Section 5.4. If all the conditions of the real-world spread from Eq (4) (the infection profiles and the ratio of the symptomatic infection) were perfectly known, it would be possible to generate an isolation rate that maintains the effective reproduction number exactly at the desired value with a single computation, according to our developed mechanisms in Section 5.1. However, the complex nature of the spread introduces uncertainty, making it difficult to design such a rate with perfect settings. To address this challenge, instead of relying on a fixed isolation rate, as studied in Section 3.5, we propose a closed-loop feedback control algorithm to adjust the isolation rate according to the severity of the spread.

The feedback control strategy is straightforward: in order to minimize the total number of tests conducted (which is proportional to the isolation rate) while maintaining the effective reproduction number at a certain level $R_{t}^{*}\in \left(0,1\right]$; we increase the isolation rate to prevent more infected individuals from spreading the virus when the outbreak is severe and decrease the rate to isolate fewer infected individuals when the spread is less severe. We leverage the estimated effective reproduction number as feedback information to indicate the severity of the outbreak. We implement this algorithm in the hypothetical spreading scenario across the UIUC campus, as shown in Fig 5A. A detailed explanation of the closed-loop feedback control algorithm can be found in Section 5.4. We further discuss in Section 2G in S1 Appendix how the goal of maintaining the daily infected population at an acceptable level aligns with the optimal mitigation strategy [49, 50].

Consider implementing the closed-loop feedback control algorithm in the hypothetical spreading scenario in Fig 5A. The control strategy adjusts the weekly isolation rate, which is evenly split over seven days, every two weeks. We estimate the effective reproduction number over the past two weeks and update the isolation rate for the following two weeks. This algorithm (see Section 5.4) indicates that we use the average estimated effective reproduction number of the past 14-day period as the indicator of the effective reproduction number for the subsequent two weeks. The algorithm assumes that the average effective reproduction number for the next two weeks will remain the same as the average value from the past 14-day period, provided the implemented isolation rate does not change. Therefore, there are no prediction mechanisms when updating the future isolation rate.

**Fig 7:**
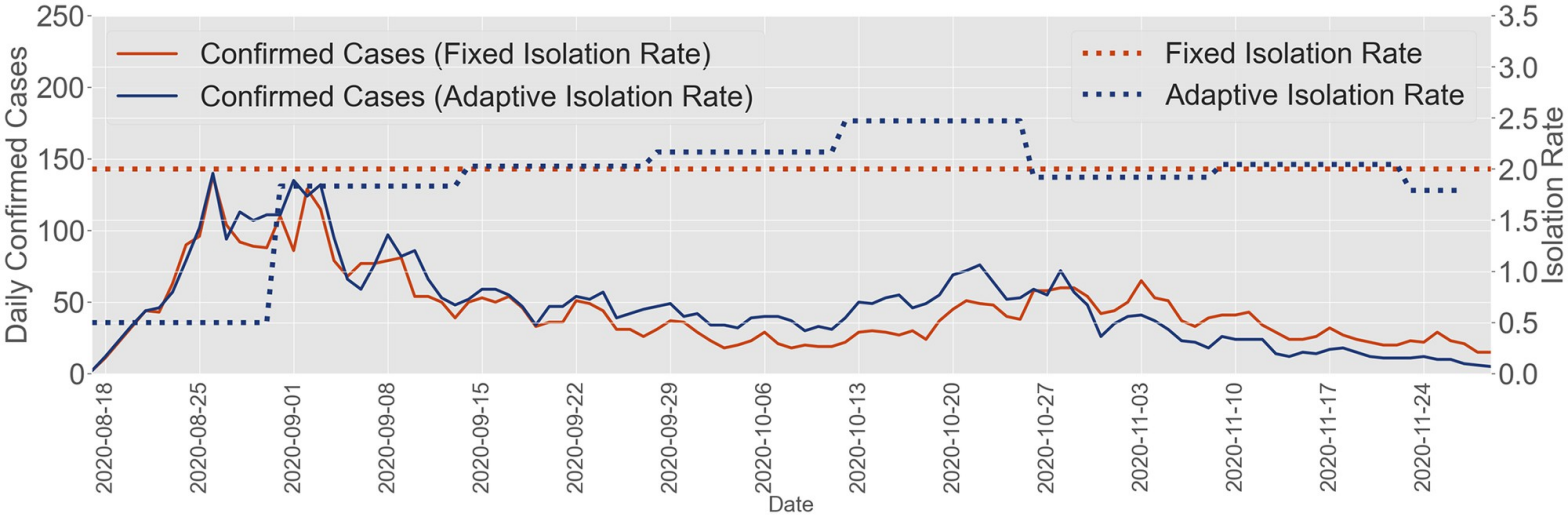
Closed-loop feedback control for the outbreak at UIUC. When controlling the effective reproduction number at 0.95, the closed-loop feedback control algorithm we propose aligns with the testing-for-isolation policy implemented by UIUC in terms of daily and total confirmed cases, which are around 5000. Under the condition that the isolation rate is proportional to the number of tests, the implemented testing-for-isolation strategy by UIUC will result in a total of 32 tests per individual, whereas our proposed feedback control strategy will require 28 tests per individual.

We implement the proposed feedback control algorithm (Section 5.4) in the hypothetical spreading environment in Fig 5A and compare it to the testing-for-isolation strategy implemented by UIUC, which involved testing and then isolating the infectious cases across the entire campus twice a week, i.e., the daily isolation rate will be 2/7. Our goal is to control the effective reproduction number at $R_{t}^{*}=0.95$. The effective reproduction number, slightly smaller than one, ensures that the epidemic can gradually fade away with sufficient testing-for-isolation resource. Controlling the effective reproduction number slightly below one to design pandemic mitigation policies is a strategy discussed by other researchers as well [51, 52]. The feedback control framework, as depicted in Fig 7, demonstrates that it can achieve a similar number of daily and total confirmed cases in the hypothetical spreading scenario (both around 5000) compared to the policy implemented by UIUC. Furthermore, under the condition that the isolation rate is proportional to the number of tests, we find that the implemented strategy by UIUC required testing everyone 32 times in total, while our proposed feedback control strategy only requires testing everyone 28 times in total. Additionally, for most of the Fall 2020 period, the feedback control algorithm implements a lower isolation rate compared to UIUC’s implemented 200% weekly isolation rate. However, during October, the feedback control framework employs higher isolation rates to mitigate the potential outbreak associated with the return of the Big Ten football season. This adjustment is based on the real-world confirmed data and the estimated effective reproduction number during Fall 2020, as depicted in the Shaded Area II in Fig 3A and 3B, respectively.

The feedback control algorithm demonstrates the core concept of utilizing the effective reproduction number as both the feedback information and the control goal to design the closed-loop control algorithm for pandemic mitigation, as illustrated in Fig 1. The strategy adjusts the isolation rate based on the risk of outbreaks, lowering the isolation rate when the risk is low and increasing it when a potential spike is detected. This example highlights the flexibility and effectiveness of the feedback control algorithm in managing pandemics. In Section 2G in S1 Appendix, we further discuss and study the impact of selecting other target effective reproduction numbers (e.g., $R_{t}^{*}=0.90$) on the hypothetical outbreak at UIUC by implementing the closed-loop control algorithm. In addition, we extend the closed-loop control algorithm introduced in Section 5.4 (Eq (15)), accounting for both symptomatic and asymptomatic cases, to design the closed-loop feedback control algorithm for the outbreak on the Purdue campus in Section 2G in S1 Appendix.

## 4 Discussions

### 4.1 Counterfactual analysis, strategy evaluation, and feedback control

We propose a framework for counterfactual analysis, strategy evaluation, and feedback control of epidemics. We propose three methods centered around the reproduction number. The first method quantifies the impact of the testing-for-isolation strategy, specifically, the isolation rate, on the basic reproduction number. This method forms the foundation for the other two methods. The second method involves reverse engineering the effective reproduction number under an alternative strength of the isolation rate. The third method formulates a closed-loop feedback control algorithm that leverages the effective reproduction number as feedback information to guide adjustments to the isolation rate. Our approaches rely on critical information from disease spread such as the infection profile of the spread, the ratio of the symptomatic infection, and the isolation rate, to guide the analysis and control of spreading behavior. We validate the approaches by leveraging testing-for-isolation data from UIUC and Purdue. Through analysis, we evaluate the implemented strategies at both universities during the early stage of the COVID-19 pandemic.

We validate the proposed closed-loop feedback control algorithm that relies on the severity of the spread, indicated by the effective reproduction number. We compare the hypothetical spreading scenario where we implement the feedback control algorithm to adjust the isolation rate, with the fixed isolate rate implemented by UIUC. Our closed-loop feedback control framework effectively manages the spreading process and can adapt to changing conditions. Nevertheless, relying solely on the estimated effective reproduction number, particularly the mean, as feedback information for comprehensive pandemic evaluation may present several limitations and challenges in real-world applications. As noted by the SHIELD team at UIUC, even when the estimated effective reproduction number is around one, outbreaks can still occur on campus [24]. In our closed-loop feedback control algorithm, when selecting a target effective reproduction number slightly below one, such as $R_{t}^{*}=0.95$, a large infected population may still lead to outbreaks because the decline in infections could be very slow. Furthermore, the stochastic nature of outbreaks and potential disturbances from various events may cause the effective reproduction number of the outbreak to easily surpass one. Therefore, when implementing the closed-loop feedback control algorithm for pandemic mitigation, it is reasonable to select a target reproduction number significantly below one to ensure the algorithm’s robustness against disturbances, while carefully adapting the control goal based on the evolution of real-world spreading scenarios.

### 4.2 Interventions beyond testing-for-isolation

We leverage COVID-19 data from universities to introduce and demonstrate our proposed framework, as shown in Fig 1. However, our framework is adaptable to other scenarios involving different intervention strategies. The concept of using the effective reproduction number as both the feedback and the control goal necessitates an examination of the relationship between the intervention strategy and the basic reproduction number, grounded in the infection profile [14–16]. For example, we consider a different intervention strategy, such as varying vaccination percentages. To implement the entire framework outlined in Fig 1, it is critical to quantify the impact of vaccination on altering the basic reproduction number. This connection is essential to effectively reverse engineer the effective reproduction number considering various vaccination rates. Hence, by exploring different intervention strategies’ effects, we can generalize the framework and algorithm.

### 4.3 Future work

We acknowledge several limitations of the proposed framework and provide potential avenues for improvement through future work (for more details, see Sections 3 and 4 in S1 Appendix). First, when estimating the effective reproduction number, we use existing infection profiles from the literature. However, it is essential to update these profiles with contact tracing data from testing-for-isolation strategies to account for varying spreading behaviors. By leveraging contact tracing data, we can capture the impact of stochasticity changes for each individual in the infection profile. The influence includes variations in viral load caused by different virus variants and changes in contact behavior due to policies beyond the testing-for-isolation strategy. Also, we utilize the past average estimated effective reproduction number to project future values in our control design, without considering any predictions. Since the estimated effective reproduction number can fluctuate due to various factors, it is crucial to incorporate predictive control mechanisms and machine learning techniques to improve the approaches. Additionally, as for the mitigation goal, with a substantial initial infected population, maintaining the effective reproduction number slightly below one may still lead to a large number of infections. Hence, adjusting the control goal (i.e., the effective reproduction number) of the framework according to different spreading scenarios becomes highly significant [53, 54].

Furthermore, while the feedback control framework can potentially save mitigation resources, frequent policy changes may be impractical. Meanwhile, the policy generated by the feedback control design could exceed the resource capacity during a certain period. Thus, exploring constrained optimization on the method is necessary. Last, we propose the framework and methods using aggregated data. However, the framework can be improved with spatially and heterogeneously spreading data, where we can estimate the effective reproduction number for connected sub-regions and adjust mitigation strategies accordingly by formulating a network optimization problem. Future work can explore a high-resolution distributed strategy evaluation and feedback control framework, by leveraging machine learning techniques like graph learning and causal inference to infer connections between sub-regions.

Nonetheless, we aim for this work to provoke discussions about the role and limitations of leveraging reproduction number estimates in pandemic mitigation, considering both analytical and computational perspectives. We believe that the three methods we propose for epidemic analysis and control can inspire and significantly benefit the development of rigorous strategy evaluation and feedback control across various research fields. For instance, the feedback control algorithm not only serves as a foundation for designing future control frameworks to allocate resources for epidemic mitigation but also lays the groundwork for incorporating control analysis in other biological and ecological dynamic systems.

## 5 Methods

In this section, we introduce the methods we develop to obtain the results in this work. As outlined in Fig 1, we include 1) the method we design to quantify the impact of the isolation rate on the infection profile and the basic reproduction number, 2) the mechanism to reverse engineer the effective reproduction number, and 3) the closed-loop feedback control algorithm that leverages the effective reproduction number as feedback to adjust the isolation rate. Additionally, we introduce the foundations for estimating the effective reproduction number by leveraging Bayesian inference. The Institutional Review Boards (IRBs) for this research were ruled exempt by UIUC (protocol #21216) and Purdue IRB-2020-1683.

### 5.1 Quantifying the impact of isolation on epidemic spread

In order to align with the infection profile defined on a daily basis, we consider the daily isolation rate to be represented by *α*, $\alpha \in R_{\geq 0}$. We propose a mechanism to quantify the impact of the isolation rate on the infection profile and, consequently, on the basic reproduction number. Consider testing-for-isolation strategies for asymptomatic infection, where the infection profile is given by Eq (2). If we have $k\in N_{>0}$ asymptomatic infectious cases on day one, without testing-for-isolation strategies, these *k* infectious cases will generate an average number of $k{\bar{v}}_{i}$ cases on day *i*, *i* ∈ {1, 2, …, *m*}. However, consider the same number of *k* asymptomatic cases under a testing-for-isolation strategy. If we test and then isolate $k\bar{\alpha }$ asymptomatic cases on day one from the *k* asymptomatic cases, there will be $k{\bar{v}}_{1}\left(1-\bar{\alpha }\right)$ new infected cases that are generated by the $k(1-\bar{\alpha })$ cases. On day two, there will be $k{\bar{v}}_{2}{\left(1-\bar{\alpha }\right)}^{2}$ cases generated by $k{\left(1-\bar{\alpha }\right)}^{2}$ infectious asymptomatic cases. Consequently, the new infected cases caused by the original *k* asymptomatic cases are $k{\bar{v}}_{i}{\left(1-\bar{\alpha }\right)}^{i}$ on day *i*, *i* ∈ {1, 2, …, *m*}. Thus, the average number of infected cases generated by a single asymptomatic individual on day *i*, *i* ∈ {1, 2, …, *m*}, is given by $k{\bar{v}}_{i}{\left(1-\bar{\alpha }\right)}^{i}$. We obtain the asymptomatic infection profile under the impact of the daily isolation rate $\bar{\alpha }$, which is given by

$$\begin{array}{c} \bar{v}\left(\bar{\alpha }\right)=\left[{\bar{v}}_{1}\left(1-\bar{\alpha }\right),{\bar{v}}_{2}{\left(1-\bar{\alpha }\right)}^{2},\cdots ,{\bar{v}}_{m}{\left(1-\bar{\alpha }\right)}^{m}\right]. \end{array}$$ (7)

Eq (7) gives the connection between the daily isolation rate and the infection profile on a daily basis, and consequently, the basic reproduction number according to Eq (3). The same mechanism is applicable to modify the infection profile of symptomatic infections, and we use $\underline{\alpha }$ and $\underline{v}\left(\underline{\alpha }\right)$ to represent the daily isolation rate of symptomatic cases and the infection profile of symptomatic infections under the isolation rate, respectively. Similar to the computation of the basic reproduction number of the mixed population in Eq (4), the modified infection profile with both symptomatic and asymptomatic infection of the population is given by

$$\begin{array}{c} v\left(\alpha \right)=\theta \underline{v}\left(\underline{\alpha }\right)+\left(1-\theta \right)\bar{v}\left(\bar{\alpha }\right), \end{array}$$ (8)

quantifying the impact of the overall isolation rate $\alpha =\theta \underline{\alpha }+\left(1-\theta \right)\bar{\alpha }$ on the infection profile *v*. Further, similar to Eq (4), we define the basic reproduction number of the spread under the overall isolation rate *α*, which is a function of the isolation rates $\underline{\alpha }$ for symptomatic infections and $\bar{\alpha }$ for asymptomatic infections, as

$$\begin{array}{c} R\left(\alpha \right)=\theta \sum_{i=1}^{n}{\underline{v}}_{i}\left(\underline{\alpha }\right)+\left(1-\theta \right)\sum_{i=1}^{m}{\bar{v}}_{i}\left(\bar{\alpha }\right)=\theta \underline{R}\left(\underline{\alpha }\right)+\left(1-\theta \right)\bar{R}\left(\bar{\alpha }\right), \end{array}$$ (9)

where $\underline{R}\left(\underline{\alpha }\right)$ and $\bar{R}\left(\bar{\alpha }\right)$ are the basic reproduction numbers of symptomatic and asymptomatic infections under the isolation rates $\underline{\alpha }$ and $\bar{\alpha }$, respectively. Note that the method also applies to the effective reproduction number where infection profiles are estimated in a mixed population, such that the summation of the infection profile gives the effective reproduction number.

In reality, it takes two to three days to receive a positive test result, with Lateral Flow (LFD) tests remaining positive for about five days and Polymerase Chain Reaction (PCR) tests for a longer duration. Furthermore, testing may not be uniformly distributed across the population, and the viral load may vary within the host. Therefore, we make the following assumption in this work when applying the method in the equation.

**Assumption 1** *The proposed method in* Eq (9) *assumes that the probability of a test giving a correct result is constant and uniform, and does not change with the viral load in the host. Furthermore, the relative difference in* R(α) *for any α is constant over the epidemic window we consider, and thus acts as a scaling factor* F(α) , *as defined in* Eq (6).

### 5.2 Estimating the effective reproduction number

We utilize Bayesian inference techniques to estimate the effective reproduction number. One critical information to leverage Bayesian inference to estimate the effective reproduction number is the normalized infection profile [19, 33, 41–43], which is usually referred to as the serial interval distribution [55]. The serial interval distribution captures the duration of time between the onset of symptoms in a primary case and a secondary case [56]. Following the infection profile and the basic reproduction number in Eqs (1)–(3), we define serial interval distributions of symptomatic and asymptomatic infections as

$$\begin{array}{c} \underline{w}=\underline{v}/\underline{R}=\left[{\underline{v}}_{1}/\underline{R},{\underline{v}}_{2}/\underline{R},\cdots ,{\underline{v}}_{n}/\underline{R}\right], \end{array}$$ (10)

$$\begin{array}{c} \bar{w}=\bar{v}/\bar{R}=\left[{\bar{v}}_{1}/\bar{R},{\bar{v}}_{2}/\bar{R},\cdots ,{\bar{v}}_{m}/\bar{R}\right], \end{array}$$ (11)

respectively.

Following Eqs (7) and (8), we define the modified serial interval distribution for a spreading process with both symptomatic and asymptomatic infections. This distribution, under isolation rates $\underline{\alpha }$ and $\bar{\alpha }$, and with a symptomatic infection ratio of *θ*, is denoted as *w*(*α*), where

$$\begin{array}{c} w_{i}\left(\alpha \right)=\left(\theta {\underline{v}}_{i}{\left(1-\underline{\alpha }\right)}^{i}+\left(1-\theta \right){\bar{v}}_{i}{\left(1-\bar{\alpha }\right)}^{i}\right)/R\left(\alpha \right). \end{array}$$ (12)

Recall that R(α) is defined in Eq (9), and *i* ∈ {1, 2, …, *n*} (considering *m* = *n*). Hence, based on the implemented isolation rates and the predefined infection profiles in Eqs (1) and (2), we can derive the modified infection profile in Eq (8), and subsequently obtain the modified serial interval distribution in Eq (12). Based on this information, we can apply existing methods to estimate the effective reproduction number, as outlined in Section 3.2 and Section 2D in S1 Appendix. Additionally, since the serial interval distribution is the normalized infection profile, we refer readers to Section 2C in S1 Appendix for details on how to compute the scaling factor F(α) using the serial interval distribution.

### 5.3 Reverse engineering the effective reproduction number

We propose a method to reverse engineer the effective reproduction number, assuming an alternative isolation strategy strength was implemented. This method also considers scenarios where no isolation strategy was applied, with all other spreading conditions remaining the same, indicating that the change in the strength of the intervention strategy does not further impact students’ behavior, viral loads, or other factors. Additionally, we assume a fixed total population and no loss of immunity for recovered cases. The method builds upon Eq (6), where we define the scaling factor F(α) to capture the relationship between the basic reproduction number of an outbreak with and without the implemented isolation rate *α*.

Consider the basic reproduction number of an epidemic outbreak without any isolation strategy, denoted by R. We approximate the effective reproduction number as

$${\hat{R}}_{t}=R\cdot \hat{S}\left(t\right)/N,$$

where $\hat{S}\left(t\right)>0$ is the susceptible population at time *t* > 0, and *N* > 0 is the total fixed population. Consider the same outbreak under two different isolation strategies, each with a different isolation rate, denoted by *α*_*i*_ > 0, for *i* ∈ {1, 2}. We define the basic reproduction number for these outbreaks as $R(\alpha_{i})$, for *i* ∈ {1, 2}, where $R(\alpha_{i})$ is derived from Eq (9) based on R and the corresponding infection profile. The effective reproduction numbers for the two outbreaks are given by $R_{t}\left(\alpha_{i}\right)=R\left(\alpha_{i}\right)\cdot S_{i}\left(t\right)/N$, where *S*_*i*_(*t*)>0 represents the susceptible population of the outbreak corresponding to the isolation rate *α*_*i*_, for each *t* > 0.

Consider a real-world outbreak where we can estimate the effective reproduction number, $R_{t}\left(\alpha_{1}\right)$, based on the spread under the implemented isolation rate *α*_1_. We introduce a reverse engineering method to obtain: 1) the effective reproduction number, ${\hat{R}}_{t}$, for the hypothetical spreading scenario where no isolation strategy was implemented, and 2) the effective reproduction number, $R_{t}\left(\alpha_{2}\right)$, for the hypothetical spreading scenario where an alternative isolation rate, *α*_2_, was implemented.

Using the mechanism from Eq (6), which quantifies the impact of isolation rates on the basic reproduction number, we can compute the corresponding scaling factors $F(\alpha_{i})$ for different isolation rates *α*_*i*_, given the basic reproduction number R of the same outbreak:

$$F\left(\alpha_{i}\right)=\frac{R(\alpha_{i})}{R}=\frac{R_{t}\left(\alpha_{i}\right)N/S_{i}\left(t\right)}{{\hat{R}}_{t}N/\hat{S}\left(t\right)},i\in \left\{1,2\right\}.$$

Therefore, we first obtain that

$$\begin{array}{c} {\hat{R}}_{t}=\frac{R_{t}\left(\alpha_{1}\right)\hat{S}\left(t\right)}{F\left(\alpha_{1}\right)S_{1}\left(t\right)}. \end{array}$$ (13)

Further, given that the effective reproduction number ${\hat{R}}_{t}$ and the susceptible population $\hat{S}\left(t\right)$ for the hypothetical spreading scenarios of the same outbreak without any testing-for-isolation strategies should remain the same, we conclude that

$$\begin{array}{c} R_{t}\left(\alpha_{2}\right)=\frac{R_{t}\left(\alpha_{1}\right)F\left(\alpha_{2}\right)S_{2}\left(t\right)}{F\left(\alpha_{1}\right)S_{1}\left(t\right)}. \end{array}$$ (14)

Eq (13) introduces a reverse engineering method to calculate the effective reproduction number, ${\hat{R}}_{t}$, for the hypothetical scenario with zero isolation rate, based on a real-world spreading process that occurred under the implemented isolation rate *α*_1_. Similarly, Eq (14) presents a method to leverage the estimated effective reproduction number from the real-world spread, $R_{t}\left(\alpha_{1}\right)$, under isolation rate *α*_1_, to compute the effective reproduction number $R_{t}\left(\alpha_{2}\right)$ for the hypothetical spreading scenario with an alternative isolation rate *α*_2_. Both approaches build upon the method and Assumption 1 that quantify the impact of isolation rates on the basic reproduction number, given in Section 5.1. In addition, both methods assume that the change in the isolation rate will not affect population behavior, viral loads, or other factors.

Eq (13) indicates that it is critical to consider two factors to reverse engineer the effective reproduction number: 1) The scaling factor $F(\alpha_{1})$ computed from the basic reproduction number of the spread and 2) the ratio of the susceptible populations $\frac{\hat{S}\left(t\right)}{S_{1}\left(t\right)}$ between the hypothetical and real-world spread. The scaling factor F will be lower if we have a higher isolation rate, and vice versa. Hence, it is natural to think that without the higher implemented isolation rate, the outbreak could be worse. Meanwhile, a higher ratio between the susceptible population $\frac{\hat{S}\left(t\right)}{S_{1}\left(t\right)}$ will result in higher scaling of ${\hat{R}}_{t}$ of the hypothetical spreading scenario without the isolation. In addition, Eq (14) shows that when reverse engineering the effective reproduction number for the hypothetical scenario with an alternative isolation rate *α*_2_, it is essential to consider the ratio of the scaling factors $\frac{F(\alpha_{2})}{F(\alpha_{1})}$, rather than just $F(\alpha_{1})$. For example, if *α*_2_ > *α*_1_, the ratio will be less than one, indicating a smaller reverse engineered effective production number $R_{t}\left(\alpha_{2}\right)$, thus a less severe hypothetical outbreak with the alternative isolation rate *α*_2_. Conversely, if *α*_2_ < *α*_1_, the ratio will be greater than one, indicating a more severe outbreak. Therefore, the hypothetical outbreak would have been less severe if a higher isolation rate, *α*_2_, had been implemented.

Eqs (13) and (14) require the estimation of the susceptible population in both the real-world spread (*S*_1_(*t*)) and the hypothetical spreading scenario ($\hat{S}\left(t\right)$ and *S*_2_(*t*)), which we construct using the method in Section 3.3. We briefly discuss how to obtain the susceptible population for both scenarios. In a real-world outbreak, we can monitor the spread and estimate the current susceptible population, *S*_1_(*t*), at each time step by subtracting the cumulative number of infected cases from the total population. The cumulative number of infected cases can be estimated through new daily infected cases based on the testing-for-isolation process, under the following assumptions: (1) there are no delays, and testing is uniformly distributed; (2) the testing results are accurate; (3) there is no loss of immunity among previously infected individuals during the epidemic window of study; and (4) we need complete ascertainment of cases. Even when testing delays exist, we can use the delay distributions to adjust the confirmed cases and estimate the actual number of infected cases (see Section 2D in S1 Appendix). Thus, to obtain the susceptible population, we assume that the testing-for-isolation strategy allows for accurate tracking of infected cases, as was implemented at UIUC with a high testing rate during the COVID-19 pandemic.

When reverse engineering the effective reproduction number for the hypothetical spread in a simulation environment, we can similarly calculate the susceptible population of the hypothetical outbreak, $\hat{S}\left(t\right)$ or *S*_2_(*t*). When the simulation environment generates infected cases based on the generation time interval and the effective reproduction number (Section 2B in S1 Appendix), we can record the daily new infected cases. By subtracting these infected cases from the total population, we can obtain the current susceptible population. There may be more accurate ways to estimate the susceptible population even in the presence of delays and noisy infection data. However, since estimating the susceptible population is not the primary focus of our work and represents an entire field of research in itself, we only briefly comment on potential methods for estimating the susceptible population from daily case reports. More analyses on reverse engineering the effective reproduction number for counterfactual analysis of the spread on the UIUC and Purdue campuses can be found in Section 2E in S1 Appendix.

**Algorithm 1** Reverse Engineering the ${\hat{R}}_{t}$ in a Hypothetical Outbreak

1: **Input:**

2: Time-series infection data, fixed implemented testing-for-isolation rate *α*, infection profile *v*, the total fixed population *N*, epidemic window of interest *τ*

3: **Preprocessing:**

4: Estimate time-series susceptible population data (Section 5.3 and Section 2E in S1 Appendix)

5: Scale the infection profile *v*(*α*) and calculate the serial interval distribution *w*(*α*) (Sections 5.1 and 5.2)

6: Compute the scaling factor F(α) (Section 5.1)

7: Estimate the effective reproduction number $R_{t}\left(\alpha \right)$ by leveraging infection data (Section 5.2 and Section 2D in S1 Appendix)

8: **Initialization of the hypothetical Outbreak:**

9: Set the initial infection counts for the hypothetical spreading scenario; Subtracting the cumulative infection counts from *N* to obtain the current susceptible population

10: Compute the reverse engineered effective reproduction number ${\hat{R}}_{1}$ (Section 5.3)

11: Simulate the hypothetical spread into the future for a time length *τ* (Section 3.3 and Section 2B in S1 Appendix)

12: **for** each subsequent time step *t* = 2, 3, … **do**

13:  **Step 1: Data Collection and Updates**

14:  Record time-series infected cases and compute existing susceptible population at *t* (Section 5.3)

15:  Update the scaling factor F(α) at *t* (Section 5.3)

16:  **Step 2: Compute Reverse Engineered Effective Reproduction Number**

17:  Update the reverse engineered effective reproduction number ${\hat{R}}_{t}$ using data from the hypothetical spread and the preprocessed data at *t* (Section 5.3)

18:  **Step 3: Simulate Hypothetical Spread**

19:  Generate new infected cases for the hypothetical spread into the future for a time length *τ*, marked as time step *t* + 1, using ${\hat{R}}_{t}$ (Section 3.3 and Section 2B in S1 Appendix)

20:  **if** termination condition is reached (e.g., epidemic ends, or predefined simulation criteria are met) **then**

21:   Terminate the simulation

22:  **end if**

23: **end for**

24: **End of Algorithm**

At the end of this section, we briefly formulate Algorithm 1 to present the method for reverse engineering the effective reproduction number while generating a hypothetical outbreak based on that reproduction number. The time-series infection data in the algorithm can come from a real-world spread or from a simulated outbreak that captures the real-world spread. In this work, we leverage the reconstructed real-world spread to generate the time-series infection data (solid lines in Figs 3 and 4) and the susceptible population data, while using the estimated effective reproduction number from the real-world outbreak to reverse engineer the effective reproduction number of the hypothetical spread. We apply the same spread-generation mechanism for both the real-world and the hypothetical outbreaks to better illustrate the reverse engineering method. Additionally, as mentioned in the discussion on obtaining the susceptible population, using the simulated spread facilitates computing the susceptible population. This approach avoids the need to implement another algorithm to estimate the susceptible population from real-world infection data.

### 5.4 A closed-loop feedback epidemic control algorithm

We first introduce the necessary information for implementing the closed-loop feedback control algorithm. For an epidemic spread, with any implemented testing-for-isolation strategies, we can obtain spreading data. Illustrated in Fig 1, under the condition that we know 1) the infection profiles (*v*) of the virus through the public health or the contact tracing data, 2) isolation rates ($\underline{\alpha }$ and $\bar{\alpha }$) implemented by the authorities, and 3) the ratio of symptomatic (*θ*) and asymptomatic cases (1 − *θ*) from collected data, we can estimate the effective reproduction number $R_{t}\left(\alpha \right)$ under the implemented overall isolation rate $\alpha =\theta \underline{\alpha }+\left(1-\theta \right)\bar{\alpha }$ at any given time window. If the estimated effective reproduction number $R_{t}\left(\alpha \right)$ is not equal to the expected value $R_{t}^{*}$, we need to update the isolation rate *α* (including both $\underline{\alpha }$ and $\bar{\alpha }$) for the next period. We integrate the methods developed in Eqs (6) and (9) to propose a novel control algorithm to adjust the isolation rate based on the estimated effective reproduction number.

Without loss of generality, we first introduce the algorithm without distinguishing between symptomatic and asymptomatic infections. Therefore, the overall isolation rate satisfies $\alpha =\bar{\alpha }=\underline{\alpha }$. Although the estimated effective reproduction number $R_{t}\left(\alpha \right)$ is continuous, in reality, we can estimate only a finite number of the effective reproduction number to capture the spread at certain time steps. Hence, we use $R_{k}\left(\alpha_{k}\right)$ to represent the estimated effective reproduction number under the isolation rate *α*_*k*_ at the *k*^*th*^ step, $k\in N_{>0}$. Based on the estimated effective reproduction number $R_{k}\left(\alpha_{k}\right)$ at step *k*, we propose the following algorithm to update the isolation rate *α*_*k*+1_ at the (*k* + 1)^*th*^ step.

We first compute the modified infection profile *v*(*α*_*k*_), using the estimated effective reproduction number $R_{k}\left(\alpha_{k}\right)$, under the isolation rate *α*_*k*_. Specifically, $v_{i}\left(\alpha_{k}\right)=w_{i}\left(\alpha_{k}\right)R_{k}\left(\alpha_{k}\right)$, for *i* ∈ {1, 2, …, *n*}, where *w*_*i*_(*α*_*k*_) is defined in Eq (12). If the effective reproduction number $R_{k}\left(\alpha_{k}\right)$ under the isolation rate *α*_*k*_ is not equal to the target effective reproduction number $R_{t}^{*}$ at the *k*^*th*^ step, given by $R_{k}\left(\alpha_{k}\right)\neq R_{t}^{*}$, we will update the isolation rate *α*_*k*+1_ for the (*k* + 1)^*th*^ step. The effective reproduction number, estimated through the method in Section 3.2, satisfies:

$$R_{k}\left(\alpha_{k}\right)=\sum_{i=1}^{n}w_{i}\left(\alpha_{k}\right)R_{k}\left(\alpha_{k}\right).$$

According to the definition of *w*_*i*_(*α*_*k*_) in Eq (12), i.e., the serial interval distribution under the isolation rate *α*_*k*_, we have that $\sum_{i=1}^{n}w_{i}\left(\alpha_{k}\right)=1$. Therefore, we can define the infection profile $v^{\prime }\left(\alpha_{k}\right)\in R^{n}$ that corresponds to the estimated effective reproduction number, where the *i*^*th*^ entry of the vector *v*′(*α*_*k*_) is given by

$$v_{i}^{\prime }\left(\alpha_{k}\right)=w_{i}\left(\alpha_{k}\right)R_{k}\left(\alpha_{k}\right),i\in \left\{1,2,\ldots ,n\right\}.$$

Then, we have that $\sum_{i=1}^{n}v_{i}^{\prime }\left(\alpha_{k}\right)=R_{k}\left(\alpha_{k}\right)$. According to Section 5.1, where the scaling mechanism also applies to the infection profiles of the effective reproduction number, we establish the following feedback control algorithm to update the isolation rate *α*_*k*+1_ at the next step in order to control the effective reproduction number at $R_{t}^{*}\in \left(0,1\right]$:

$$\begin{array}{c} \frac{1}{F(\alpha_{k})}\sum_{i=1}^{n}v_{i}^{\prime }\left(\alpha_{k}\right){\left(1-\alpha_{k+1}\right)}^{i}=\frac{R_{k}(\alpha_{k})}{F(\alpha_{k})}\sum_{i=1}^{n}w_{i}\left(\alpha_{k}\right){\left(1-\alpha_{k+1}\right)}^{i}=R_{t}^{*}. \end{array}$$ (15)

In Eq (15), $R_{k}\left(\alpha_{k}\right)$ is the effective reproduction number at step *k*^*th*^, under the implemented isolation rate *α*_*k*_. The only unknown in Eq (15) is the isolation rate to be updated, *α*_*k*+1_. Therefore, by solving Eq (15), we compute the updated isolation rate at the next time step directly with the feedback information from the effective reproduction number $R_{k}\left(\alpha_{k}\right)$ and the modified serial interval distribution $w\left(\alpha_{k}\right)\in R_{\geq 0}^{n}$ under the previously implemented isolation rate *α*_*k*_, making it a closed-loop feedback control policy. More details can be found in Section 2G in S1 Appendix.

We generate Algorithm 2 for the proposed closed-loop feedback control framework for the pandemic mitigation problem. In the algorithm, we use ‘period’ to represent a fixed time interval such as a week, month, or season. In addition, we can replace the isolation rate with other control intervention strategies, provided we can quantify the impact of the intervention on the infection profile.

**Algorithm 2** Closed-loop Feedback Control Framework for Epidemics

1: **Initialize:**

2: Compute the initial infection profile *v* from spreading data or obtain from public health (Section 3.1 and Section 2A in S1 Appendix)

3: Generate the initial serial interval distribution *w* (Section 5.2 and Section 2A in S1 Appendix)

4: **Input:** Target effective reproduction number $R_{t}^{*}$, initial serial interval distribution *w*

5: **for** each period *k* = 1, 2, … **do**

6:  **if** period *k* = 1 **then**

7:   Implement the daily isolation rate *α*_1_ based on the initial pandemic evaluation

8:   Compute the modified serial interval distribution *w*(*α*_1_) for *k* = 1 based on the daily isolation rate *α*_1_ (Section 5.2 and Section 2E in S1 Appendix)

9:  **else**

10:   Estimate the effective reproduction number $R_{k}\left(\alpha_{k}\right)$ at the end of period *k* using infection data collected (Section 5.2 and Section 2D in S1 Appendix)

11:   **if** $R_{k}=R_{t}^{*}$ **then**

12:    Maintain the same daily isolation rate: *α*_*k*+1_ = *α*_*k*_

13:   **else**

14:    Compute a new daily isolation rate *α*_*k*+1_ (Eq (15))

15:   **end if**

16:   Update the modified serial interval distribution as *w*(*α*_*k*+1_) (Eqs (4), (7) and (12))

17:  **end if**

18:  Proceed to the next period *k* + 1 by updating the isolation rate as *α*_*k*+1_

19: **end for**

20: **End of Algorithm**

## Supporting information

S1 Appendix Supporting information for a framework for counterfactual analysis, strategy evaluation, and control of epidemics using reproduction number estimates. (PDF)
