# Supplementary material for: A framework for counterfactual analysis, strategy evaluation, and control of epidemics using reproduction number estimates
Source: PLoS Comput Biol. 2024 Nov 20;20(11):e1012569. doi: 10.1371/journal.pcbi.1012569 (PMC11616887; doi:10.1371/journal.pcbi.1012569)
Supplement: S1 Appendix — (PDF) [file pcbi.1012569.s001.pdf]

S1 Appendix - Supporting Information for A Framework for Counterfactual Analysis, Strategy Evaluation, and Control of Epidemics Using Reproduction Number Estimates

Baike She[1], Rebecca Lee Smith[2], Ian Pytlarz[3], Shreyas Sundaram[4], Philip E. Paré[4*]

1 School of Electrical and Computer Engineering, Georgia Institute of Technology, Atlanta, Georgia, United States of America
2 Department of Pathobiology, University of Illinois Urbana-Champaign, Champaign, Illinois, United States of America
3 Institutional Data Analytics + Assessment, Purdue University, West Lafayette, Indiana, United States of America
4 Elmore Family School of Electrical and Computer Engineering, Purdue University, West Lafayette, Indiana, United States of America

* shebaike@gmail.com; philpare@purdue.edu

Summary

We present a general overview of the framework for counterfactual analysis, strategy evaluation, and feedback control of epidemics framework in S1 Fig in SI. In any testing-based epidemic spreading process, we can obtain confirmed positive cases and utilize these confirmed cases to estimate the reproduction number of the spreading process [1–3]. 1) We introduce a mechanism to quantify the impact of the testing-for-isolation intervention strategy on the basic reproduction number. Building on this mechanism, 2) we propose a method to reverse engineer the effective reproduction number under different strengths of the intervention strategy. In addition, based on the method that quantifies the impact of the testing-for-isolation strategy on the basic reproduction number, 3) we propose a closed-loop control algorithm that uses the effective reproduction number both as feedback to indicate the severity of the spread and as the control goal to guide adjustments in the intensity of the intervention. This Supporting Information provides a detailed introduction to the entire framework, including the real-world data utilized, the methodologies proposed, sensitivity analysis of the estimation of the effective reproduction number and reconstruction of the spreading process, and the goals associated with updating these strategies.

In particular, we validate the framework through leveraging COVID-19 data from the University of Illinois Urbana-Champaign (UIUC) and Purdue University (Purdue), where the intervention is the testing-for-isolation strategy. During the COVID-19 pandemic, in order to safely operate university campuses, UIUC and Purdue implemented testing-for-isolation strategies, testing a proportion of the total population on campus, including students, faculty, and staff, both universities isolated confirmed positive cases. The isolation process has not strictly forced. Confirmed positive cases were encouraged to isolate themselves from the population, aiming to keep the infected population below acceptable thresholds and prevent potential large-scale outbreaks. Both universities successfully maintained the infected population under their determined acceptable level. Inspired by these successful implementations, we validate our framework that incorporates the three proposed methods to further improve the

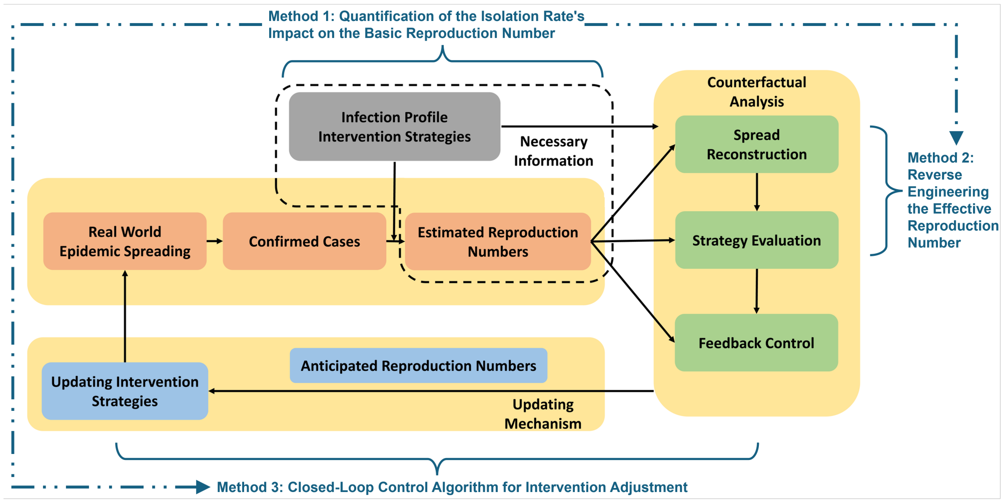

Fig S1. A framework for counterfactual analysis, strategy evaluation, and feedback control of epidemics using reproduction number estimates. The framework consists of three pieces and three methods. The first piece is to leverage real-world spreading data to estimate the effective reproduction number, where we propose a method to quantify the impact of the isolation rate on the basic reproduction number. The second step involves performing counterfactual analysis by introducing a method to reverse engineer the effective reproduction number, enabling simulation of hypothetical spreading scenarios without the implemented intervention strategy or with an alternative strength of intervention. The third component introduces a closed-loop control algorithm that uses the effective reproduction number as feedback to adjust the isolation rate, which in turn influences the effective reproduction number to manage the spread.

existing intervention strategies for spread reconstruction, strategy evaluation, and pandemic mitigation.

To summarize, in this Supporting Information, we will explain the following in detail:

- What type of data from UIUC and Purdue do we leverage to analyze the effectiveness of the testing-for-isolation strategies implemented by both universities?
- How do we quantify the impact of the intervention strategy (testing-for-isolation in this work) on the infection profile, the basic reproduction number, and the overall spreading process?
- What methodology do we propose for reverse engineering the effective reproduction number?
- How do we utilize the reverse engineered effective reproduction number to conduct counterfactual analysis of the outbreak?
- How can the closed-loop feedback control algorithm serve as a foundation to facilitate policy-making for pandemic control?

SI-1. Intervention Strategies and Data from UIUC and Purdue

SI-1-A. Background

First, we present the data sets. All the real-world data from UIUC and Purdue were collected by the SHIELD team at the University of Illinois Urbana-Champaign and Institutional Data Analytics + Assessment at Purdue University, respectively. During the COVID-19 pandemic, the SHIELD team at UIUC relied on guidance from reputable sources such as the Centers for Disease Control and Prevention, the Illinois Department of Public Health, and the Champaign Urbana Public Health District. They actively monitored the pandemic on and around campus and adapted their decisions in response to the evolving understanding of COVID-19. The IDA+A team at Purdue leveraged COVID-19 data specifically from the Purdue campus to perform statistical analysis and construct sampling methodologies. Their findings and reports were provided to campus leaders and decision-makers to assist in evaluating the spread of the virus on campus and the effectiveness of intervention strategies implemented at Purdue. Both teams adopted testing-for-isolation strategies to assess the severity of the pandemic and made necessary adjustments to their plans.

At the early stage of the COVID-19 pandemic, when the availability of an efficient vaccine was absent, significant challenges arose in mitigating the spread of the virus. Smart communities with dense populations, such as technology companies and research laboratories, began adopting remote working practices. In order to ensure the safe operation of densely populated areas, large universities like the University of Illinois Urbana-Champaign and Purdue University implemented pre-arrival testing, regular screening, and voluntary testing, as well as case isolation, contact tracing, and quarantine to mitigate the spread of infection. These strategies involved testing a proportion of the total campus population, enabling the identification of infected cases. Subsequently, infected and high-risk individuals were encouraged to isolate themselves within their own residences or be quarantined in designated quarantine centers. As a result, the severity of the epidemic on these campuses decreased since a portion of the infected population was no longer actively transmitting the virus. Consequently, Purdue and UIUC effectively maintained the infected population at levels that allowed for safe operation throughout the semesters, despite experiencing periodic spikes. This success highlights the potential of testing-for-isolation strategies in mitigating future pandemics.

In order to better support the testing-for-isolation strategy, different universities implemented unique methodologies. For example, the team at UIUC proposed an agent-based model to capture the spreading on campus [4], while researchers at UCSD and Harvard studied the impact of testing using a network model [5]. Emory University developed a compartmental model to study SARS-CoV-2 spreading among disparate populations of students, faculty, and staff [6]. In addition, the researchers in [7] proposed a group of models to simulate the spread over campuses after reopening. To determine whether in-person instruction could safely continue during the pandemic and evaluate the necessity of various interventions, [8] utilized a stochastic agent-based model to study the spread over campuses. A customized susceptible, exposed, infected, and recovered compartmental model was presented in [9] to describe the control of asymptomatic spread of COVID-19 infections on the Boston University campus. Furthermore, an agent-based model on a network aimed at capturing unique features of COVID-19 spread through small residential colleges was proposed in [10]. Moreover, [11] used mathematical models to evaluate the impact of class sizes on the reproduction number to suppress the spread of the virus on campus, specifically in classrooms.

SI-1-B. Testing-For-Isolation at UIUC and Purdue

We first introduce the aggregated data from UIUC. The University of Illinois Urbana-Champaign conducted campus-wide surveillance testing twice a week during Fall 2020 and three times a week during Spring 2021, illustrated by S2 Fig in SI. When testing everyone twice a week during Fall 2020 at UIUC, the tests were not sampled on the same day due to the daily laboratory capacity on the UIUC campus being up to 10,000 tests. For simplicity, testing-for-isolation strategies can be considered as a policy implemented on a weekly basis and distributed evenly throughout the week. An intervention that can mitigate a potential outbreak is an isolation intervention after testing positive. Ideally, the daily isolated population should equal the daily confirmed cases. However, because isolation is not mandatory, we use the isolation rate instead of the testing rate to describe the effectiveness of the testing-for-isolation strategy.

In order to estimate the effective reproduction number, it is important to distinguish between infected cases and confirmed cases [1, 3]. An infected case means that an individual is already infected by the virus, but they may not be contagious yet due to the existence of an incubation period. Therefore, an infected case is not equivalent to an infectious case. Meanwhile, a confirmed case refers to a case that has been reported as infected, but there may be delays between the time of infection and confirmation due to testing and reporting delays. All the data we obtained from UIUC and Purdue are considered confirmed cases rather than infected cases.

After summarizing the testing-for-isolation strategy implemented by UIUC, we further introduce the data collected by UIUC. The surveillance testing-for-isolation strategy was supported through spatial analyses in order to target high-risk regions such as fraternity houses. The daily confirmed cases are presented in S3 Fig in SI. In the Methodology Section, we provide a more detailed explanation of the distinction between confirmed cases and infected cases. From S3 Fig in SI, we observe multiple spikes during Fall 2020 and Spring 2021 on the UIUC campus. We highlight two significant spikes. The first spike occurred around the middle of August 2020 when UIUC implemented an entry-screening to identify the infected population returning to campus. The second spike occurred around the middle of October 2020, which we attribute to gathering events associated with the return of the college football season in Fall 2020. In addition to the entry-screening, UIUC managed to maintain a consistently low level of daily confirmed cases with mild fluctuations under the high surveillance testing frequency.

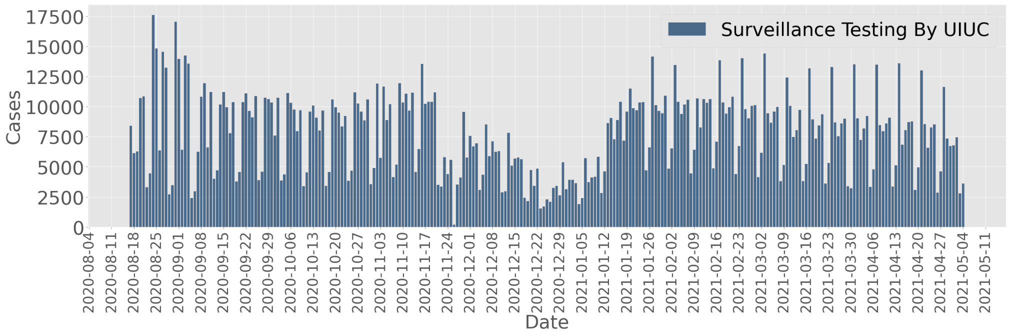

Fig S2. Daily surveillance testing at UIUC during Fall 2020 and Spring 2021 implemented by the SHIELD team at UIUC. The data shows weekly effect, with more testing conducted on Mondays and less testing on weekends.

Distinct from UIUC, which relied on a surveillance testing-for-isolation strategy, Purdue University implemented a different testing-for-isolation strategy. Purdue allocated testing resources into two categories. The first category included symptomatic cases and cases identified through contact-tracing analysis, requiring these individuals

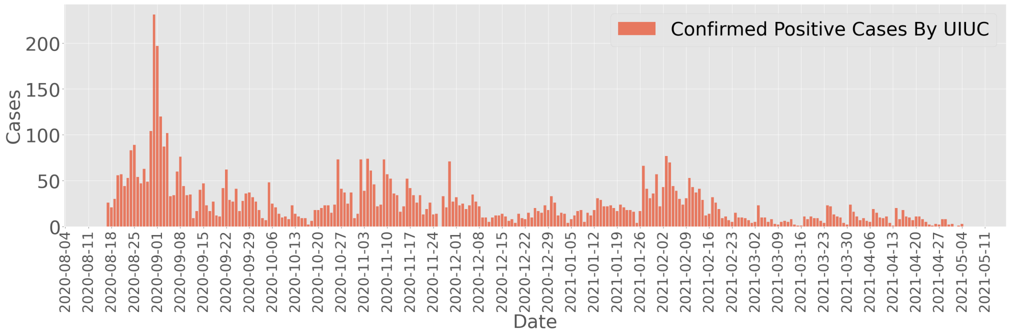

Fig S3. Daily confirmed positive cases at UIUC during Fall 2020 and Spring 2021 captured by the SHIELD team at UIUC. Obvious spikes can be observed at the beginning of Fall 2020, the middle of Fall 2020, and the beginning of Spring 2021. These spikes correspond to the entry-screening at the beginning of Fall 2020, the return of the Big Ten football Season of Fall 2020, and entry-screening at the beginning of Spring 2021.

to undergo testing. In this work, we refer to this type of testing-for-isolation strategy as voluntary testing-for-isolation. The allocation of voluntary testing resources related to this category is illustrated in S4 Fig in SI. However, COVID-19 also involves asymptomatic infections [12]. To capture asymptomatic cases, Purdue designated approximately 5000 tests per week (approximately 8% to 12% of the total campus population) for surveillance testing-for-isolation during Fall 2020. Additionally, Purdue increased the surveillance testing rate during Spring 2021 to target more active spreading areas, such as fraternity houses. As shown in S5 Fig in SI, there was an increase in the amount of surveillance testing resources used in Spring 2021.

In detail, Purdue's strategy involved randomly sampling and testing a proportion of the campus population. We refer to this type of testing-for-isolation strategy as surveillance testing, and Purdue's allocation of testing resources is shown in S5 Fig in SI. The absence of the surveillance testing dataset from the middle of November 2020 to the beginning of January 2021, as depicted in S5 Fig in SI, reflects the fact that Purdue University sent students back home after the Thanksgiving break in Fall 2020, and students did not return to campus until the Spring 2021 semester. Although we simplify the testing-for-isolation strategy implemented by Purdue in our analysis, we still introduce more details about Purdue's testing-for-isolation strategy. For residential students, Purdue randomly sampled 8% to 12% of each residence hall floor based on their analyses. For off-campus students, Purdue utilized various contact tracing elements that the IDA+A team constructed in consultation with experts. The IDA+A team created a series of clusters in a contacting network representing the off-campus student body. Each cluster connected students via different contact tracing metrics such as dining swipes and network logs. Each student in the cluster was assigned centrality and connectedness features, which were then summed across each cluster to give each student an aggregate 'connectedness' value. Purdue then performed a weighted random sample, weighting by connectedness, from the entire off-campus student body. The amount of this sampling varied week-to-week depending on test availability.

We additionally present the confirmed cases through the surveillance testing-for-isolation strategy and the total confirmed cases in S7 Fig in SI and S8 Fig in SI during Fall 2020 and Spring 2021, respectively. From S7 Fig in SI, we conclude that the surveillance testing-for-isolation strategy successfully captured asymptomatic cases. Similar to UIUC during Fall 2020, S8 Fig in SI exhibits two significant spikes. The first spike, occurring around the middle of August, was related to the entry-screening

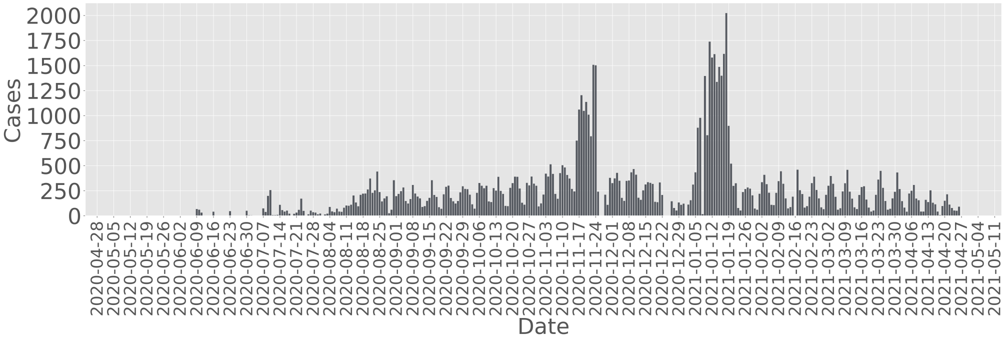

Fig S4. Daily voluntary tests at Purdue University during Fall 2020 and Spring 2021 captured by the IDA+A team at Purdue. The first main spike corresponds to the change in weather and students gathering during the college football season. The second main spike corresponds to the situation when students were voluntarily tested after returning from the Christmas break.

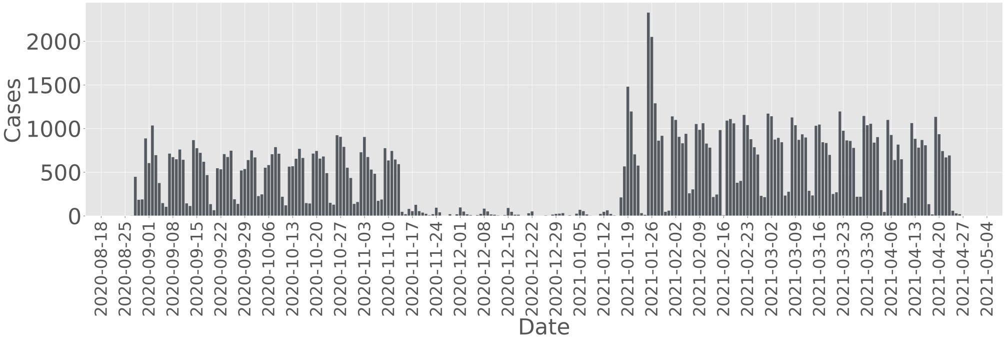

Fig S5. Daily surveillance tests at Purdue University during Fall 2020 and Spring 2021 captured by the IDA+A team at Purdue. Compared to Fall 2020, Purdue increased the surveillance testing rate during Spring 2021 to target more active spreading areas. The absence of the surveillance testing dataset, from the middle of November 2020 to the beginning of January 2021, reflects the fact that Purdue University sent students back home after the Thanksgiving break in Fall 2020, and students did not return to campus until the start of the Spring 2021 semester.

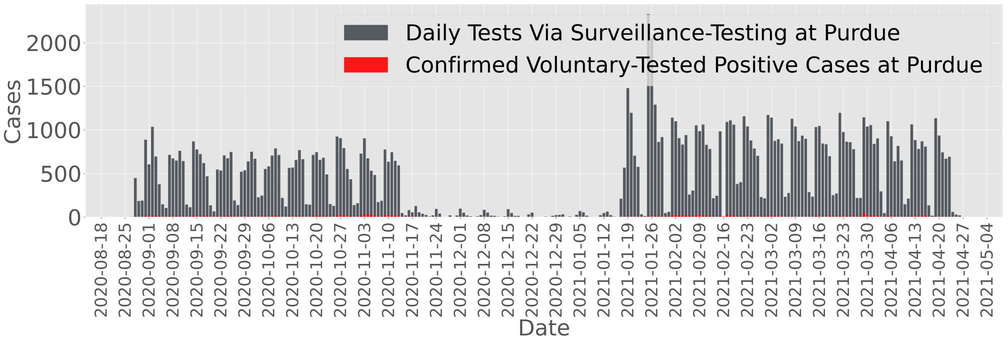

Fig S6. Daily surveillance tests and confirmed cases through surveillance testing at Purdue University during Fall 2020 and Spring 2021 captured by the IDA+A team at Purdue.

conducted at the beginning of the semester. The other spike was associated with

gathering events resulting from the return of the college football season towards the end of October.

By comparing the total confirmed cases between UIUC (S3 Fig in SI) and Purdue (S8 Fig in SI), we observe that Purdue University had a higher average daily number of confirmed cases and experienced greater fluctuations, while allocating fewer testing resources. We can intuitively conclude that higher testing rates at UIUC enabled the timely identification and isolation of infected cases, thereby reducing the spread of the virus. Consequently, compared to Purdue, UIUC had fewer confirmed cases at the cost of significantly more testing resources. Moving forward, we primarily leverage the total confirmed cases from both universities, under their respective testing-for-isolation strategies, to validate the proposed pandemic mitigation framework for counterfactual analysis, strategy evaluation, and feedback control.

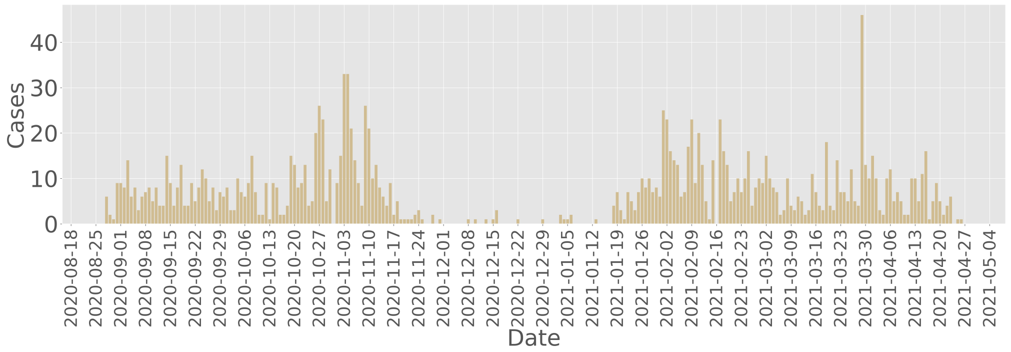

Fig S7. Daily confirmed positive cases at Purdue University during Fall 2020 and Spring 2021 captured by the surveillance testing. Although the surveillance testing kits remained unchanged weekly, the confirmed cases still reflect potential spikes.

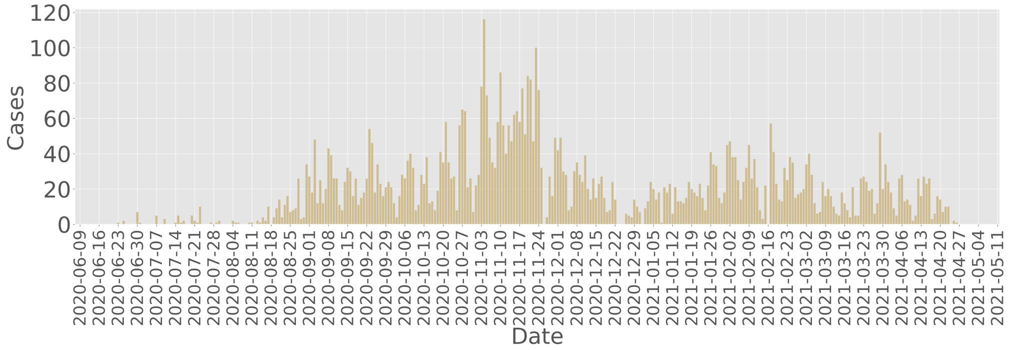

Fig S8. Total daily confirmed positive cases at Purdue University during Fall 2020 and Spring 2021. Similar to UIUC, three significant spikes can be observed. The first spike, occurring around the middle of August, was related to the entry-screening conducted at the beginning of the Fall semester. The second spike was associated with gathering events resulting from the return of the college football season towards the end of October. The third spike, occurring around the beginning of 2021, was related to the entry-screening conducted at the beginning of the Spring semester.

SI-1-C. Difference Between Isolation and Quarantine

In addition to presenting testing resources and confirmed cases from Purdue University, we further illustrate the implemented testing-for-isolation strategy at Purdue through

the provided isolation data in S9 Fig in SI and quarantine data in S10 Fig in SI. When a case is confirmed to be infected through testing, Purdue University encourages the case to isolate from others and designates it as an isolated case. The termination of the isolation period is determined by whether the case tests negative or not. Therefore, the isolation data in S9 Fig in SI can be considered as the cumulative number of infected cases with an average recovery time of one to two weeks. During the Winter break, most students were not on campus, resulting in only a few recorded isolated cases during Winter 2020.

Unlike isolated cases, where all individuals were confirmed infected, Purdue also implemented contact tracing strategies to identify and quarantine individuals who had close contact with confirmed cases, as shown in S10 Fig in SI. During the Fall 2020 semester, Purdue implemented a contact-tracing-based testing-for-isolation strategy by targeting a few closely contacted cases and encouraging them to quarantine. To enhance the effectiveness of contract-tracing, Purdue established a more complicated contact-tracing network, as described before. By constructing these contact-tracing networks, Purdue improved its contact-tracing policy during Spring 2021, resulting in more closely contacted cases being traced and subsequently quarantined. Consequently, compared to Fall 2020, we observe a significant increase in the number of daily quarantined cases, as depicted in S10 Fig in SI.

Both UIUC and Purdue distinguish between isolation and quarantine. Quarantine keeps someone who has been in close contact with someone who has COVID-19 away from others, while isolation keeps someone who is sick or has tested positive for COVID-19 away from others, even within their own home. In this work, we refer to isolation as a measure to prevent those who tested positive from further spreading the virus. Therefore, we consider the isolation rate as the intensity of our testing-for-isolation strategy. We do not consider quarantine in the proposed framework. Additionally, testing at both universities was not conducted through uniformly random sampling in reality. Both universities leveraged their spatial data to target and enhance the testing-for-isolation strategy in high-risk areas. However, for simplicity, we assume the isolation rate is implemented through uniformly random sampling across the population in our framework.

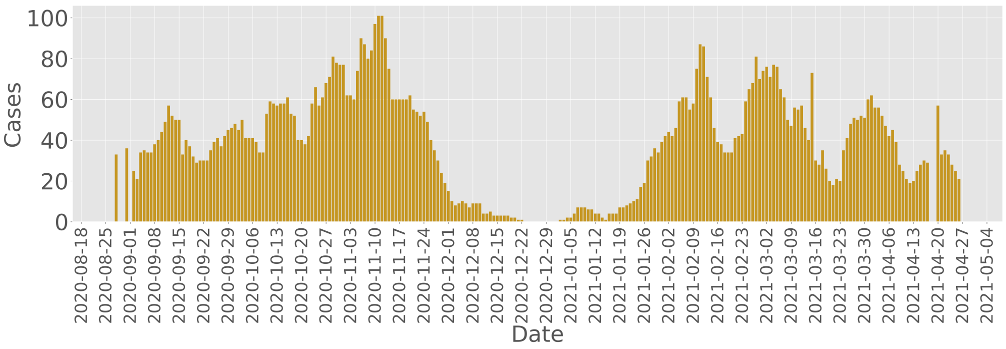

Fig S9. Daily isolated cases at Purdue University during Fall 2020 and Spring 2021. The isolation data can be considered as the cumulative number of infected cases with an average recovery time of one to two weeks. During the Winter Break, most students were not on campus, resulting in only a few recorded isolated cases during Winter 2020.

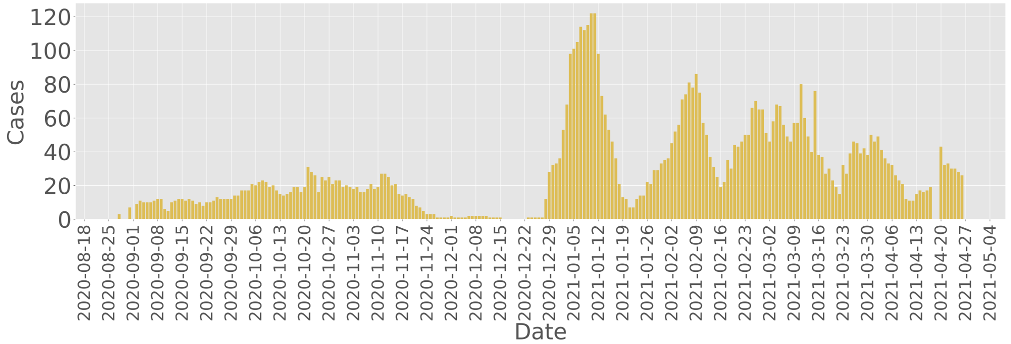

Fig S10. Daily quarantined cases at Purdue University during Fall 2020 and Spring 2021. Purdue implemented a contact-tracing-based testing-for-isolation strategy by targeting a few closely contacted cases and encouraging them to quarantine. Purdue improved its contact-tracing policy during Spring 2021 by expanding these contact-tracing networks, which resulted in more closely contacted cases being traced and subsequently quarantined.

SI-2. Methods

We introduce the core methods that we leverage and develop for the framework in S1 Fig in SI. We present the mechanism used to generate synthetic data to simulate the spreading processes. This procedure includes explaining how to estimate the effective reproduction number based on the confirmed cases under the impact of the implemented intervention strategy, specifically the testing-for-isolation strategies, as indicated within the dashed region in S1 Fig in SI.

SI-2-A. Epidemic Infection Profiles

For an epidemic spreading process such as COVID-19, it is impossible for us to compare the effectiveness of different strengths of an implemented intervention strategy under the exact spreading conditions at the exact same time in reality. This inherent characteristic of epidemics presents challenges when proposing epidemic evaluation and feedback control design. However, it is important and necessary to evaluate different intervention strategies for hypothetical spreading scenarios in order to prepare for future outbreaks. Therefore, counterfactual analysis, a method used to evaluate what might have happened in a situation, is critical. We first introduce a method that leverages the reproduction number and random processes to generate daily confirmed cases, which serves as a foundation for reconstructing the spread over the UIUC and Purdue campuses with their implemented interventions in the next section. This approach facilitates the counterfactual analysis of alternative intensities of intervention strategies in the hypothetical spreading scenarios. We further assume that nothing else changes in the hypothetical spreading scenario, meaning that the change in the strength of the intervention strategy does not further impact students' behavior, viral loads, or other factors.

SI-2-A-1. Infection Profile and the Basic Reproduction Number

The infection profile represents the average time between the onset of infections in a primary case and the onset of infections in its secondary cases. Different infectious diseases exhibit distinct spreading behaviors, leading to varying infection profiles. Typically, the profile reflects pathogen shedding, with a single peak indicating pathogen

growth followed by immune suppression or host death. This profile also indicates the effective contact rate between infectious and susceptible individuals, which can vary. Moreover, the infection profile of the same virus can differ due to changes in the spreading environment. Factors such as the age structure of the population can influence the infection profile. Additionally, symptomatic and asymptomatic infections may generate different infection profiles within the same population [13–15].

Hence, to generalize the methodology developed in this work, we denote the infection profile of symptomatic infections as follows:

$$\underline{v} = [\underline{v}_1, \underline{v}_2, \cdots, \underline{v}_n],$$

where $\underline{v} \in \mathbb{R}^n_{\geq 0}$ and n is the average number of days during which a symptomatic case is infectious. We use $\underline{v}_i \in \mathbb{R}_{\geq 0}$ to represent the average number of infected cases that a symptomatic case can generate on day i. Similarly, we define infection profile of asymptomatic cases as a vector

$$\overline{v} = [\overline{v}_1, \overline{v}_2, \cdots, \overline{v}_m],$$

where $\overline{v} \in \mathbb{R}^m_{\geq 0}$ and m is the number of the days during which an asymptomatic case is infectious. We use $\overline{v}_i \in \mathbb{R}_{\geq 0}$ to represent the average number of infected cases that an asymptomatic case can generate on day i.

The basic reproduction number can capture the average number of infected cases generated by one infected individual in a nearly fully susceptible population [16]. Hence, if the infection profiles $\overline{v}$ and $\underline{v}$ are obtained in a nearly fully susceptible population, it is naturally to bridge the gap between the infection profile and the basic reproduction number through the following equations:

$$\underline{\mathcal{R}} = \sum_{i=1}^{n} \underline{v}_i, \quad \overline{\mathcal{R}} = \sum_{i=1}^{m} \overline{v}_i, \tag{1}$$

where $\underline{\mathcal{R}}$ and $\overline{\mathcal{R}}$ are the basic reproduction numbers of the symptomatic and asymptomatic infections, respectively. Further, for an infectious disease that can generate both symptomatic and asymptomatic infections, if symptomatic cases are θ percent, the basic reproduction number of the spreading process is defined as

$$\mathcal{R} = \theta \sum_{i=1}^{n} \underline{v}_i + (1-\theta) \sum_{i=1}^{m} \overline{v}_i = \theta \underline{\mathcal{R}} + (1-\theta)\overline{\mathcal{R}}. \tag{2}$$

Specifically, in order to facilitate comparisons of the implemented intervention strategy, we utilize the same infection profile of the COVID-19 pandemic for both UIUC and Purdue, given by [17, 18]

$$v = \underline{v} = \overline{v} = [0.148, 1.0, 0.823, 0.426, 0.202, 0.078, 0.042, 0.057, 0.009]. \tag{3}$$

Eq (3) were leveraged by a research team at UIUC [4]. We illustrate the infection profile in Eq (3) by S11 Fig in SI (Left). Additionally, based on Eq (2), the basic reproduction number we leverage for COVID-19 is $\mathcal{R} = 2.785$. In epidemic mitigation and prediction, researchers not only rely on the basic reproduction number but also focus on the effective reproduction number. Unlike the basic reproduction number, the effective reproduction number captures the average number of infected cases generated by one infected case in a mixed population, including susceptible, recovered, and infected individuals, etc. If we can construct the infection profile vector in a mixed population, we can obtain the effective reproduction number by summing all entries in the vector, similar to the computation of the basic reproduction number. When the

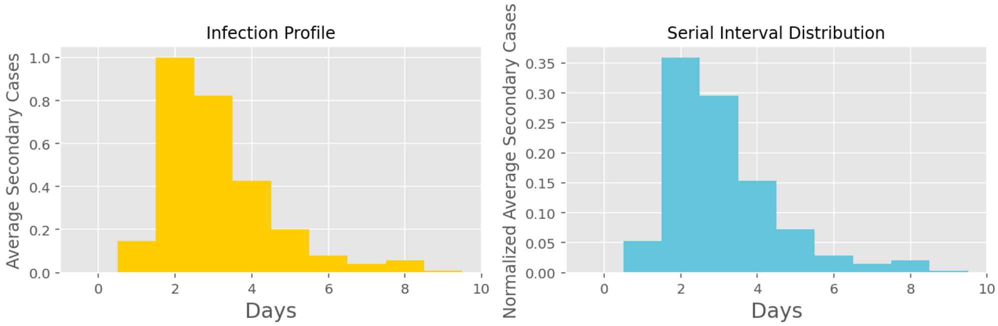

Fig S11. One COVID-19 infection profile (Left); Serial interval distribution (Right) [17, 18]. The infection profile captures the average daily infected cases caused by one primary infected case, as given by Eq (3). The serial interval distribution on the right provides the normalized infection profile.

basic reproduction number of an infectious disease spreading over a population is known, it is common to approximate the effective reproduction number $\mathcal{R}_t$ by scaling the basic reproduction number using the following equation:

$$\mathcal{R}_t = \frac{S(t)}{N}\mathcal{R}, \tag{4}$$

where N represents the total population and $S(t) \in [0, N]$ represents the susceptible population at time $t \in [0, \infty)$. Researchers proposed various methods to estimate the effective reproduction number: utilizing data on infected cases, confirmed cases, hospitalized cases, and more. In this work, we employ a popular method based on Bayesian inference to estimate the reproduction number using confirmed cases [1–3]. To do so, we first need to introduce the concept of the serial interval distribution.

SI-2-A-2. Serial Interval Distributions

A serial interval distribution represents the normalized infection profile. If an infected case can transmit the infection to another person, it defines the probability distribution of the duration between the infection of the primary case and the infection of the secondary case. Based on this definition, we can define the serial interval distributions of symptomatic and asymptomatic infections as

$$\underline{w} = \underline{v}/\underline{\mathcal{R}} = [\underline{v}_1/\underline{\mathcal{R}}, \underline{v}_2/\underline{\mathcal{R}}, \cdots, \underline{v}_n/\underline{\mathcal{R}}],$$
$$\overline{w} = \overline{v}/\overline{\mathcal{R}} = [\overline{v}_1/\overline{\mathcal{R}}, \overline{v}_2/\overline{\mathcal{R}}, \cdots, \overline{v}_m/\overline{\mathcal{R}}],$$

respectively. Note that $\underline{w} \in [0, 1]^n$ and $\overline{w} \in [0, 1]^m$. According to Eq (1) and Eq (2), the serial interval distribution of a spreading process with both symptomatic and asymptomatic infections is given by

$$\begin{aligned} w = [&(\theta\underline{v}_1 + (1-\theta)\overline{v}_1)/\mathcal{R}, (\theta\underline{v}_2 + (1-\theta)\overline{v}_2)/\mathcal{R}, \\ &\ldots, (\theta\underline{v}_m + (1-\theta)\overline{v}_m)/\mathcal{R}, \theta\underline{v}_{m+1}/\mathcal{R}, \ldots, \theta\underline{v}_n/\mathcal{R}], \end{aligned} \tag{5}$$

where $n \geq m$. Note that, $\sum_{i=1}^{n} \underline{w} = \sum_{i=1}^{m} \overline{w} = \sum_{i=1}^{n} w = 1$ by definition, since the serial interval distribution is a probability distribution. In this work, we consider

$$w = \underline{w} = \overline{w} = [0.053, 0.36, 0.29, 0.153, 0.078, 0.028, 0.015, 0.02, 0.003], \tag{6}$$

where Eq (6) is obtained by normalizing the infection profile in Eq (3). We show how to leverage the serial interval distribution to generate spreading data, which will lay a foundation for estimating the effective reproduction number.

SI-2-B. Generating Spreading Data

We introduce a mechanism for generating spreading data that matches real-world spreading processes [2, 19]. We utilize this data-generation method to generate confirmed cases that mimic the confirmed cases from UIUC and Purdue. The data generation method is based on a widely-used model, which can be found in [1, 2] and in the Python package *EpyEstim* [19]. We consider an epidemic spreading process with the serial interval distribution, given by w, which follows a Poisson process, such that the number of new generated infected cases I_t at time t is Poisson-distributed [1], with mean

$$\mathbb{E}(I_t) = \mathcal{R}_t \sum_{s=1}^{t} I_{t-s} w_s. \tag{7}$$

Further, we define $\Lambda_t = \sum_{s=1}^{t} I_{t-s} w_s$. Then, the probability distribution of k infected cases on day t [1], is denoted by

$$\mathbb{P}(I_t = k) = \frac{(\mathcal{R}_t \Lambda_t)^k e^{(-\mathcal{R}_t \Lambda_t)}}{k!}. \tag{8}$$

Therefore, we have $I_t \sim Pois(\lambda = \mathcal{R}_t \Lambda_t)$, where $Pois(\lambda)$ denotes the Poisson distribution with mean λ. Eq (8) implies that the new infected cases at time step t are determined by the serial interval distribution w, the existing daily infected cases I_t, $t \in \{t-1, t-2, \dots, 0\}$, and the effective reproduction number at time step t, i.e., $\mathcal{R}_t$. By further investigating Eq (7), the term Λ_t represents the convolution of the daily infected cases and the serial interval distribution of the corresponding disease. If $t - s$ is greater than the length of the serial interval distribution w, the average number of new daily infected cases prior to or on day $t - s$ cannot generate new infections on day t, meaning that the infected cases becoming infectious on day zero are already non-infectious on day $t - s$. Furthermore, Λ_t can only generate "scaled" new infected cases because w is a normalized infection profile. To obtain the average number of new infected cases based on Eq (7), we need to scale Λ_t by the current effective reproduction number $\mathcal{R}_t$. Hence, we obtain Eq (7) to describe the average daily number of new infected cases. A more detailed introduction and discussion on how to leverage this technique to capture new infected cases can be found in [1, 2, 19].

After introducing the mechanism to generate infected cases, we use the following example to illustrate the process of generating new infected cases using Eq (7) and Eq (8). The data generation process is built upon the Python package to generate infection data and estimate the effective reproduction number [19]. We consider an epidemic spreading process over a sufficiently large population with a fixed serial interval distribution, as given in Eq (3). Since we have a sufficiently large population, we approximate the effective reproduction number using the basic reproduction number of the spreading process, as shown in S12 Fig in SI. We consider the initial infected cases from day one to day seven, i.e., the initial condition to be $[1, 0, 1, 0, 2, 1, 3]$. By implementing the mechanism described by Eq (7) and Eq (8), we generate the infected cases using the Poisson process [15]. S13 Fig in SI shows one typical simulation of the daily infected cases. We can see from S13 Fig in SI that the trend of the infected cases matches the changes in the effective reproduction number. A higher effective reproduction number results in larger spikes in the number of infected cases, while the number of the infected cases begins to decrease when the effective reproduction number is less than one.

In reality, directly obtaining the infected cases described in S13 Fig in SI is extremely challenging. Instead, it is more common to have access to confirmed cases, which include a delay between a case being infected and being confirmed. This period is influenced by factors such as the incubation period, testing delays, and reporting delays.

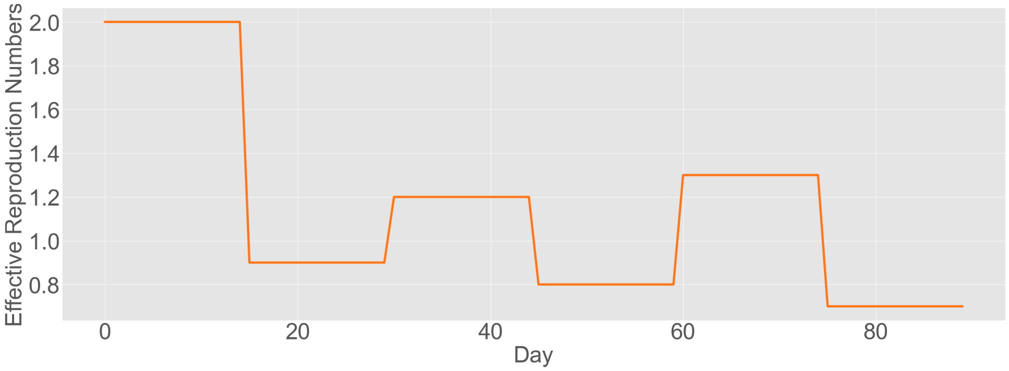

Fig S12. The simulated effective reproduction number. The effective reproduction number, whether higher or lower than one, influences the generation of a spreading process with spikes and decreases.

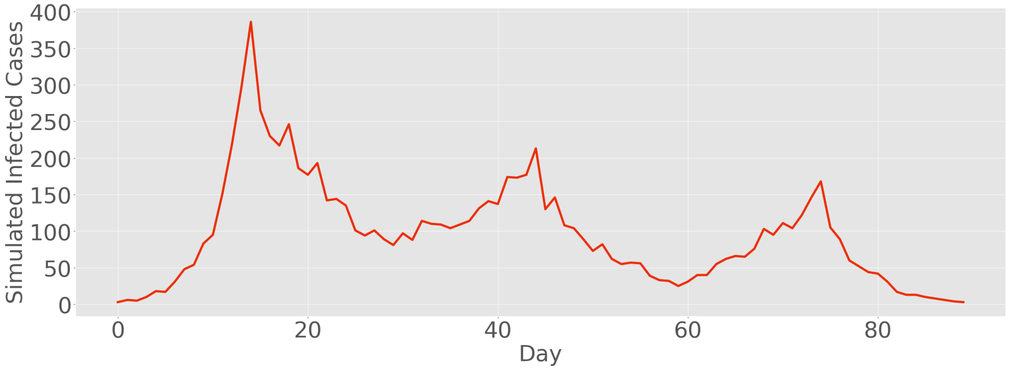

Fig S13. Simulated daily infected cases. The trend of the infected cases matches the changes in the effective reproduction number in S12 Fig in SI. A higher effective reproduction number results in larger spikes in the number of infected cases, while the number of the infected cases begins to decrease when the effective reproduction number is less than one.

For instance, all the cases we collected in S3 Fig in SI and S8 Fig in SI were confirmed cases rather than infected cases. To simulate confirmed cases in the modeling process, we need to generate confirmed cases based on the infected cases. The idea is to add factors such as the incubation period, testing delays, and reporting delays to the simulated infected cases.

The incubation period captures the time from when an infected case becomes infected to when it becomes infectious [20]. Testing and reporting delays capture the delays from becoming infectious to being reported/confirmed. In this work, we mainly consider these two delays. We consider the incubation period of the infections during Fall 2020 and 2021 follows the distribution given by [21, 22], where the incubation period of an infected case follows a discrete Gamma distribution with shape parameter 1.35 and scale parameter 3.77. Meanwhile, we leverage another discrete Gamma distribution, with shape parameter 2 and scale parameter 3.2 to capture the testing-to-confirmation delay [23]. We generate the combined delay distribution via convolution between the incubation period distribution and the testing-to-confirmation delay distribution [19]. The combined distribution is defined as the infection-to-confirmation delay distribution, denoted as Δ. The mean of the infection-to-confirmation delay distribution Δ in this work is 10.3-day [2, 19, 21]. Through the convolution between the

infection-to-confirmation delay distribution and the infected cases generated by Eq (7) and Eq (8), we obtain the synthetic data to describe confirmed cases [2], given by

$$C_t = \sum_{s=1}^{t} I_{t-s} \Delta_s, \tag{9}$$

where C_t represents confirmed cases on day t from the infected cases with incubation period and testing-to-confirmation delays.

Additionally, to better align with the weekly testing and reporting patterns implemented by UIUC and Purdue, as shown in S6 Fig in SI, we introduce noise and weekly patterns to the confirmed cases through the methodology in [2]. We utilize a sinusoidal function to generate the weekly testing and reporting pattern. By incorporating noise and weekly patterns, we can generate confirmed cases that reflect the infected data shown in S13 Fig in SI, as illustrated in S14 Fig in SI. In comparison to S13 Fig in SI, the confirmed cases in S14 Fig in SI exhibit delayed infection with weekly patterns. More detailed techniques about generating synthetic confirmed data can be found in [2, 19].

We utilize the techniques introduced in this section to generate confirmed data for both UIUC and Purdue, aiming to match the confirmed cases observed on both campuses. To achieve the data generation process, we need to estimate the effective reproduction number $\mathcal{R}_t$ through the confirmed cases, taking into account the impact of the implemented testing-for-isolation strategies. As a result, we propose a method to quantify the impact of the testing-for-isolation intervention on spreading processes.

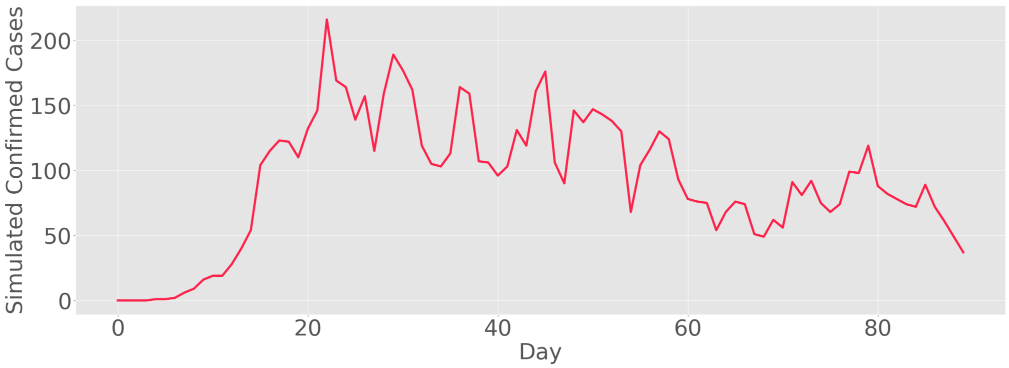

Fig S14. Simulated daily confirmed cases. Compared to S13 Fig in SI, the confirmed cases exhibit delayed infections with weekly patterns, which lays a foundation for simulating the COVID-19 spread across university campuses.

SI-2-C. The Impact of Testing-For-Isolation on Spreading Processes

We assume that the spreading processes we study in this work follow the serial interval distribution normalized from the infection profile in Eq (3). Additionally, we explore the impact of testing-for-isolation strategies on the serial interval distribution, since the testing-for-isolation strategy, i.e., the isolation rate, is the only control intervention we adjust [24]. Typically, researchers qualitatively study the impact of interventions on serial interval distributions by leveraging real-world spreading data [21]. However, one disadvantage of studying the impact of the intervention strategy at different intensity is the limited amount of data from the irreversible features of the spread. Hence, it is challenging to directly quantify the influence of all possible intervention strategies on the spread using real-world data.

To address this problem, we propose a novel method to quantify the impact of the testing-for-isolation strategy on the infection profile, and thus, the basic reproduction number and the spread. In particular, the method we propose presents a new way to compute how the isolation rate affects the infection profile and serial interval distribution and subsequently alters the spreading process. Using this method, we can reverse engineer the effective reproduction number of a real-world spread under the implemented isolation rate to compute the effective reproduction number of the hypothetical spread under alternative isolation rates. To introduce the method, we first examine the impact of the isolation rate on the infection profile and the basic reproduction number. Then, we map the influence to the serial interval distribution. This method provides valuable insights into understanding the relationship between the isolation rate and the spreading process. By quantifying the impact of the testing-for-isolation strategy on the serial interval distribution, we can better assess the effectiveness of different isolation rates in evaluating and controlling the spread of infectious diseases.

We consider implementing testing-for-isolation strategies for a mix of symptomatic and asymptomatic cases to simplify the introduction and validation. In our approach, the symptomatic and asymptomatic cases have the exact same infection profile. However, the same mechanism can be applied to symptomatic infections and asymptomatic infections with different infection profiles through Eqs (1) and (2), or even a disease with multiple infection profiles based on the host. If we uniformly randomly sample a proportion α, $\alpha \in (0, 1]$, of the population on campus to test daily, we can confirm and isolate α times the total infected population daily, assuming the ideal situation where we can isolate all confirmed cases immediately. Consequently, the isolated cases cannot infect others. Therefore, we consider the removal of an average of α of the total infected cases each day. In this context, we consider that the infection profile of a spreading process is represented by the vector $v = [v_1, v_2, \ldots, v_n]$. Then, we propose the modified infection profile under the daily isolation rate α as

$$v(\alpha) = [v_1(1-\alpha), v_2(1-\alpha)^2, \cdots, v_n(1-\alpha)^n]. \tag{10}$$

We explain Eq (10) step by step: If we have $k \in \mathbb{N}_{>0}$ infectious cases on day one, without testing-for-isolation strategies, these k infectious cases will generate an average number of kv_i cases on day i, $i \in \{1, 2, \ldots, n\}$. However, consider the same number of k infectious cases under a testing-for-isolation strategy. If we test and then isolate $k\overline{\alpha}$ cases on day one from the k cases, there will be $kv_1(1-\overline{\alpha})$ new infected cases that are generated by the $k(1-\overline{\alpha})$ cases. On day two, there will be $kv_2(1-\overline{\alpha})^2$ cases generated by $k(1-\overline{\alpha})^2$ infectious cases. Consequently, the new infected cases caused by the original k infectious cases are $kv_i(1-\overline{\alpha})^i$ on day i, $i \in \{1, 2, \ldots, n\}$. Thus, the average number of infected cases generated by a single infectious individual on day i is given by $v_i(1-\overline{\alpha})^i$, $i \in \{1, 2, \ldots, n\}$. Then, we can obtain the infection profile of the original k infectious cases as shown in Eq (10). Eq (10) provides a method to quantify the impact of the daily isolation rate on the initial infection profile. Given a certain spreading process, we can study the impact of the isolation rate on the infection profile and further analyze its effect on the basic reproduction number. In addition, the method assumes that the probability of a test giving a correct result is constant (one, in this work) and uniform, and does not change with the viral load in the host. The infection profile, which captures the spread in a nearly fully susceptible population, is assumed to be constant over the epidemic window we consider.

In order to study the impact of the daily isolation rate α on the basic reproduction number, we define the basic reproduction number under the impact of the isolation rate

α as

$$\mathcal{R}(\alpha) = \sum_{i=1}^{n} v_i(\alpha). \tag{11}$$

Then, we define the scaling factor of the basic reproduction number under the isolation rate α as $\mathcal{F}(\alpha)$, where

$$\mathcal{F}(\alpha) = \frac{\mathcal{R}(\alpha)}{\mathcal{R}}. \tag{12}$$

Based on Eq (12), we can quantify how the isolate rate α scales down the basic reproduction number. From the definition of the scaling factor, we have that $\mathcal{F}(\alpha) \in [0, 1]$. Additionally, we further explore the impact of the isolation rate on the serial interval distribution. Based on the definition of the serial interval distribution in Eq (5), we define the serial interval distribution under the isolation rate α as

$$w(\alpha) = \frac{v(\alpha)}{\mathcal{R}(\alpha)} = \frac{v(\alpha)}{\mathcal{F}(\alpha)\mathcal{R}}. \tag{13}$$

Both w and $w(\alpha)$ are probability distributions. Hence, the isolation rate α only changes the shape of the distribution but not the summation.

We use an example to illustrate the defined scaling factor $\mathcal{F}(\alpha)$. Consider an epidemic follows the given infection profile $v = [0.148, 1.0, 0.823, 0.426, 0.202, 0.078, 0.042, 0.057, 0.009]$, with the basic reproduction number $\mathcal{R} = 2.785$. We consider two testing-for-isolation strategies: testing and then isolating 100% of the total population weekly, uniformly split over seven days. Testing and isolating the entire population on one day can prevent the virus from spreading. However, it is challenging to implement such a strategy due to limited testing capacity. For instance, UIUC implemented a high testing-for-isolation rate, such that everyone was tested two or three times a week. Nevertheless, these tests at UIUC were still split over the week, as shown in S2 Fig in SI, which gives us $\alpha_1 = 1/7$. Testing and isolating 10% of the total population weekly, uniformly split over seven days, give us $\alpha_2 = 0.1/7$. We apply these isolation rates on the infection profile v using the proposed mechanism in Eq (10), and as a result, we obtain the following scaled infection profiles

$$v(\alpha_1) = [0.127, 0.735, 0.518, 0.223, 0.0935, 0.031, 0.014, 0.017, 0.002],$$
$$v(\alpha_2) = [0.146, 0.972, 0.7882, 0.402, 0.188, 0.072, 0.038, 0.051, 0.008].$$

We plot the original infection profile v and the two infection profiles ($v(\alpha_1)$ and $v(\alpha_2)$) under the daily isolation rates $\alpha_1 = 1/7$ and $\alpha_2 = 0.1/7$ in S15 Fig in SI. Additionally, we compute the basic reproduction number under the two different isolation rates, resulting in $\mathcal{R}(\alpha_1) = 1.77$ and $\mathcal{R}(\alpha_2) = 2.66$. The corresponding scaling factors are $\mathcal{F}(\alpha_1) = 0.64$ and $\mathcal{F}(\alpha_2) = 0.95$, respectively. Consequently, testing then isolating the entire population weekly can reduce the basic reproduction number of the spreading process, as captured by the infection profile in Eq (3), by 36%. Similarly, a lower isolation rate, such as isolating 10% of the population weekly, can reduce the basic reproduction number of the spreading process by 5%. These findings demonstrate the impact of the isolation rate on the basic reproduction number. Next, we utilize the proposed method to generate synthetic infection data, estimate the effective reproduction number, evaluate the impact of different isolation rates for hypothetical spreading scenarios, and design a feedback control algorithm that takes into account the influence of the isolation rate.

SI-2-D. Estimation of the Effective Reproduction Number

The key metric we utilize to generate synthetic infection data is the effective reproduction number. To accomplish our objective of reconstructing spreading processes

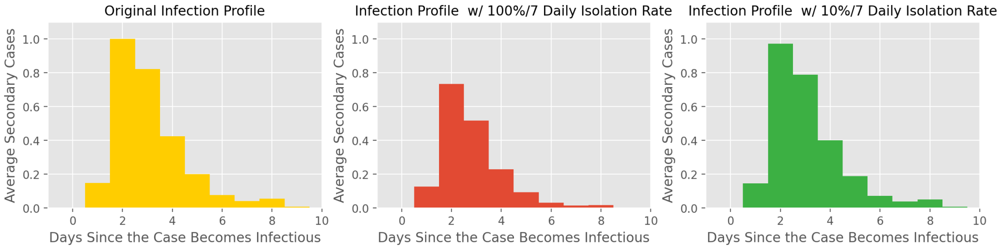

Fig S15. Infection profiles: (Left) Original infection profile, (Middle) Infection profile under $\alpha_1 = 1/7$ testing/isolation rate, (Right) Infection profile under $\alpha_2 = 0.1/7$ testing/isolation rate. Different isolation rates can reshape the infection profile of the spreading process to varying degrees.

on the UIUC and Purdue campuses, we present the method we employ to estimate the effective reproduction number from the confirmed cases on both campuses. The fundamental methodology we employ for estimating the effective reproduction number is based on the approach outlined in [1, 2, 25] using the *Epyestim* package [19], which is a Python package that builds upon the *R* package EpiEstim [1]. As the development of methodologies for estimating the effective reproduction number is not the main focus of our work, we provide only a brief introduction of the core techniques developed in [1]. Moreover, the techniques presented in [1] can be applied to infected cases without considering the incubation period and test delays that we have introduced. Therefore, to implement the estimation techniques described in [1], the frameworks presented in [2] provide a means to preprocess confirmed cases to fit into the techniques described in [1]. Here, we introduce the general concepts of preprocessing confirmed cases for the estimation of the effective reproduction number from [2] and [19]. For more detailed information and discussions on methods and challenges in estimating the effective reproduction number, refer to [1, 2, 19, 26–29].

SI-2-D-1. Pre-processing Confirmed Infection Data

One essential step for estimating the effective reproduction number via EpiEstim [25] involves preprocessing the confirmed infection data. We can consider the estimation of the effective reproduction number from the confirmed cases (e.g., S3 and S8 Figs in SI) as a reverse process of generating synthetic infection data in Section SI-2-B. In contrast to Section SI-2-B, where we leverage the known effective reproduction number to generate synthetic confirmed cases, we use real-world confirmed cases to estimate the effective reproduction number that generated such confirmed data. Recall that, the Poisson process in Eqs (7) and (8) can generate infected data. However, real-world spreading data, including data from S3 Fig and S8 Fig in SI are reported as confirmed cases. Therefore, in order to obtain the effective reproduction number via Eqs (7) and (8), we need to preprocess the real-world spreading data to obtain the infected cases. As introduced in [2], we summarize the core data preprocessing procedures in terms of data smoothing and deconvolution. To smooth the confirmed cases, [2] implemented a local polynomial regression (LOESS) with first-order polynomials and tricubic weights. In our settings, we included a 21-day window of confirmed cases in the local neighborhood of each point as our smoothing parameter. Further, [2] extended the deconvolution method of [1, 25, 30], which is itself an adaptation of the Richardson-Lucy algorithm [31, 32], to obtain the infected cases based on the confirmed cases under the delay distributions from the incubation period and infection-to-confirmation delays. A more detailed discussion about data preprocessing can be found in [2].

SI-2-D-2. Bayesian Inference

We utilize Bayesian inference to estimate the effective reproduction number, as proposed by [1–3]. Again, we leverage the Python package *EpyEstim* [19] inspired by [1, 2, 25] to estimate the effective reproduction number from confirmed cases. In this section, we introduce some key notations for the Bayesian inference framework. For more comprehensive details, please refer to [1–3]. We again assume that the infection profile, along with its serial interval distribution, is independent of calendar time and is solely influenced by the testing-for-isolation strategy. In Section SI-2-B, the new infected cases are modeled as a Poisson process, where the average number of new infected cases at time t follows a Poisson distribution with a mean of $\mathcal{R}_t \sum_{s=1}^{t} I_{t-s} w_s$. Hence, if the number of new infected cases at time t is given by I_t, the likelihood of the infected cases I_t given the unknown effective reproduction number $\mathcal{R}_t$, conditional on the previous infected cases $\{I_0, \ldots, I_{t-1}\}$ is

$$\mathbb{P}(I_t \mid I_0, \ldots, I_{t-1}, w, \mathcal{R}_t) = \frac{(\mathcal{R}_t \Lambda_t)^{I_t} e^{(-\mathcal{R}_t \Lambda_t)}}{I_t!}, \tag{14}$$

where $\Lambda_t = \sum_{s=1}^{t} I_{t-s} w_s$ [1].

In the real-world spreading process, the effective reproduction number usually varies over time. To describe the spreading process within a short time period, we can leverage the effective reproduction number during a sliding window of a certain time period. Hence, we leverage an estimated effective reproduction number $\mathcal{R}_{t,\tau}$ to represent the average effective reproduction number over a time period $[t - \tau + 1, t]$. The likelihood of the new infected cases during the time period $[t - \tau + 1, t]$, $\{I_{t-\tau+1}, \ldots, I_t\}$, given the effective reproduction number $\mathcal{R}_{t,\tau}$, conditional on the previous infected cases $\{I_0, \ldots, I_{t-\tau}\}$, is

$$\mathbb{P}(I_{t-\tau+1}, \ldots, I_t \mid I_0, \ldots, I_{t-\tau}, w, \mathcal{R}_{t,\tau}) = \prod_{s=t-\tau+1}^{t} \frac{(\mathcal{R}_{t,\tau} \Lambda_s)^{I_s} e^{(-\mathcal{R}_{t,\tau} \Lambda_s)}}{I_s!}. \tag{15}$$

In the framework of Bayesian inference, the likelihood function in Eq (15) follows a Poisson distribution. Therefore, it is natural to consider choosing the prior distribution of $\mathcal{R}_{t,\tau}$ as the conjugate prior of a Poisson distribution, which is a Gamma distribution with parameters (a, b). Using a posterior joint distribution of $\mathcal{R}_{t,\tau}$, the posterior joint distribution of $\mathcal{R}_{t,\tau}$ [1, 25], is given by:

$$\begin{aligned}&\mathbb{P}(I_{t-\tau+1}, \ldots, I_t, \mathcal{R}_{t,\tau} \mid I_0, \ldots, I_{t-\tau}, w)\\ &= \mathcal{R}_{t,\tau}^{a+\sum_{s=t-\tau+1}^{t}(I_s-1)} e^{-\mathcal{R}_{t,\tau}(\sum_{s=t-\tau+1}^{t} \Lambda_s + \frac{1}{b})} \prod_{s=t-\tau+1}^{t} \frac{\Lambda_s^{I_s}}{I_s!} \frac{1}{\Gamma(a) b^a},\end{aligned}$$

which is proportional to $\mathcal{R}_{t,\tau}^{a+\sum_{s=t-\tau+1}^{t}(I_s-1)} e^{-\mathcal{R}_{t,\tau}(\sum_{s=t-\tau+1}^{t} \Lambda_s + \frac{1}{b})} \prod_{s=t-\tau+1}^{t} \frac{\Lambda_s^{I_s}}{I_s!}$. Therefore, the posterior distribution of $\mathcal{R}_{t,\tau}$ is a Gamma distribution with parameters $(a + \sum_{s=t-\tau+1}^{t} I_s, \frac{1}{\sum_{s=t-\tau+1}^{t} \Lambda_s + \frac{1}{b}})$. Further, the posterior mean of $\mathcal{R}_{t,\tau}$ is $(\frac{a+\sum_{s=t-\tau+1}^{t} I_s}{\sum_{s=t-\tau+1}^{t} \Lambda_s + \frac{1}{b}})$ [1, 25]. Again, for a detailed discussion of the Bayesian inference for the effective reproduction number, we refer readers to [1, 2, 19, 21–23, 25]. Additionally, [3] and [27] discuss the advantages and limitations of current widely-used estimation methods for the effective reproduction number.

SI-2-D-3. Estimation of the Effective Reproduction Number over Campuses

In this section, we leverage *EpyEstim*, which combines the data pre-processing method from [2] with Bayesian inference techniques to estimate the effective reproduction

number using confirmed cases from UIUC and Purdue. Both UIUC and Purdue implemented testing-for-isolation strategies, which require modifications to the initial serial interval distribution given in Eq (6). Specifically, for UIUC, where the daily isolation rate equals the daily testing rate, i.e., $\alpha_I = 2/7$ of the population (surveillance testing-for-isolation strategy), we can calculate the modified serial interval distribution for the spreading process at UIUC using the default infection profile from Eqs (3), (10), and (13). We denote this modified serial interval distribution as $w(\alpha_I)$. We use $w(\alpha_I)$ as the serial interval distribution to estimate the effective reproduction number at the UIUC campus. Compared to UIUC, Purdue encouraged symptomatic cases to be self-reported and tested (voluntary testing-for-isolation), while focused on testing and isolating around 10% of the total population to identify asymptomatic cases (surveillance testing). Based on the data from Purdue, we consider the following conditions when estimating the effective reproduction number. We perform sensitivity analysis on the ratio of symptomatic infections θ and the isolation rate on our proposed methodologies.

- The ratio of symptomatic infections $\theta = 0.55$.
- Symptomatic cases are expected to report and get tested and then to be isolated within a week of showing symptoms. Therefore, the isolation rate for symptomatic cases is 100%/7.
- For asymptomatic cases, around 30% of the total asymptomatic cases were tested and isolated through the 10% weekly non-uniformly testing. This condition is based on the fact that the 10% testing excludes symptomatic and suspicious cases, and the contact-tracing network at Purdue makes the testing not uniformly random. Hence, we assume a 30% uniformly applied testing-for-isolation rate for asymptomatic cases at Purdue.
- Both symptomatic and asymptomatic infections have the same infection profile v given by Eq (3).

Based on these conditions, the overall daily isolation rate at Purdue is defined as

$$\alpha_P = \theta \underline{\alpha}_P + (1 - \theta)\overline{\alpha}_P,$$

where $\underline{\alpha}_P$ and $\overline{\alpha}_P$ represent the daily isolation rates for symptomatic and asymptomatic infections, respectively. Hence, we have that $\alpha_P = 100\%/7 \times 0.55 + 30\%/7 \times 0.45 = 68.5\%/7$ per day. Therefore, the overall weekly isolation rate at Purdue is 0.685. Similarly, we can calculate the serial interval distribution of the spreading process at Purdue using Eq (10) and Eq (13) (Eq (12) in the main manuscript), denoted as $w(\alpha_P)$. We use $w(\alpha_P)$ as the serial interval distribution to estimate the effective reproduction number at the Purdue campus.

In the estimation process, we use a sliding window of 21 days to smooth the confirmed cases when estimating the effective reproduction number. We consider that the estimated effective reproduction number can affect new infections in the next seven days, denoted as $\tau = 7$ in Eq (15), such that the policy remained unchanged within a short period. The estimated effective reproduction numbers for UIUC, $\mathcal{R}_t(\alpha_I)$, and Purdue, $\mathcal{R}_t(\alpha_P)$, under their corresponding isolation rates $\alpha_I = 2/7$ and $\alpha_P = 0.685/7$ are illustrated in S16 Fig in SI and S17 Fig in SI, respectively. In S16 Fig and S17 Fig in SI, the solid lines represent the mean values of the estimated effective reproduction number, while the shadow areas represent the 95% confidence interval of the estimated effective reproduction number. Since we use an average 10.3-day infection-to-confirmation delay distribution from being infected to confirmed, there are lags between the estimated effective reproduction number and the confirmed cases.

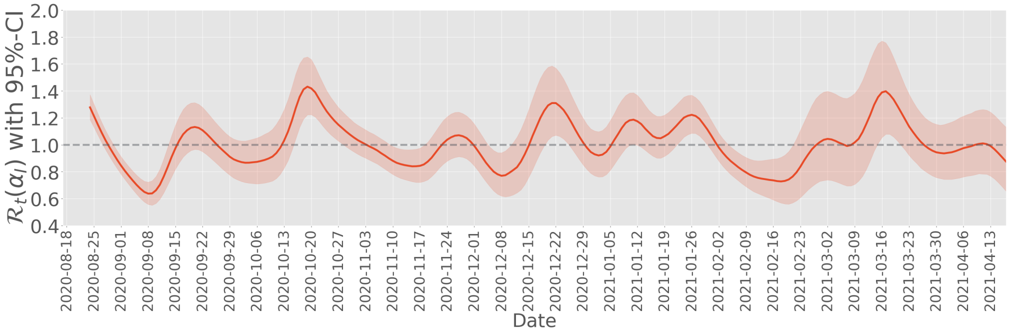

Fig S16. The estimated effective reproduction number of the spreading process over the UIUC campus. The estimated effective reproduction number is greater than one during multiple periods, particularly at the beginning of Fall 2020, around the middle of October 2020, and the middle of Spring 2021. This estimation aligns with the observations from the confirmed cases at UIUC during Fall 2020 and Spring 2021, where several mild spikes were observed.

These lags capture the delays between the infected cases and the corresponding confirmed cases.

For UIUC, the estimated effective reproduction number is greater than one for multiple periods, particularly at the beginning of Fall 2020, around the middle of October 2020, and the middle of Spring 2021. This estimation aligns with the observations from the confirmed cases at UIUC during Fall 2020 and Spring 2021, where multiple mild spikes were observed. The larger spikes during Fall 2020 were caused by the entry-screening at the beginning of the semester and the gathering events during the return of the football season around the middle of October. The larger spike during Spring 2021 was caused by the entry-screening for students returning from the Spring break. Similarly, for Purdue, the estimated effective reproduction number is greater than one for multiple periods during Fall 2020 and Spring 2021. There were two major spikes observed in the estimated effective reproduction number at Purdue. One occurred around the middle of August, and the other around the beginning of January. These two spikes describe the infection process during the Summer and the Winter break, caught by the entry-screening processes. Additionally, these spikes were reflected in the confirmed cases around ten days later, corresponding to the beginning of the Fall 2020 and Spring 2021 semesters, respectively. These estimation results further highlight the importance of the infection-to-confirmation delay distribution, where the spikes observed during the entry-screening at the beginning of the Fall 2020 and Spring 2021 semesters were due to infections that occurred during breaks off-campus.

In summary, the estimated effective reproduction number can reflect the spikes observed in the confirmed cases, and we can connect these spikes to real-world events. These analyses inspire us to use the effective reproduction number as an indicator (feedback) to facilitate the design of the closed-loop feedback control algorithm.

SI-2-D-4. Sensitivity Analysis on Estimating the Effective Reproduction Number

To further analyze the impact of the length of the smoothing windows on the estimation results, we use the confirmed cases from UIUC (S3 Fig in SI) as an example. Fixing $\tau = 7$, we consider smoothing windows of 7, 14, and 28 days and plot the corresponding estimated effective reproduction numbers in S18 Fig in SI. From S18 Fig in SI, we observe that a longer smoothing window generates a smoother estimated effective reproduction number, as it averages out more noise and variations in data collection.

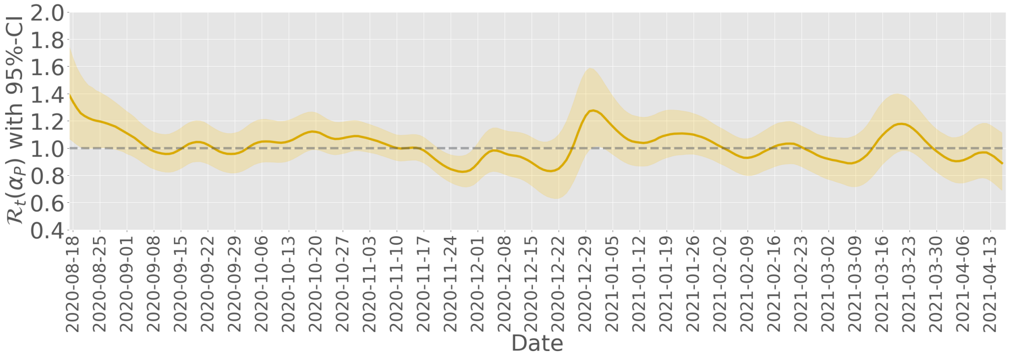

Fig S17. The estimated effective reproduction number of the spreading process over the Purdue campus. The estimated effective reproduction number is greater than one for multiple periods during Fall 2020 and Spring 2021. There were two major spikes observed in the estimated effective reproduction number at Purdue. One occurred around the middle of August, and the other around the beginning of January 2021. These two spikes describe the infection process during the Summer and the Winter break.

All three plots can capture the trend of the epidemic spreading in terms of spikes and plateaus. However, with longer smoothing windows, the estimated effective reproduction numbers become less sensitive to changes in the spreading behavior.

For instance, when using a relatively shorter smoothing window, such as 7 days (the red line in S18 Fig in SI), the estimated effective reproduction number is highly sensitive to changes in the confirmed cases. In contrast, when using a longer smoothing window, such as 28 days (the golden line in S18 Fig in SI), the estimated effective reproduction number is less sensitive to changes in the confirmed cases. It is critical to find a balance between sensitivity and responsiveness in the estimation process to ensure reliable outbreak evaluation and decision-making. Therefore, in this work, we choose a 21-day smoothing window (as shown in S16 Fig in SI) and $\tau = 7$ in our estimation to capture the epidemic trend while maintaining sensitivity to changes in the spreading behavior.

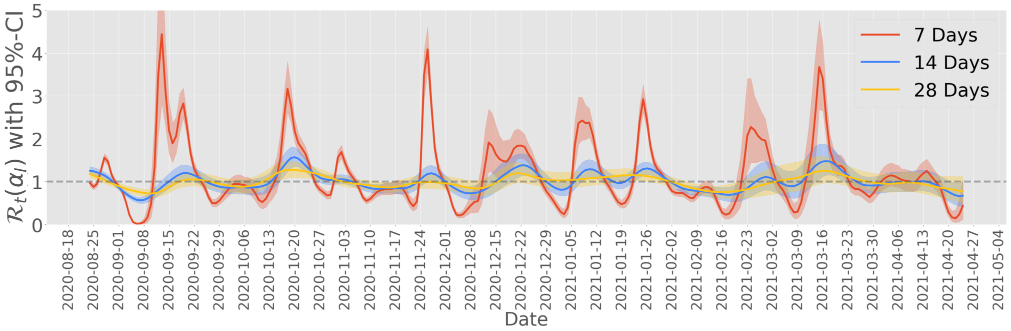

Fig S18. Estimated effective reproduction number of the spreading process over the UIUC campus (7−, 14−, 28−day average data). A longer smoothing window generates a smoother estimated effective reproduction number, as it averages out more noise and variations in data collection.

In addition to analyzing the impact of the data smoothing window, we also study the impact of τ on the estimation results. We maintain a 21−day smoothing window and vary the value of τ to observe its effect. Specifically, we set τ as 14, 21, and 28, respectively, and plot the corresponding estimated effective reproduction number over

the UIUC campus in S19 Fig in SI. From these plots, it is evident that as we increase the value of τ, representing a longer window for estimating the effective reproduction number, the estimation becomes less sensitive to changes. In other words, a longer τ results in a smoother estimated effective reproduction number with reduced sensitivity to short-term fluctuations. Additionally, considering the weekly nature of the confirmed testing data in S3, it is reasonable to consider that the estimated effective reproduction number can exhibit weekly changes in behavior rather than monthly. Hence, to capture the changing trends in the spread of the epidemic, we choose $\tau = 7$ as the value for estimating the effective reproduction number. Additionally, the estimated effective reproduction number's accuracy is influenced by several factors, such as the estimation algorithm, the choice of distribution, and the data collection process, among others. More detailed discussions on this topic are given in [1, 3, 25, 33–35].

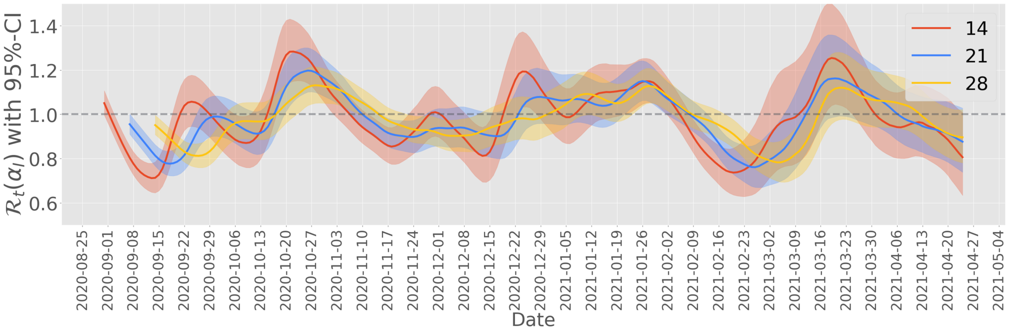

Fig S19. Estimated effective reproduction number of the spreading process over the UIUC campus ($R_{t,\tau=14,21,28}$). A longer window captured by τ for estimating the effective reproduction number reduces sensitivity to short-term fluctuations from weekly data collection dynamics.

SI-2-E. Reconstruction of Spreading Processes Over Campuses

In order to create an environment for validating the proposed framework, we reconstruct the spreading processes over both campuses using the methods introduced in Section SI-2-C and the estimated effective reproduction number from both campuses in Section SI-2-D. We consider two reconstruction scenarios. First, we reconstruct the spreading processes over both campuses under their implemented testing-for-isolation strategies using the methods introduced and proposed in Sections SI-2-B, SI-2-C, and SI-2-D. This approach integrates the estimation techniques and testing-for-isolation strategies discussed earlier. Next, we introduce the method to reverse engineer the effective reproduction number of the real-world spread under the implemented isolation rate to compute the effective reproduction number of a hypothetical spread with alternative isolation rates, including zero. We leverage this method to reconstruct hypothetical spreading scenarios on both campuses under the assumption that the universities had not implemented their testing-for-isolation strategies during Fall 2020, aiming to conduct a counterfactual analysis of the potential outbreak. By comparing the reconstruction results between the two scenarios, we aim to evaluate the impact of the testing-for-isolation strategies on the spreading processes and emphasize the significance of implementing these strategies for mitigating the spread of the epidemic.

SI-2-E-1. Reconstructing the Original Spread

We utilize the estimated effective reproduction number in S16 Fig in SI and S17 Fig in SI in Section SI-2-D along with the simulation mechanism introduced in Section SI-2-B

to generate synthetic data that aligns with the real-world confirmed cases on both campuses, as shown in S3 and S8 Figs in SI. We begin by reconstructing the spreading process over the UIUC campus. We leverage the estimated effective reproduction number shown in S16 Fig in SI, along with the modified serial interval distribution $w(\alpha_I)$ under the overall daily isolation rate α_I, and the infection-to-confirmation delay distribution Δ given in Section SI-2-B. The resulting reconstruction is illustrated in S20 Fig in SI with a dotted, solid line, which closely matches the confirmed cases observed over the UIUC campus during Fall 2020 and Spring 2021. Notably, the reconstruction accurately captures the spreading trend, including spikes and weekly confirmed patterns. In order to eliminate the impact of the Winter break, when most people were not on campus, we simulate the spreading process over Fall 2020 and Spring 2021 separately, since at the beginning of each semester, the entry-screening resets the spreading process. In addition, UIUC implemented different isolation rates during Fall 2020 (two times a week) and Spring 2021 (three times a week).

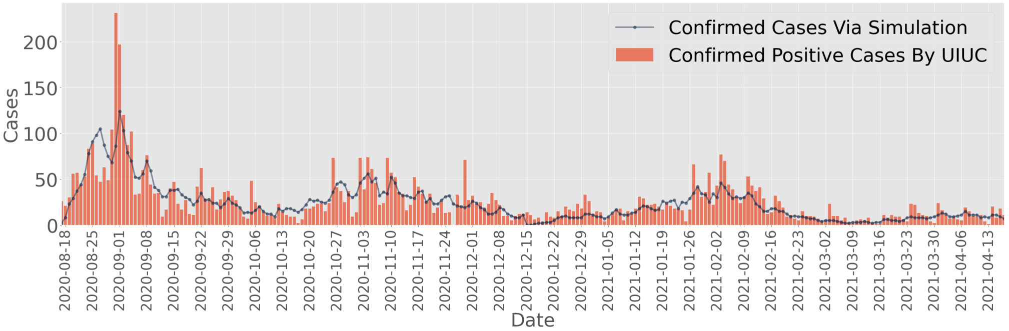

Fig S20. Reconstructed spreading process over the UIUC campus. The simulated spreading process accurately captures the spreading trend, including spikes and weekly confirmed pattern.

We apply the same techniques to reconstruct the spreading process over the Purdue campus. However, we utilize a different modified serial interval distribution, $w(\alpha_P)$, which is determined based on Purdue's testing-for-isolation strategy. We continue to use the same infection-to-confirmation delay distribution, Δ, used for UIUC. S21 Fig in SI illustrates the reconstruction (dotted, solid line) that matches the confirmed cases observed on the Purdue campus during Fall 2020 and Spring 2021. Further, the reconstructed spreading process accurately captures the major spikes observed in the confirmed cases. In contrast to UIUC where we reset the initial condition at the beginning of the Spring 2021 semester, we did not reset the initial condition for Purdue during the same period. Therefore, the impact of the entry-screening at the beginning of Spring 2021 is not considered, resulting in an overestimation of the daily infected cases during the Spring 2021 semester at Purdue.

In summary, the reconstruction process relies on the estimated effective reproduction number, distributions, and parameters utilized during the estimation process. Different distributions and parameters may result in varied estimations of the effective reproduction number. However, when the conditions for estimation remain constant, the generation of synthetic data for reconstruction acts as an inverse process of the estimation, aligning the reconstructed spread with the real-world spreading data.

SI-2-E-2. Evaluating the Spreading Process without Isolation

After reconstructing the spreading process under the implemented testing-for-isolation strategies over both campuses, we study the scenario where what would have happened

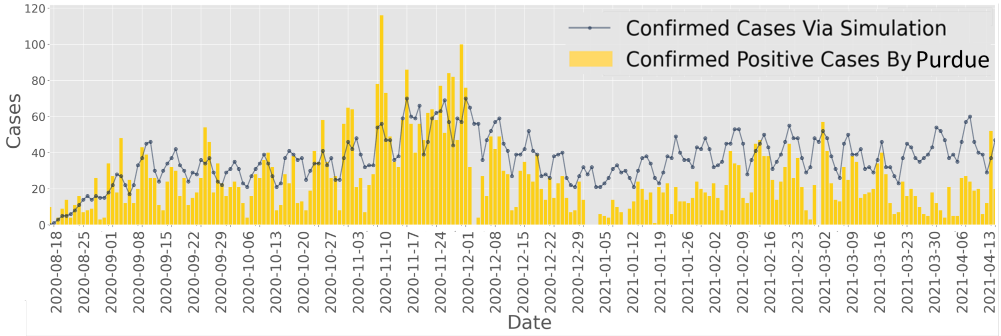

Fig S21. Reconstructed spreading process over the Purdue campus. The reconstruction (dotted, solid line) matches the confirmed cases observed on the Purdue campus during Fall 2020 and Spring 2021. In contrast to UIUC, where we reset the initial condition at the beginning of the Spring 2021 semester, not resetting the initial condition for Purdue during the same period resulted in an overestimation of the daily infected cases during the Spring 2021 semester at Purdue.

if both universities had not implemented their testing-for-isolation strategies. This type of epidemic reconstruction process will also provide a foundation for evaluating hypothetical spreading scenarios under different intervention strengths, including varying the isolation rate. First, we propose a novel method, i.e., reverse engineering the effective reproduction number, to facilitate the reconstruction of the hypothetical outbreak over campuses without the implemented testing-for-isolation strategies. Consider that all the conditions remain exactly the same during the spreading period of interest, such as the spreading environment, other intervention policies, among others. Further, we assume that the change in the strength of the intervention strategy does not further impact students' behavior, viral loads, or other factors. The only difference is that the spreading processes will be affected by a different isolation rate, including a zero isolation rate. To account for this modification, we can extend the basic reproduction number scaling method we proposed in Eq (12) to compute $\mathcal{R}_t(\alpha = 0)$ based on the effective reproduction number $\mathcal{R}_t(\alpha)$ under the implemented daily isolation rate $\alpha = \alpha_I$ or $\alpha = \alpha_P$.

Besides using Eq (12) to compute the scaling factor, which is introduced in the main manuscript, we can also compute $\mathcal{F}(\alpha)$ by using the serial interval distribution. We denote the serial interval distribution of the spreading process as $w \in \mathbb{R}^n$. According to the method that quantifies the impact of the isolation rate on the infection profile v, introduced in SI-2-C, we can define the pseudo-serial interval distribution under the isolation rate α as

$$w^*(\alpha) = [w_1(1-\alpha), w_2(1-\alpha)^2, \cdots, w_n(1-\alpha)^n]. \tag{16}$$

Note that $\sum_{i=1}^n w_i^*(\alpha) \leq 1$, since $\sum_{i=1}^n w_i = 1$. Hence, we call $w^*(\alpha)$ a pseudo-serial interval distribution instead of a serial interval distribution. We show that Eq (16) provides a way of using $w^*(\alpha)$ to compute the impact of the isolation rate on the serial interval distribution directly, through the scaling factor $\mathcal{F}(\alpha)$. The connection is given by

$$\mathcal{F}(\alpha) = \frac{\mathcal{R}(\alpha)}{\mathcal{R}} = \frac{\mathcal{R}\sum_{i=1}^n w_i^*(\alpha)}{\mathcal{R}\sum_{i=1}^n w_i} = \sum_{i=1}^n w_i^*(\alpha). \tag{17}$$

In Eq (17), we use the fact that $w_i^*(\alpha) \times \mathcal{R} = w_i^*(1-\alpha)^i \times \mathcal{R} = v_i(1-\alpha)^i = v_i(\alpha)$, $i \in \{1, 2, \ldots, n\}$ and $\sum_{i=1}^n v_i(\alpha) = \mathcal{R}(\alpha)$. Therefore, even without the knowledge of the estimated effective reproduction number, we can still compute the impact of the

isolation rate by using the scaling factor $\mathcal{F}(\alpha)$, given the serial interval distribution of the spreading process. The proposed method in Eq (17) provides a foundation for reverse engineering the effective reproduction number of an outbreak for a hypothetical spreading scenario under an alternative isolation rate, where we can compute the scaling factor $\mathcal{F}(\alpha)$ by directly scaling the serial interval distribution w.

Consider that the epidemic spreading processes without any testing-for-isolation strategies over both campuses follow the initial serial interval distribution w as shown in S11 Fig in SI (Right). Consider that UIUC tested the entire campus twice a week during Fall 2020 and these tests were evenly distributed throughout the week, we calculate the daily isolation rate for UIUC as $\alpha_I = 2/7 = 0.286$. Furthermore, using Eq (17) we compute $\mathcal{F}(\alpha_I) = 0.3885$. Based on our analyses, it is natural to believe that the effective reproduction number of the hypothetical outbreak without the implemented testing-for-isolation strategies would be the estimated one given in S16 Fig in SI divided by $\mathcal{F}(\alpha_I) = 0.3885$. However, by directly following this procedure, we may overlook a critical factor.

In SI-2-A-1, we consider the infection profile and its serial interval distribution, which correspond to the basic reproduction number. This assumes that the population size is sufficiently large and that the infection profile is generated in a nearly fully susceptible population. For densely populated university campuses where the total number of population is fixed, the infected population can be large, so we need to consider the impact of the susceptible population size on scaling the effective reproduction number. Similar to the idea that the effective reproduction number can be obtained by scaling the basic reproduction number via the proportion of the existing susceptible population (Eq (4)), we propose the following mechanism to compute the effective reproduction number $\hat{\mathcal{R}}_t$ of the hypothetical outbreak without any implemented isolation rate α_I at UIUC, as shown in Section 4.3 of the main manuscript. This is applied when reverse engineering the effective reproduction number under the condition that the population is not sufficiently large:

$$\hat{\mathcal{R}}_t = \frac{\mathcal{R}_t(\alpha_I)\hat{S}(t)}{\mathcal{F}(\alpha_I)S(t)}, \tag{18}$$

where $N \in \mathbb{N}_{\geq 0}$ is the total population, $S(t) \in [0, N]$ is the number of susceptible individuals in the simulated outbreak for the spread with the overall isolation rate α_I on the UIUC campus at time t, $\hat{S}(t) \in [0, N]$ is the number of susceptible individuals in the simulated outbreak for the hypothetical spread with zero isolation rate on the UIUC campus at time t, and $\mathcal{R}_t(\alpha_I)$ is the estimated effective reproduction number of the real-world outbreak at time t, under the overall daily isolation rate α_I.

The total population on the UIUC campus during Fall 2020 is approximately $N = 50,000$ and we have the scaling factor $\mathcal{F} = \mathcal{F}(\alpha_I) = 0.3885$. We simulate two spreading processes simultaneously. The first spread is simulated by leveraging the estimated effective reproduction number at UIUC, $\mathcal{R}_t(\alpha_I)$, allowing us to reconstruct the spread at UIUC, as shown in SI-2-E-1 (i.e., the solid line in S20 Fig in SI). Meanwhile, we simulate the second spread, representing the hypothetical outbreak without the daily isolation rate α_I. We first compute the reverse engineered reproduction number $\hat{\mathcal{R}}_t$ simultaneously from the ongoing simulation of the first spread, and then we use the reverse engineered reproduction number along with the same infection data generation mechanism in SI-2-B to simulate the hypothetical outbreak. To capture the worst-case scenario, we explore the hypothetical scenario where the infected population behaves as if they were uninfected, either due to indifference or lack of awareness. This scenario is akin to assuming that all infected individuals are asymptomatic, and requires that the change in the strength of the intervention strategy does not further impact students' behavior, viral loads, or other factors. In this scenario,

we perform testing but no isolation, only recording the confirmed cases. Consequently, the infected population spreads the virus throughout the full infectious period, adhering to the serial interval distribution represented by w from S11 Fig in SI (Left). These parameters depict a potential worst-case scenario for COVID-19 spreading across the UIUC campus, as no infectious cases are isolated.

The reverse engineering method, as shown in Eq (18), requires the estimation of the susceptible population, $S(t)$ at each time step t in the real-world outbreak. Based on the testing-for-isolation strategy, we can estimate the current susceptible population, $S(t)$, by subtracting the new daily infected population from the total population N prior to the time step t. Under the assumption that testing is uniformly sampled from the entire population, with no loss of immunity during the epidemic window of study, we can estimate the total new daily infected population based on the sample population and the sampling rate. This information allows us to determine the existing susceptible population $S(t)$ in the real-world spreading scenario. There may be more accurate ways to estimate the susceptible population even in the presence of delays, such as delays in testing-to-report or the incubation period of infected cases. However, since estimating the susceptible population is not the primary focus of our work and represents an entire field of research in itself, we only briefly comment on potential methods for estimating the susceptible population from daily case reports.

When computing the susceptible population $\hat{S}(t)$ in the simulation environment, we first generate infected cases based on the generation time interval and then add delays and noise patterns to imitate real-world spreading scenarios (SI-2-B). We can record the new (daily) infected cases as they are generated. Then, we subtract the daily infected cases from the total population N to obtain the daily susceptible population. In this work, when we reverse engineer the effective reproduction number for the hypothetical spread, we simulate both outbreaks simultaneously to generate both the real-world and hypothetical spreading scenarios. This approach allows us to estimate both $\hat{S}(t)$ and $S(t)$ in the simulation, as we can directly obtain the infected cases in the simulated hypothetical spreading environment and then compute the susceptible population.

We generate the 'confirmed cases' without employing any testing-for-isolation strategies, as depicted in S22 Fig in SI by the solid line in dark blue. These cases are confirmed positive through testing but no isolation actions are taken. Although we label these instances as 'confirmed cases,' in the simulation, they are not actually isolated from the population. These cases are recorded by applying the same delay distributions to the infected cases, facilitating a comparison with the simulated daily confirmed cases under the implemented testing-for-isolation strategy at UIUC, represented by the red solid line. In S23 Fig in SI, the corresponding effective reproduction number (95% confidence interval) is presented. The marked line in dark blue illustrates the effective reproduction number of the hypothetical spreading process without the implementation of testing-for-isolation strategies. The red marked line represents the estimated effective reproduction number from UIUC data featuring the implemented testing-for-isolation strategy, as shown in S16 Fig in SI.

S22 Fig in SI illustrates that without any isolation strategies and under the assumption that the change in the strength of the intervention strategy does not further impact students' behavior, viral loads, or other factors, the entire population on the UIUC campus would have been infected approximately two months after the start of the Fall 2020 semester. In comparison to the confirmed cases shown in S3 Fig in SI, where the peak number of confirmed cases was under 200, we observe a significant spike in daily confirmed cases, eventually exceeding $3,000$ cases. In addition to studying the confirmed cases, based on the reverse engineered effective reproduction number $\hat{\mathcal{R}}_t$ in S22 Fig in SI, we find that during the first month of the Fall 2020 semester, the reverse engineered effective reproduction number without testing-for-isolation strategies is

consistently higher than the estimated effective reproduction number $\mathcal{R}_t(\alpha_I)$ obtained from real-world spreading data on campus. This phenomenon is caused by the fact that in the real-world scenario, the implementation of testing-for-isolation strategies, specifically testing everyone then isolating all confirmed cases twice a week, reshaped the infection profile of COVID-19 spreading at UIUC. Therefore, without the testing-for-isolation strategy, according to Eq (18), the reverse engineered effective reproduction number would have been scaled up. However, after mid-September 2020, due to the fact of a fixed total population on campus ($N = 50,000$) and the assumption of no short-term loss of immunity, the lack of the sufficient susceptible population of the hypothetical outbreak with zero isolation rate, outweighs the impact of the scaling factor from the testing-for-isolation strategies, as captured by the term $\hat{S}(t)/S(t)$ in Eq (18). As a result, the reconstructed population of confirmed cases in S22 Fig in SI begins to decrease, along with the reverse engineered effective reproduction number dropping below one in S23 Fig in SI. This situation can be explained by the pandemic reaching the point of herd immunity, where the number of new confirmed cases naturally decreases due to a sufficiently small susceptible population.

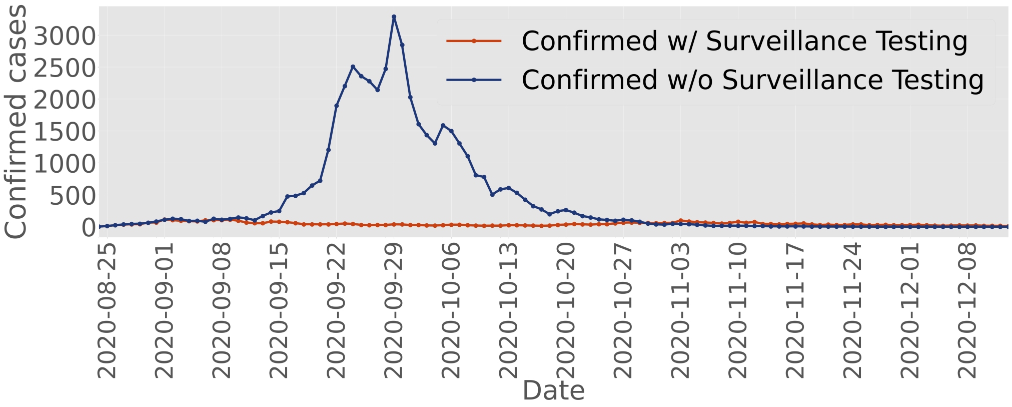

Fig S22. Confirmed cases over the UIUC campus w/ and w/o testing-for-isolation strategies. The solid dark blue line represents the simulated 'confirmed cases' without employing any testing-for-isolation strategies, where these infected cases are confirmed positive through testing but no isolation actions are taken. The solid red line represents the simulated 'confirmed cases' under UIUC's testing-for-isolation strategy.

We apply the same method (Eq (18)) to reconstruct the spread over the Purdue campus without the surveillance testing-for-isolation strategy, i.e., we implement surveillance testing but the tested positive cases take no actions. The same as UIUC, we assume that the change in the strength of the intervention strategy does not further impact students' behavior, viral loads, or other factors. In reality, Purdue implemented a surveillance testing-for-isolation strategy by sampling approximately $50,000$ individuals on campus each week. However, in this case, we consider that there was no isolation for asymptomatic cases under the surveillance testing, and symptomatic cases would be tested and isolated at a daily testing and isolation rate of $\underline{\alpha}_P = 1/7$ through voluntary testing-for-isolation. Although the daily surveillance testing-for-isolation rate is $0.1/7$, we acknowledge that testing may have been influenced by contact tracing strategies and population behaviors, as introduced in Section SI-1-B. Consequently, the rate will be biased and may not precisely reflect 10% per week as intended. Hence, to align with our effective reproduction number estimation process, we consider a 30% weekly isolation rate from surveillance testing, indicating that the 10% weekly surveillance testing-for-isolation strategy identified at least 30% of asymptomatic infections weekly at Purdue, evenly split across the week. Therefore, we have

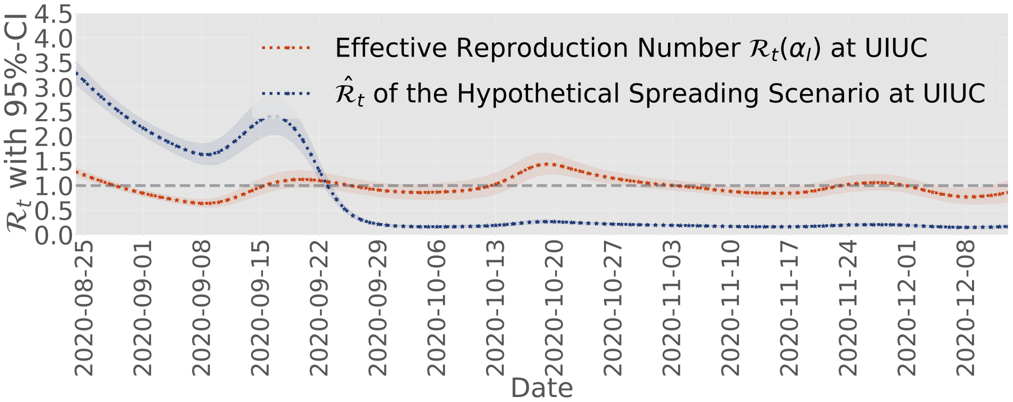

Fig S23. The effective reproduction number at the UIUC campus w/ and w/o isolation strategies. The dashed, dark blue line illustrates the reverse engineered effective reproduction number of the hypothetical spreading process without the isolation strategies. The dashed, red line represents the estimated effective reproduction number obtained from the data at UIUC featuring the implemented testing-for-isolation strategy.

$\overline{\alpha}_P = 0.3/7$.

S24 Fig in SI illustrates that without any isolation under the surveillance testing and under the condition that symptomatic infections are isolated through voluntary testing-for-isolation strategy, there would have been a much larger outbreak at Purdue during Fall 2020. The peak infection value nearly tripled. Compared to the hypothetical spreading process at UIUC, the outbreak at Purdue during Fall 2020 is less severe since all symptomatic cases are caught and isolated through the voluntary testing-for-isolation strategy, under the daily isolation rate $\underline{\alpha}_P = 1/7$. In addition to studying the confirmed cases, based on the effective reproduction number in S25 Fig in SI, we find that during the first three months of the Fall 2020 semester, the reverse engineered effective reproduction number $\hat{\mathcal{R}}_t$ without the isolation under surveillance testing ($\overline{\alpha}_P = 0$) is consistently higher than the estimated effective reproduction number $\mathcal{R}_t(\alpha_P)$ obtained from real-world spreading data on campus. Similar to UIUC, after November 2020, the fixed total population on campus ($N = 50,000$) that results in the lack of a sufficient susceptible population outweighs the impact of the scaling factor from the testing-for-isolation strategies, as captured by the term $\hat{S}(t)/S(t)$ in Eq (18). As a result, the confirmed cases of the hypothetical outbreak without the surveillance testing-for-isolation in S24 Fig in SI start to decrease, along with the reverse engineered effective reproduction number becoming smaller than the estimated effective reproduction number derived from COVID-19 data from Purdue, as shown in S25 Fig in SI.

The results illustrate that the COVID-19 pandemic could have been far worse on both campuses, in terms of huge peak infection values, which would have collapsed the health-care systems, and a larger infected population, which would have forced the schools to close. Therefore, in order to safely operate communities such as large universities during a pandemic, it is critical to implement testing-for-isolation strategies with a sufficiently large isolation rate. Further, we reconstruct the possible worst-case hypothetical scenario at the UIUC campus. For the Purdue campus, we consider a zero isolation rate for asymptomatic cases, while all symptomatic cases, detected via voluntary testing, will isolate themselves. The reconstruction processes are heavily influenced by various conditions and assumptions, such as no change in the viral load. Since we have no further information on how confirmed cases behaved or the exact

isolation rate of the testing-for-isolation strategies, we provide possible scenarios to primarily illustrate the proposed reverse engineering effective reproduction number method for hypothetical outbreaks with zero isolation rate, as shown in Eq (18). There could be other hypothetical scenarios in the reconstruction process under different conditions and assumptions. We further discuss more reconstructions of the hypothetical spreading processes over UIUC and Purdue campuses under different conditions.

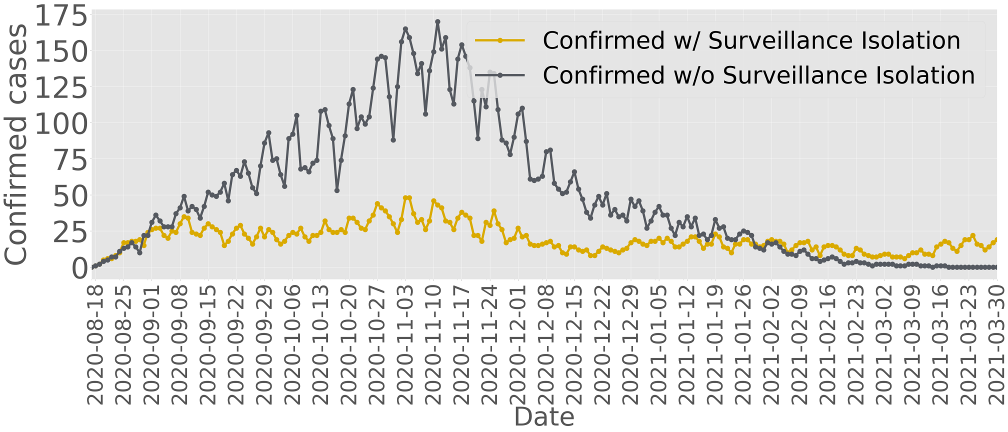

Fig S24. Confirmed cases at the Purdue campus w/ and w/o isolation under the surveillance testing. Compared to the simulated hypothetical spreading scenario at UIUC, the outbreak at Purdue is less severe since all symptomatic cases are caught and isolated through the voluntary testing-for-isolation strategy, with the daily isolation rate $\underline{\alpha}_P = 1/7$.

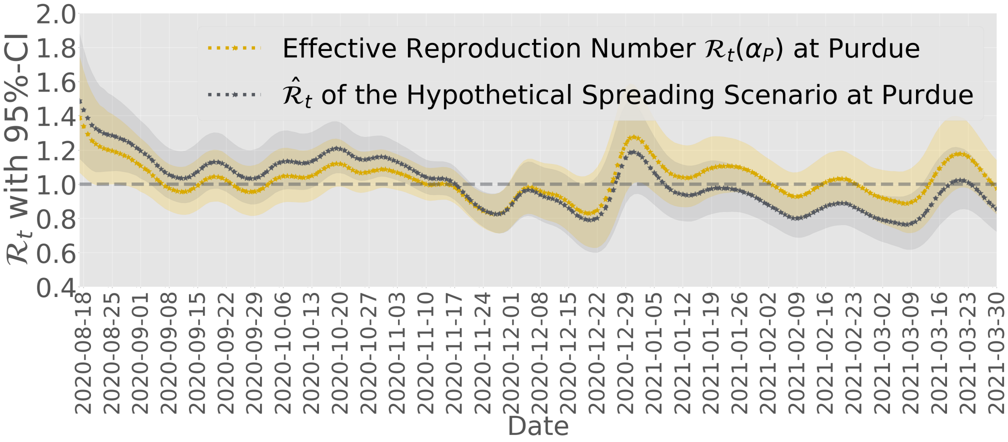

Fig S25. The effective reproduction number at the Purdue campus w/ and w/o isolation under the surveillance testing. During the first three months of the Fall 2020 semester, the reverse engineered effective reproduction number without isolation under surveillance testing (i.e., $\overline{\alpha}_P = 0$) consistently exceeds the estimated effective reproduction number obtained from real-world spreading data on campus. After November 2020, the reduction in the susceptible population of the hypothetical outbreak leads to the reverse engineered effective reproduction number becoming smaller than the estimated effective reproduction number derived from the Purdue COVID-19 data.

SI-2-E-3. Sensitivity Analysis on Reconstruction and Evaluation

The reconstruction and evaluation on the UIUC campus considers the worst-case scenario where all confirmed cases in S3 Fig in SI isolated themselves after being caught by the testing, and none of these confirmed cases isolate themselves from the population without the testing-for-isolation strategy. Here, the sensitivity analysis on the reconstruction of the hypothetical outbreak at the UIUC campus studies what would happen without the isolation if α_I confirmed cases had isolated themselves from the total population, where the overall daily isolation rate satisfies $\alpha_I \in \{10\%/7, 20\%/7, \ldots, 90\%/7\}$ and $\alpha_I \in \{100\%/7, 120\%/7, \ldots, 200\%/7\}$. Based on Eq (18), a lower scaling factor $\mathcal{F}(\alpha_I)$ results in a higher reverse engineered effective reproduction number. For instance, with a 200% weekly testing rate at UIUC, only 100% of the confirmed cases isolated themselves from the population weekly, reflecting an $\alpha_I = 100\%/7$ daily isolation rate. Consequently, without the testing-for-isolation strategy, the remaining 100% of confirmed cases not isolated from the population remain unaffected each week. The testing-for-isolation influences only half of the confirmed cases. In an extreme scenario where $\alpha_I = 0\%/7$ of confirmed cases isolated themselves, S3 Fig in SI would record only the confirmed cases at UIUC under the testing-for-isolation strategy. These infected individuals can still spread the virus freely. Hence, under this assumption, even without the testing, the spreading dynamics would not change.

Based on this concept, S26 Fig in SI captures hypothetical scenarios for the implemented daily isolation rates $\alpha_I \in \{10\%/7, 20\%/7, \ldots, 90\%/7\}$ and $\alpha_I \in \{100\%/7, 120\%/7, \ldots, 200\%7\}$ illustrating that even if UIUC implemented a 200%/7 daily isolation rate, only a proportion of the confirmed cases were isolated. Hence, we analyze hypothetical spreading scenarios to explore what would have occurred if the assumed implemented isolation rate α_I in the outbreak at UIUC had not been implemented. S26 Fig in SI illustrates that the more confirmed cases follow the isolation strategy upon testing positive during Fall 2020 and Spring 2021, the more severe the spread would be without the testing-for-isolation strategy. When $\alpha_I = 200\%/7$ in S26 Fig in SI (where all confirmed cases follow the isolation rule), the reconstruction generates the worst-case scenario, seen in S22 Fig in SI. We plot the heatmap of the daily confirmed cases in S27 Fig in SI, and the cumulative confirmed cases in S28 and S29 Figs in SI.

Our counterfactual analysis of the hypothetical outbreaks at UIUC, under the assumption that the isolation process may not have been perfectly implemented (with a daily isolation rate $\alpha_P \leq 200\%/7$), highlights the importance of understanding policy execution and population behavior when modeling and analyzing epidemic spread. By analyzing the reconstructed hypothetical outbreaks on the UIUC campus, we illustrate the method of reverse engineering the effective reproduction number while discussing the impact of isolation rates on the spread. The reconstruction processes are heavily influenced by conditions and assumptions, such as no changes in viral load. Therefore, considering various aspects of population behavior is crucial for improving the accuracy and reliability of our models and reconstructions, ultimately leading to more effective strategies for controlling and managing epidemics.

When examining the impact of population behavior at the UIUC campus, we did not differentiate between symptomatic and asymptomatic infections. However, Purdue University employed distinct testing-for-isolation methods for symptomatic and asymptomatic cases, with voluntary testing-for-isolation for symptomatic infections and surveillance testing-for-isolation for asymptomatic ones. It becomes necessary to explore the effect of the symptomatic ratio θ on the counterfactual analysis of the reconstruction. Public health data indicates an average symptomatic ratio of $124.0M/144.6M = 0.85$ from February 2020 to September 2021 in the United

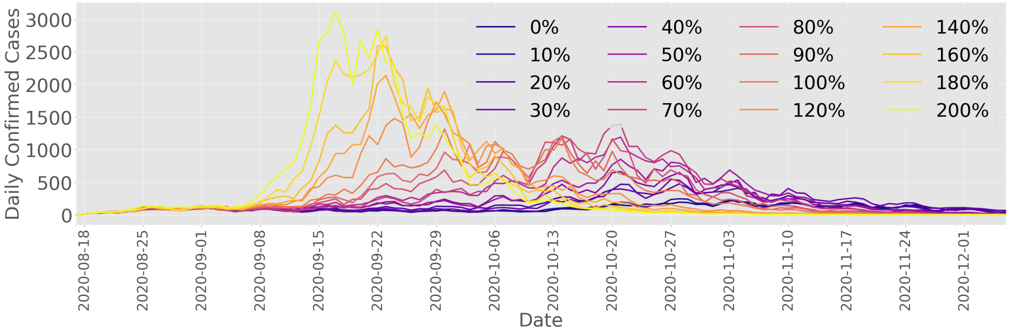

Fig S26. Daily confirmed cases of the hypothetical outbreak without isolation at the UIUC campus, assuming different proportions follow the isolation rules from the real-world outbreak. The more confirmed cases that follow the isolation strategy upon testing positive during Fall 2020 and Spring 2021, the less severe the hypothetical spread would be without the testing-for-isolation strategy.

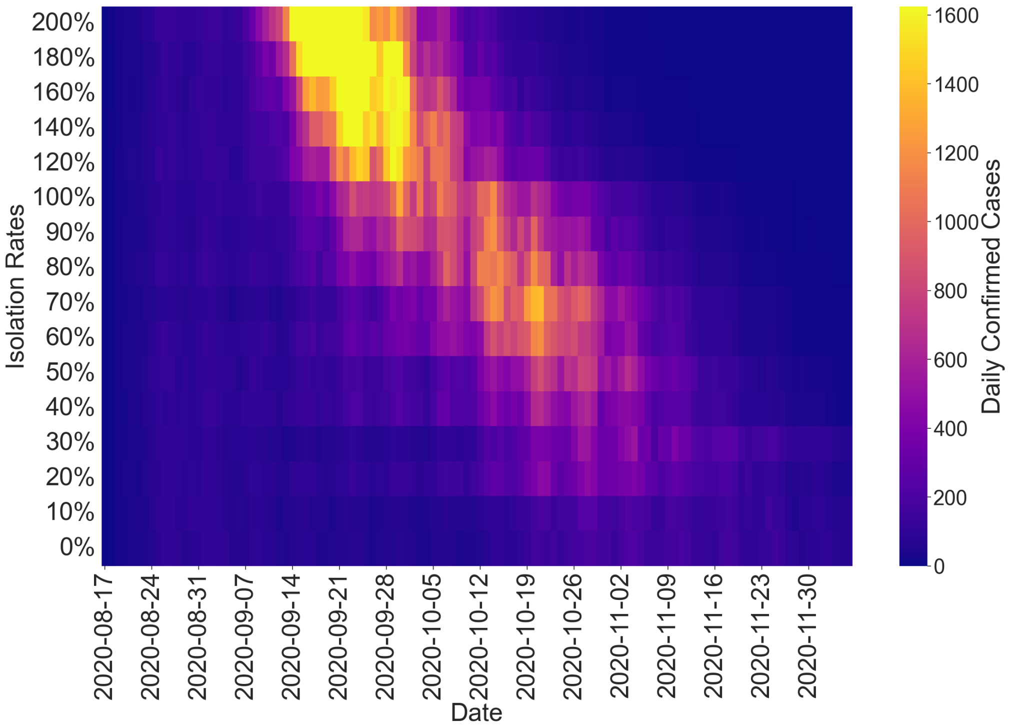

Fig S27. Daily confirmed cases of the hypothetical outbreak without isolation at the UIUC campus, assuming different proportions follow the isolation rules from the real-world outbreak. The higher the assumed isolation rate implemented by the university, the higher the peak infection value would be during the hypothetical spreading scenario without the isolation strategy, and the earlier the outbreak would occur in calendar time.

States [36]. Purdue considered that cases caught by voluntary testing as symptomatic infection, and cases caught by surveillance testing as asymptomatic infection. Due to the fact that not all symptomatic cases were tested voluntarily, some symptomatic cases entered the testing pool of the surveillance testing. Hence, Purdue recorded a different symptomatic ratio (55%) compared to the record from the Centers for Disease Control

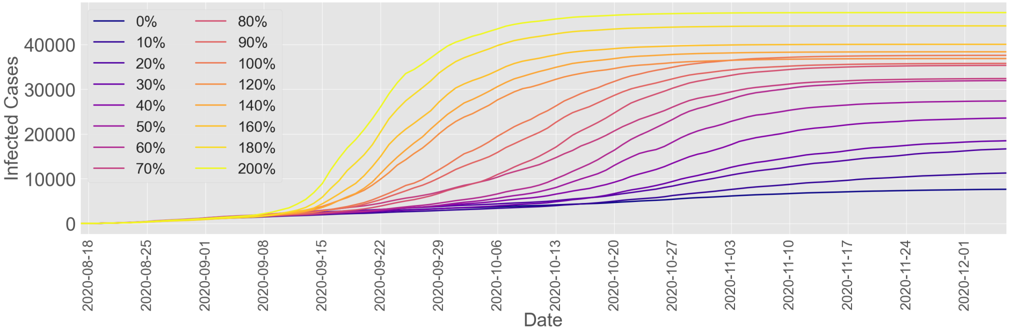

Fig S28. Confirmed cumulative cases of the hypothetical outbreak without isolation at the UIUC campus, assuming different proportions follow the isolation rules from the real-world outbreak. When $\alpha_I = 200\%/7$ (where all confirmed cases in the real-world outbreak follow the isolation rule), the hypothetical spreading scenario generates the worst-case scenario seen in S22 Fig in SI.

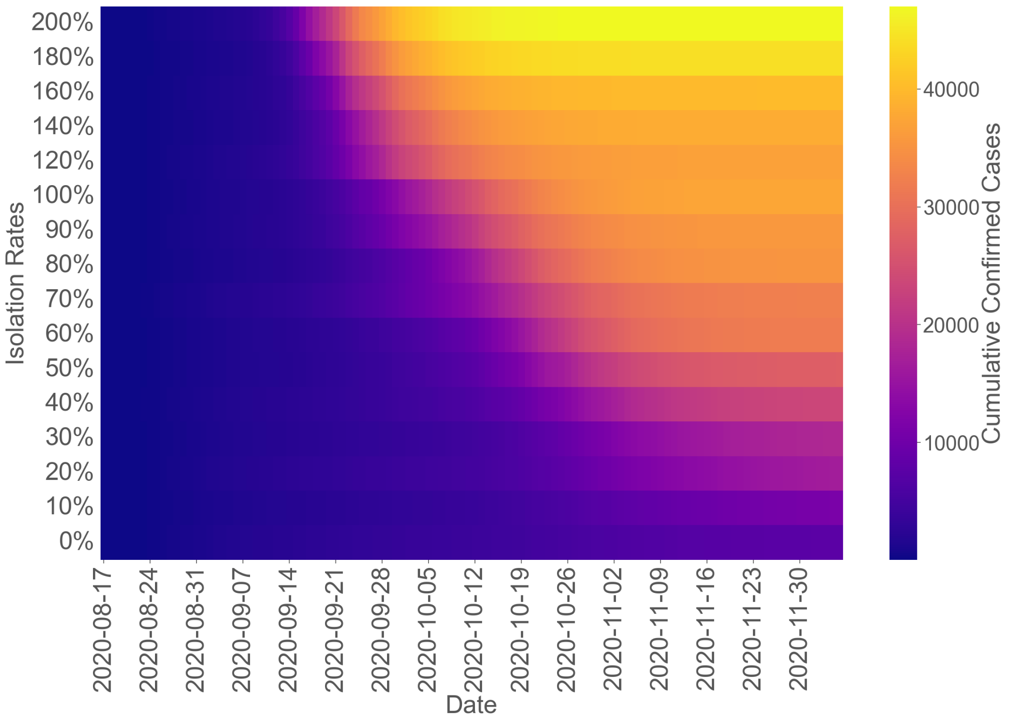

Fig S29. Confirmed cumulative cases of the hypothetical outbreak without isolation at the UIUC campus, assuming different proportions follow the isolation rules from the real-world outbreak.

and Prevention. To match Purdue's record, we also consider the positive cases that were caught by the voluntary testing at Purdue as the symptomatic cases. We further consider cases caught through voluntary testing are isolated within a week of testing positive (since they are cautious and willing to be tested), spread evenly over the week. To assess the impact of the symptomatic ratio on the epidemic reconstruction of the hypothetical spread, under Purdue's implemented testing-for-isolation strategy, we adjusted θ within the range of $\{10\%, 30\%, 50\%, 70\%, 90\%\}$.

In this scenario, we vary the ratio of the symptomatic infection while keeping the same weekly isolation rate of 30% for asymptomatic cases under surveillance testing,

similar to what was done when estimating the effective reproduction number and reconstructing the hypothetical spreading process without any isolation at Purdue in Section SI-2-E-2. Hence, based on Eq (17), the scaling factor is given by

$$\mathcal{F}(\alpha_P) = \frac{\mathcal{R}(\alpha_P)}{\mathcal{R}} = \frac{(\theta \sum_{i=1}^{n} \underline{v}_i(\underline{\alpha}_P) + (1-\theta) \sum_{i=1}^{n} \overline{v}_i(\overline{\alpha}_P))}{\mathcal{R}}, \tag{19}$$

where α_P is the overall isolation rate from the isolation rates under voluntary testing-for-isolation ($\underline{\alpha}_P = 1/7$) and surveillance testing-for-isolation ($\overline{\alpha}_P = 0.3/7$), respectively. According to Eq (19), a higher symptomatic ratio (θ) results in a smaller scaling factor $\mathcal{F}(\alpha_P)$, which leads to a higher reverse engineered effective reproduction number (Eq (18)).

We explore various ratios for symptomatic infections (θ) from the set $\{10\%, 30\%, \ldots, 90\%\}$. The daily confirmed cases of the reconstructed hypothetical outbreak, under the assumption of different symptomatic infection ratios (θ), at the Purdue campus are illustrated in S30 Fig in SI, along with the corresponding heatmap in S31 Fig in SI. Additionally, the cumulative confirmed cases under various symptomatic infection ratios at the Purdue campus are shown in S32 Fig in SI, with the corresponding heatmap in S33 Fig in SI. S30 and S31 Figs in SI demonstrate projections based on the scenario where Purdue's surveillance testing-for-isolation strategy isolated 30% of asymptomatic cases weekly. The hypothetical outbreaks appear to be more severe when lower proportions of symptomatic infections are considered. This result is reflected in the higher peak infection values and cumulative confirmed cases.

Our analysis assumes that all symptomatic infections were subjected to the testing-for-isolation strategy, tested, and isolated through voluntary testing. Meanwhile, $\overline{\alpha}_P = 30\%/7$ of asymptomatic infections were tested and isolated through surveillance testing. Therefore, the severity of the hypothetical outbreaks shown in S8 Fig in SI depends on the proportion of confirmed cases that are symptomatic or asymptomatic. For instance, a higher proportion of confirmed symptomatic cases (captured by the $\theta = 90\%$ line in S30 Fig in SI) resulted in milder hypothetical outbreaks compared to scenarios with a higher proportion of asymptomatic cases (as depicted by the $\theta = 10\%$ line in S30 Fig in SI).

After discussing the impact of the symptomatic ratio θ on the epidemic reconstruction, similar to UIUC, we consider the impact of the isolation rate on asymptomatic infections. We leverage the analysis to show why we chose 30% for our weekly isolation rate of asymptomatic infections. In this scenario, we vary the effectiveness of the testing-for-isolation strategy Purdue implemented on asymptomatic infections. The daily isolation rate of asymptomatic infections is drawn from the set $\overline{\alpha}_P \in \{10\%/7, 20\%/7, \ldots, 100\%/7\}$. We keep the ratio of symptomatic infections as $\theta = 55\%$, the same as when estimating the effective reproduction number. Hence, based on Eq (19), the scaling factor is given by

$$\mathcal{F}(\alpha_P) = \frac{\mathcal{R}(\alpha_P)}{\mathcal{R}} = \frac{(0.55 \sum_{i=1}^{n} \underline{v}_i(\underline{\alpha}_P) + (1-0.55) \sum_{i=1}^{n} \overline{v}_i(\overline{\alpha}_P))}{\mathcal{R}}, \tag{20}$$

where $\alpha_P = 0.55\underline{\alpha}_P + 0.45\overline{\alpha}_P$ is the overall isolation rate from the isolation rates under voluntary testing-for-isolation strategy ($\underline{\alpha}_P = 1/7$) and surveillance testing-for-isolation strategy ($\overline{\alpha}_P \in \{10\%/7, 20\%/7, \ldots, 100\%/7\}$), respectively. The daily confirmed cases of the reconstructed hypothetical spreading scenarios under different assumed isolation rates on asymptomatic cases at the Purdue campus in the real-world outbreak, are illustrated in S34 Fig in SI, along with the corresponding heatmap in S35 Fig in SI. Additionally, the cumulative confirmed cases under various isolation rates on asymptomatic cases at the Purdue campus are shown in S36 Fig in SI, with the corresponding heatmap in S37 Fig in SI.

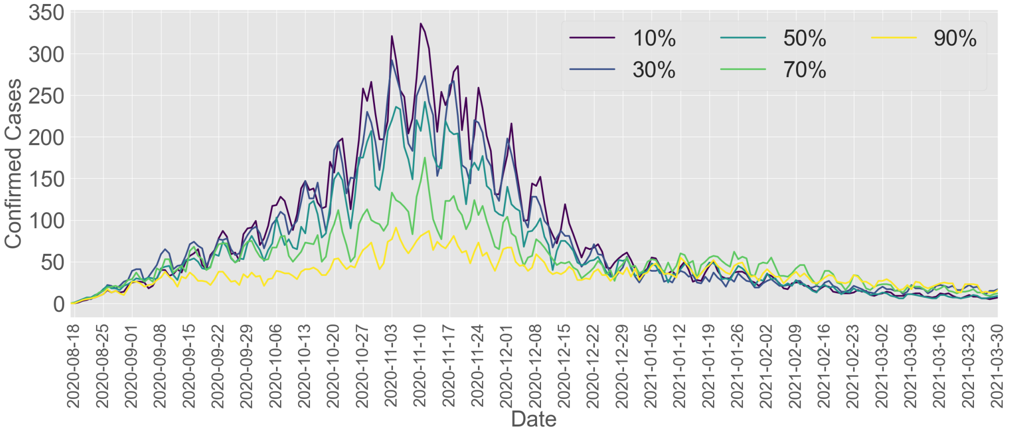

Fig S30. Daily confirmed cases of the hypothetical outbreak with $\underline{\alpha}_P = 1/7$ and $\overline{\alpha}_P = 0.3/7$ at the Purdue campus, assuming different proportions of symptomatic cases during the real-world outbreak. The hypothetical outbreaks appear to be more severe when lower proportions of symptomatic infections are considered, since fewer infected cases (i.e., symptomatic cases) will be caught and isolated based on the voluntary testing-for-isolation strategy.

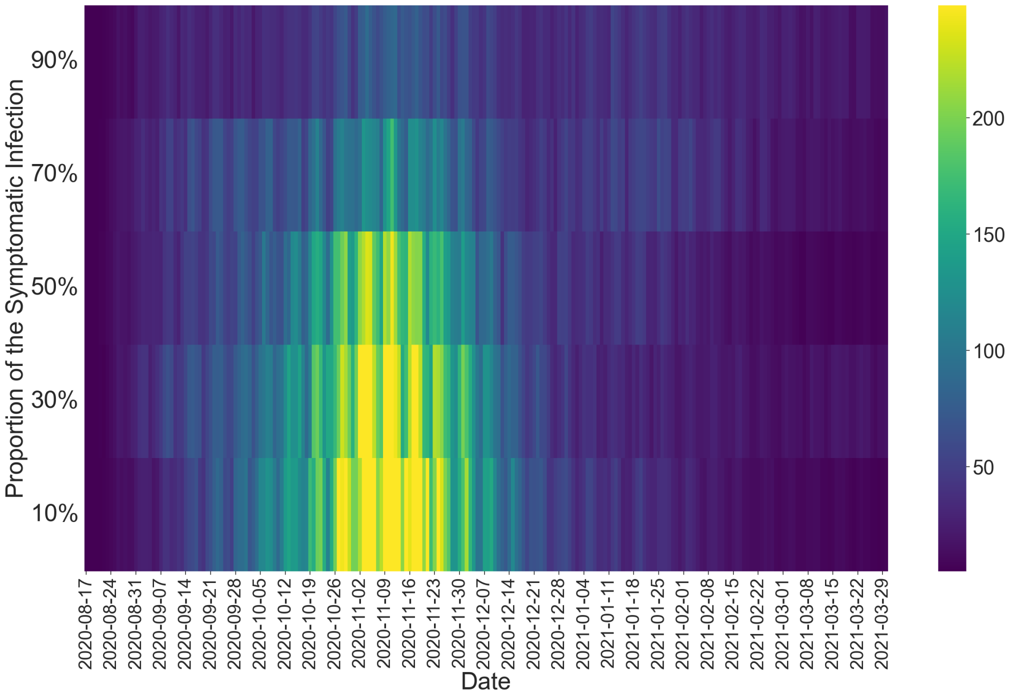

Fig S31. Daily confirmed cases of the hypothetical outbreak with $\underline{\alpha}_P = 1/7$ and $\overline{\alpha}_P = 0.3/7$ at the Purdue campus, assuming different proportions of symptomatic cases during the real-world outbreak. The outbreaks appear to be less severe when higher proportions of symptomatic infections are considered, which is intuitive since the voluntary testing-for-isolation strategy for symptomatic cases dominates the testing-for-isolation strategy at Purdue. Without the surveillance testing-for-isolation strategy for asymptomatic cases, a higher proportion of symptomatic infections would result in a higher proportion of infected cases being caught and isolated.

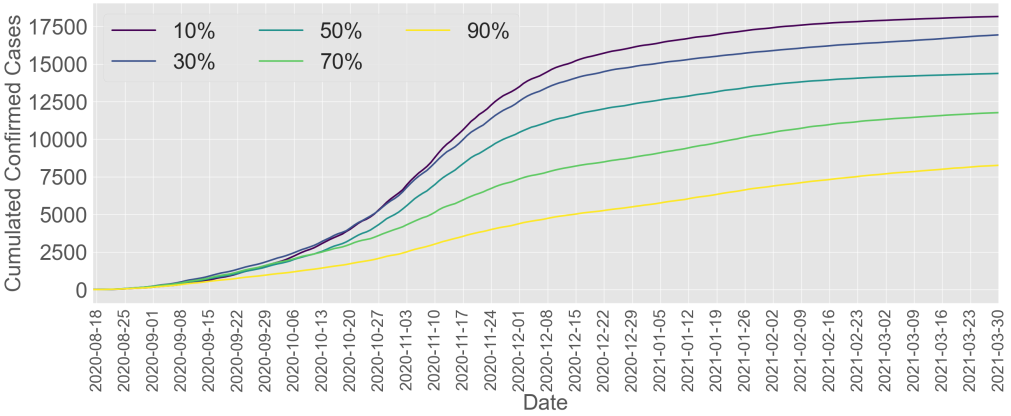

Fig S32. Cumulative confirmed cases of the hypothetical outbreak with $\underline{\alpha}_P = 1/7$ and $\overline{\alpha}_P = 0.3/7$ at the Purdue campus, assuming different proportions of symptomatic cases during the real-world outbreak. Unlike the isolation rate, the ratio of symptomatic cases will not delay the outbreak. A higher proportion of confirmed symptomatic cases (captured by $\theta = 90\%$) results in milder outbreaks compared to scenarios with a higher proportion of asymptomatic cases (captured by $\theta = 10\%$).

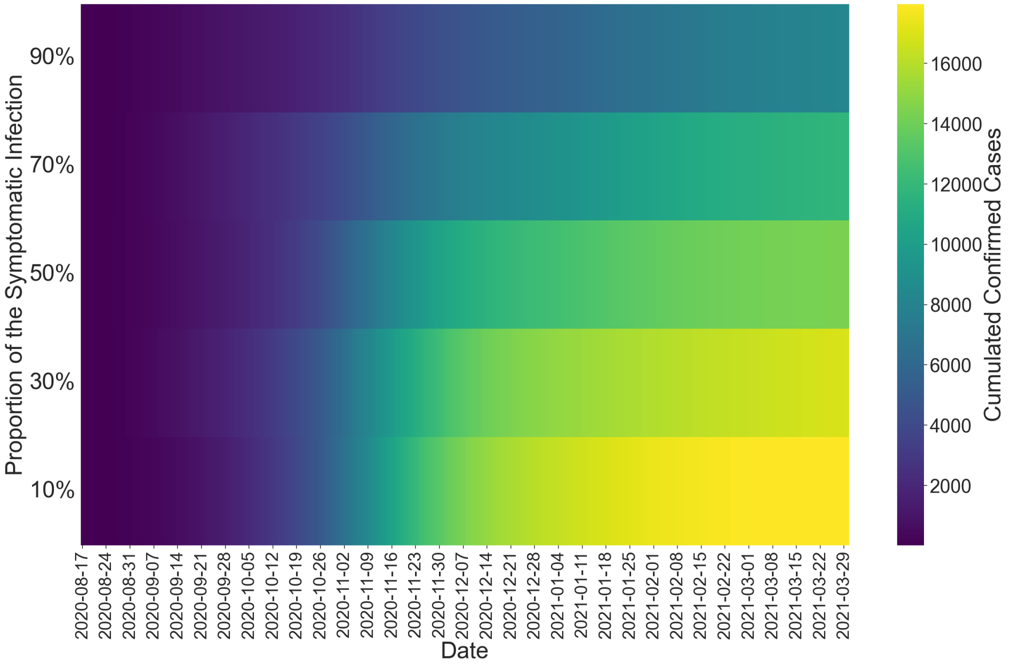

Fig S33. Cumulative confirmed cases of the hypothetical outbreak with $\underline{\alpha}_P = 1/7$ and $\overline{\alpha}_P = 0.3/7$ at the Purdue campus, assuming different proportions of symptomatic cases during the real-world outbreak.

S34 and S35 Figs in SI demonstrate projections based on the scenario where Purdue's surveillance testing-for-isolation strategy isolated $\overline{\alpha}_P$ of asymptomatic infection daily. The hypothetical outbreaks appear to be more severe when a higher ratio of asymptomatic infections is isolated. This result is reflected in the higher peak infection values and cumulative confirmed cases. Our analysis assumes that all symptomatic infections were subjected to the testing-for-isolation strategy, tested, and isolated through voluntary testing, with the ratio of symptomatic infections being $\theta = 0.55$.

Meanwhile, $\overline{\alpha}_P \in \{10\%/7, \ldots, 100\%/7\}$ of asymptomatic infections were tested and isolated through surveillance testing daily. S34 Fig in SI and S36 Fig in SI correlate with the condition that a higher implemented daily isolation rate $\overline{\alpha}_P$ by Purdue generates a lower scaling factor in Eq (20), leading to more severe outbreaks when the surveillance testing-for-isolation strategy is not in place during our reconstructed hypothetical spreading scenarios. Further, as illustrated by S36 and S37 Figs in SI, when $\overline{\alpha}_P$ is higher than the isolation rate from the set $\{10\%/7, 20\%/7, 30\%/7, 40\%/7\}$, the cumulative infected cases are similar, which are also much lower than the situation where $\overline{\alpha}_P \geq 50\%/7$. Therefore, we select $\overline{\alpha}_P = 30\%/7$ in the analysis to consider that the implemented surveillance testing-for-isolation at Purdue did play an important role in pandemic mitigation, but we avoid exaggerating the effectiveness of the surveillance testing-for-isolation since 1) the voluntary testing at Purdue was also critical and 2) it was hard to trace most of the asymptomatic infected cases (i.e., large $\overline{\alpha}_P$).

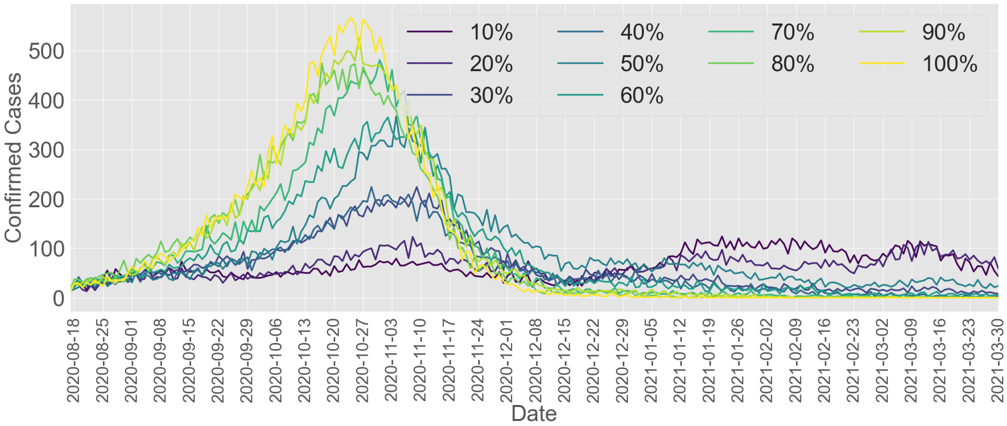

Fig S34. Daily confirmed cases of the hypothetical spreading scenarios over the Purdue campus, assuming different implemented isolation rates ($\overline{\alpha}_P$) for asymptomatic cases. Given that a higher ratio of asymptomatic infections was isolated in reality, the outbreaks in the hypothetical spreading scenario without the surveillance testing-for-isolation strategy would appear more severe. This observation is reflected in the higher peak infection values.

In summary, considering the testing-for-isolation strategies implemented by UIUC and Purdue, which aim to manage infected cases and prevent significant infection spikes (as shown in S3 and S8 Figs in SI), we conclude that without these strategies, the epidemic would have been worse. It is critical to emphasize again that the impact of the testing-for-isolation strategy is dependent on the isolation rate. Solely conducting testing without encouraging isolation would make little difference, as testing can only detect infectious cases. Fortunately, the isolation rate is typically proportional to the testing rate due to other policies and shifts in behavior resulting from heightened awareness. Furthermore, the reconstruction of the hypothetical outbreaks is affected by population behavior, the symptomatic ratio, and various other factors influencing the counterfactual analysis. We also assume that the change in the strength of the intervention strategy does not further impact students' behavior, viral loads, or other factors. Therefore, obtaining precise information about the spread is critical to successfully reverse engineering the effective reproduction number. While the analytical results are specific to particular conditions and assumptions, as well as their corresponding reconstructions, the methods proposed for reconstruction and intervention evaluation are general. These methods offer a new perspective for researchers to assess the impact of implemented pandemic intervention strategies by

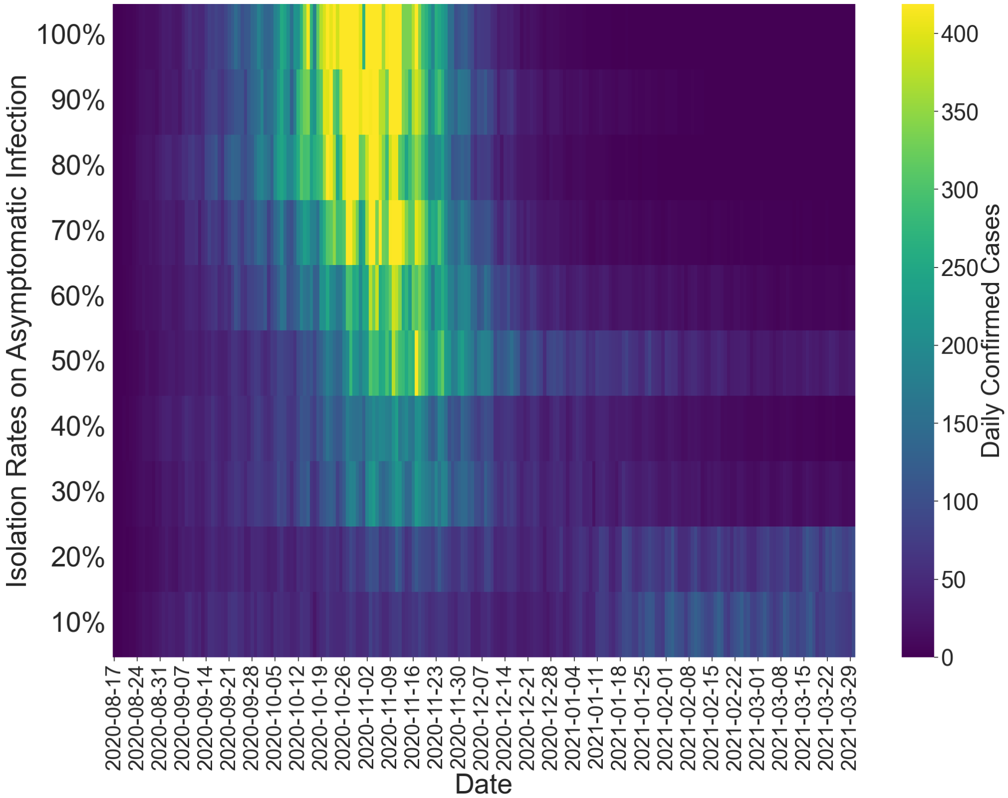

Fig S35. Daily confirmed cases of the hypothetical spreading scenarios over the Purdue campus, assuming different implemented isolation rates ($\overline{\alpha}_P$) for asymptomatic cases.

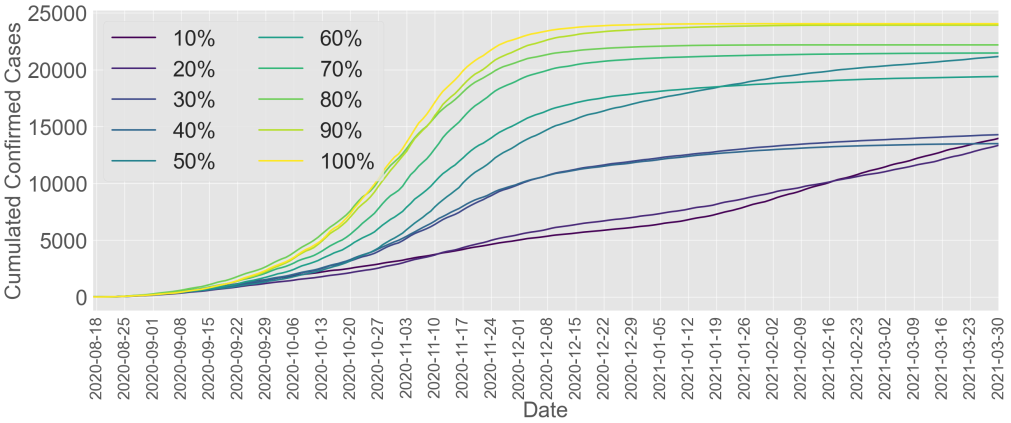

Fig S36. Cumulative confirmed cases of the hypothetical spreading scenarios over the Purdue campus, assuming different implemented isolation rates ($\overline{\alpha}_P$) for asymptomatic cases. Cumulative confirmed cases also reflect that the outbreaks appear to be more severe when a higher ratio of asymptomatic infections in reality is isolated.

reverse engineering the effective reproduction number in a novel manner.

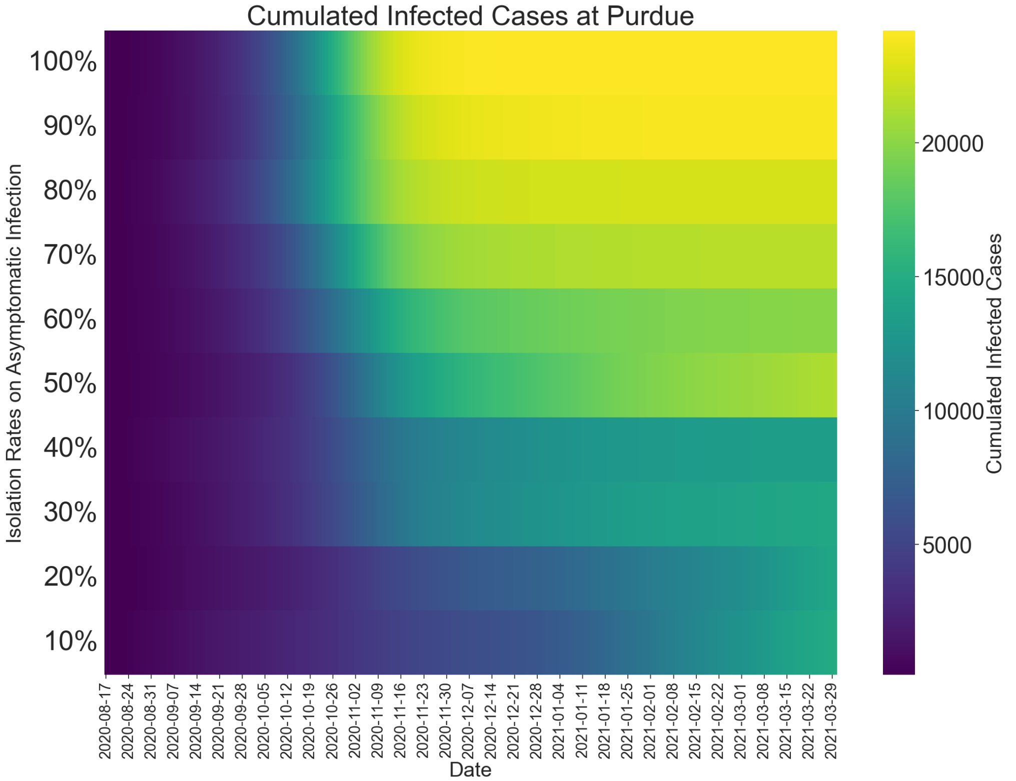

Fig S37. Cumulative confirmed cases of the hypothetical spreading scenarios over the Purdue campus, assuming different implemented isolation rates ($\overline{\alpha}_P$) for asymptomatic cases.

SI-2-F. The Impact of the Isolation Rate on Spreading Processes

In this section, we extend the method of reverse engineering the effective reproduction number in Eq (18) to the scenario where an alternative strength of the isolation rate is implemented in the hypothetical spreading scenario, in order to explore the impact of isolation rates on epidemic spread. We first focus on a fixed isolation rate, often referred to as the open-loop control strategy. Our analysis involves the reconstructed hypothetical spreading scenarios over both campuses, creating an experimental environment to examine potential outcomes if universities had implemented varying isolation rates during Fall 2020.

As shown in SI-2-B and SI-2-E-1, we introduce a method for generating confirmed cases using real-world data from S3 and S8 Figs in SI. The core method incorporates utilizing the scaling factor to reverse engineer the effective reproduction number, as defined in Eq (17). Generally, accounting for the implemented daily isolation rate in reality as α, we denote the scaling factor as $\mathcal{F}(\alpha)$ according to Eq (12). Given the estimated effective reproduction number under the implemented isolation rate α, denoted as $\mathcal{R}_t(\alpha)$ from Eq (17), we can approximate the basic reproduction number without the isolation rate α, denoted as $\frac{\mathcal{R}_t(\alpha)S(t)}{\mathcal{F}(\alpha)N}$. We use $S(t)$ to denote the susceptible population at time t and N to denote the total fixed population. When introducing a different isolation rate α^* at time t in the hypothetical spreading scenario, we denote the new scaling factor as $\mathcal{F}(\alpha^*)$. We compute the effective reproduction number of the

hypothetical outbreak with the alternative isolation rate, changing from α to α^*:

$$\mathcal{R}_t(\alpha^*) = \mathcal{R}_t(\alpha) \frac{S^*(t)}{S(t)} \frac{\mathcal{F}(\alpha^*)}{\mathcal{F}(\alpha)}. \tag{21}$$

The detailed derivation of the equation is given in Eq (14) of the main manuscript.

Using Eq (21), we are able to calculate the effective reproduction number $\mathcal{R}_t(\alpha^*)$ of the hypothetical outbreak under a different fixed isolation rate α^* by utilizing the estimated effective reproduction number $\mathcal{R}_t(\alpha)$ under the implemented isolation rate α in reality (e.g., S16 Fig in SI). The computed scaling factors are obtained by scaling the default serial interval distribution w with the isolation rates α and α^* as described in Eq (13) and Eq (17), and the ratio between the susceptible population at any given time t. Further, with Eq (21) we generate the effective reproduction number $\mathcal{R}_t(\alpha^*)$ of the hypothetical outbreak under a different fixed isolation rate α^*. By applying Eq (13), we use $w(\alpha^*)$ to calculate the infection profile $v(\alpha^*)$ under the fixed isolation rate α^*. The isolation rate is not affected by the severity of the epidemic spread, making it an open-loop control strategy. As discussed in SI-2-E-2, we assume that the change in the strength of the intervention strategy does not further impact students' behavior, viral loads, or other factors. Additionally, we discuss a method to obtain the susceptible populations for both the real-world and hypothetical spreads in SI-2-E-2, where the susceptible population can be obtained by subtracting the daily infected cases from the total fixed population. Therefore, utilizing the synthetic data generation mechanism outlined in Section SI-2-B, we generate confirmed cases of the hypothetical outbreaks under different fixed isolation rates.

In the previous section, we conclude that the reconstructed hypothetical spreading environment is sensitive to various factors. In this section, we utilize the worst-case reconstructed hypothetical spreading scenario at the UIUC campus (S22 Fig in SI) as our testing environment. The worst-case scenario considers that a case caught via testing will not be isolated, and the case will behave as uninfected until recovery. We compare the hypothetical outcomes under different fixed isolation rates (open-loop control strategy) on the UIUC campus during Fall 2020, considering isolation rates that are less than or equal to 200% weekly. Under the fact that the testing rate at UIUC during Fall 2020 is around 200% weekly, the weekly isolation rates $\alpha_P \times 7$ are drawn from the set $\{0\%, 10\%, 20\%, \ldots, 90\%\}$ and $\{100\%, 120\%, \ldots 180\%, 200\%\}$. Additionally, the testing process does not differentiate between symptomatic and asymptomatic cases, since we leverage the same infection profile and population behavior for all infected cases.

Based on the daily confirmed cases presented in S38 Fig in SI, we observe that higher isolation rates generate relatively smoother and flatter curves in terms of confirmed cases in the hypothetical spreading scenarios. The bright area in S39 Fig in SI indicates that higher isolation rates result in lower and delayed spikes. Examining the cumulative confirmed cases in S40 Fig in SI, we conclude that higher isolation rates generally lead to a lower number of total cumulative cases. An important distinction was observed between isolation rates below 100% per week and isolation rates above 100% per week. When the isolation rate was 100% or lower, the cumulative confirmed cases amounted to approximately 35,000 out of a total population of 50,000. The primary difference between isolating at 100% and isolating at 0% per week was that isolating at 100% per week flattened the curve more and resulted in lower peak infection values.

S41 Fig in SI shows that when the isolation rate exceeds 100% per week, increasing the rate by 20% per week notably decreases the total cumulative cases. This phenomenon can be explained by considering the threshold conditions of an epidemic spreading process. Epidemics often exhibit threshold conditions that determine their spreading behavior. Thus, for the hypothetical spreading processes on the UIUC

campus, when the isolation rate was below 100% per week, significant outbreaks were inevitable, as they approached an equilibrium where nearly everyone would eventually be infected. We conclude that, in order to effectively mitigate the hypothetical spreading process over the UIUC campus under the worst-case scenario situation, it is necessary to test everyone at least once a week.

When the weekly isolation rate reaches 200%, the daily confirmed cases and cumulative confirmed cases match the UIUC confirmed cases introduced in S20 Fig in SI. Similarly, when the isolation rate was zero, the daily confirmed cases and cumulative confirmed cases aligned with the reconstruction results in S22 Fig in SI. This phenomenon demonstrates the idea of reverse engineering the effective reproduction number to study the hypothetical outbreak under alternative intensities of the isolation rate. Therefore, under the conditions that we leverage for reconstructing the hypothetical spread at the UIUC campus, we conclude that in order to avoid major outbreaks in the worst-case scenario at the UIUC campus, it is necessary to test the entire campus once a week, then ensure that all confirmed cases are isolated. Increasing the isolation rate beyond that can further flatten the curve and reduce the total number of confirmed cases. All these analyses are based on the worst-case scenario assumption at UIUC, shown in S22 Fig in SI. If we test these fixed isolation rates in a different testing environment at UIUC, such as the other reconstructed hypothetical spreading environments provided in S26 Fig in SI, we obtain significantly different results. Hence, when evaluating epidemic mitigation strategies like isolation rates, it is critical to consider factors such as population behavior and virus spreading behavior.

It is important to note the differences between S26 Fig in SI and S38 Fig in SI. Both figures illustrate a counterfactual analysis of hypothetical spreading scenarios at the UIUC campus with varying isolation rates. In the counterfactual analysis of SI-2-E-3, we assume that different isolation rates are implemented in the real-world outbreak at UIUC, while the reconstructed hypothetical spreading scenario sets this implemented isolation rate to zero. For the counterfactual analysis in this section, we assume that the fixed 200% weekly isolation rate is implemented in the real-world outbreak at UIUC, while the reconstructed hypothetical spreading scenario changes this isolation rate from 200% to an alternative isolation rate. Thus, S26 Fig in SI provides multiple scenarios that simulate the hypothetical outbreaks at UIUC without their implemented testing-for-isolation strategy. S38 Fig in SI tests the impact of different isolation strategies on one reconstructed hypothetical spreading scenario from SI-2-E-2, which is the worst-case scenario captured by S22.

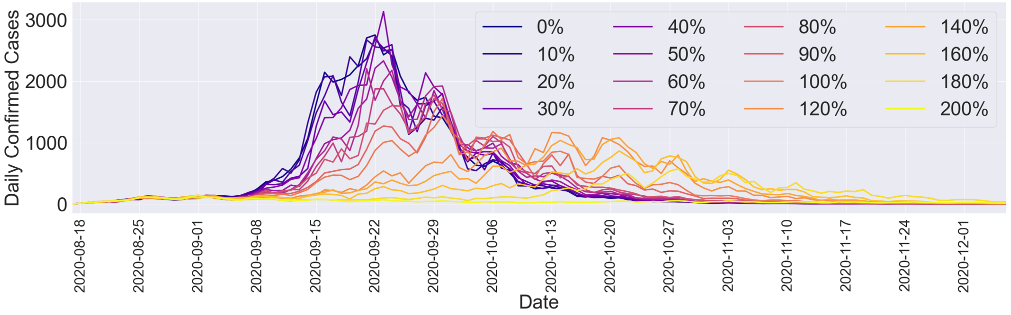

Fig S38. Daily confirmed cases of the hypothetical spreading scenarios over the UIUC campus with different isolation rates. Assuming that the implemented weekly isolation rate is given by $\alpha_P \times 7 = 200\%$ in reality by UIUC, higher weekly isolation rates in the hypothetical spreading scenarios generate relatively smoother and flatter curves in terms of daily confirmed cases.

Compared to UIUC, we focus on evaluating the impact of different isolation rates on

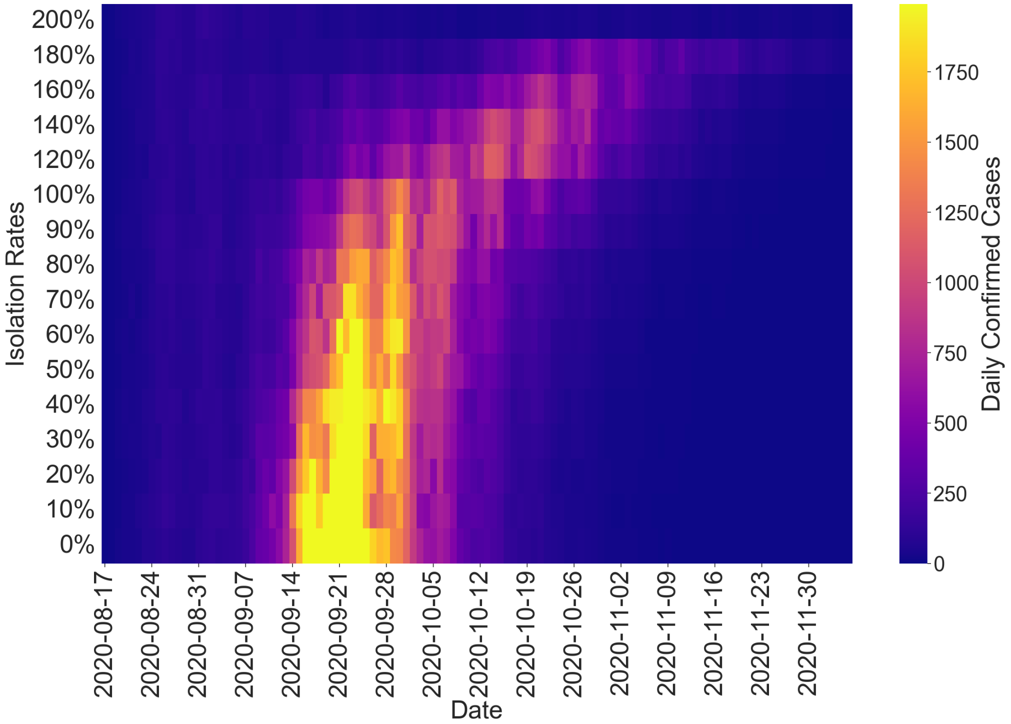

Fig S39. Daily confirmed cases of the hypothetical spreading scenarios over the UIUC campus with different isolation rates. Assuming that the implemented weekly isolation rate is given by $\alpha_P \times 7 = 200\%$ in reality by UIUC, higher weekly isolation rates in the hypothetical spreading scenarios result in lower and delayed spikes.

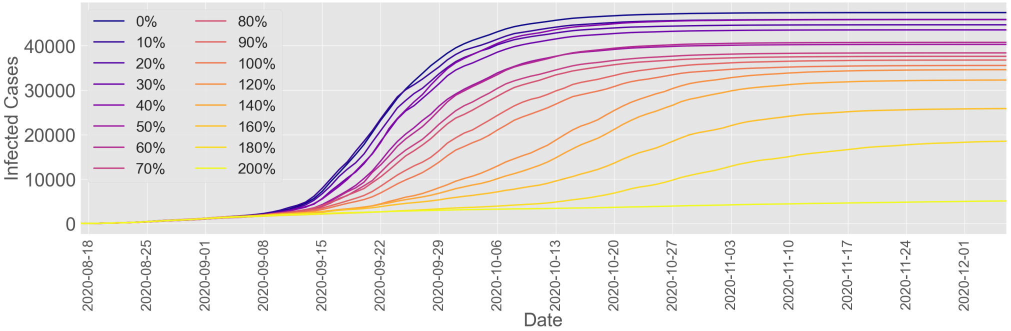

Fig S40. Cumulative confirmed cases of the hypothetical spreading scenarios over the UIUC campus with different isolation rates. Assuming that the implemented weekly isolation rate is given by $\alpha_P \times 7 = 200\%$ in reality by UIUC, higher weekly isolation rates in the hypothetical spreading scenarios generally lead to a lower number of total cumulative cases.

the spreading process over the Purdue campus, considering a different setting:

- Symptomatic and asymptomatic cases have the same spreading behavior.
- Symptomatic cases on the Purdue campus seek testing-for-isolation within a week after becoming infectious, equivalent to a 100% weekly testing and isolation rate for symptomatic cases.
- The isolation rate for asymptomatic cases is 30% per week.

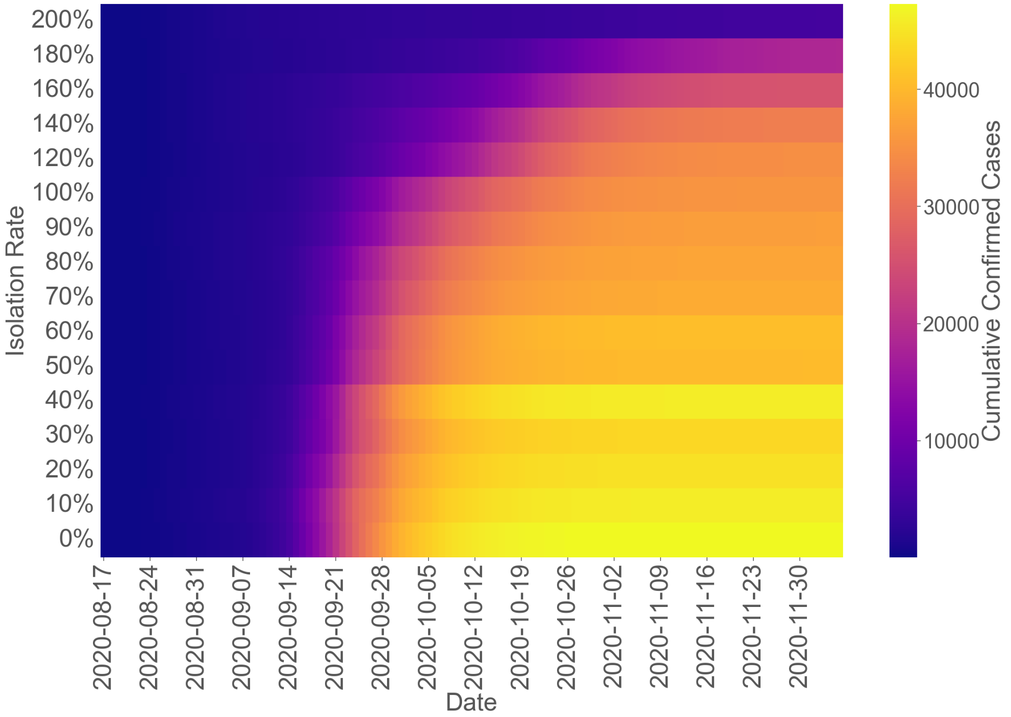

Fig S41. Cumulative confirmed cases of the hypothetical spreading scenarios over the UIUC campus with different isolation rates. Assuming that the implemented weekly isolation rate is given by $\alpha_P \times 7 = 200\%$ in reality by UIUC, when the isolation rate in the hypothetical spreading scenarios exceeds 100% per week, increasing the rate by 20% per week notably decreases the total cumulative cases.

- Based on our discussion in SI-2-D-3 and SI-2-D-4, 55% of the population on the Purdue campus is assumed to be symptomatic.

To capture the reconstructed hypothetical spreading environment without the implemented isolation under surveillance testing, we refer to S24 Fig in SI with the $\overline{\alpha}_P = 30\%/7$ daily isolation rate scenario. We consider a milder spread over Purdue in order to evaluate the impact of the surveillance testing under a different spread behavior. We expect that the spreading process with different isolation rates under surveillance testing will be less severe compared to UIUC. Similarly to UIUC, we consider weekly isolation rates under surveillance testing for asymptomatic cases from the sets $\{0\%, 10\%, 20\%, \dots, 90\%\}$, $\{100\%, 120\%, \dots 180\%, 200\%\}$. Utilizing the method for reverse engineering the effective reproduction number proposed in Eq (21) and the simulation method in Section SI-2-B, we generate the daily confirmed cases of the hypothetical outbreaks under alternative isolation rates for asymptomatic cases, in S42 Fig in SI and the corresponding heatmap in S43 Fig in SI. As expected, the daily confirmed cases of the hypothetical outbreaks at the Purdue campus are notably lower than those of the hypothetical outbreaks at UIUC. Higher isolation rates under the surveillance testing result in smoother and flatter curves in terms of confirmed cases, as observed in S42 Fig in SI. Moreover, S43 Fig in SI demonstrates that higher isolation rates also lead to lower spikes.

We further plot the cumulative confirmed cases of the hypothetical outbreaks in S44 Fig in SI with the corresponding heatmap in S45 Fig in SI. The cumulative confirmed cases in S44 Fig in SI suggest that higher isolation rates generally lead to fewer cumulative confirmed cases. Analyzing the heatmaps in S43 and S45 Figs in SI, a

marked difference is observed between implementing isolation rates in the hypothetical outbreaks for asymptomatic cases below 50% per week and rates above 50% per week. When the weekly isolation rate under surveillance testing for asymptomatic cases is set at 50% or lower per week, a notable outbreak occurs with large spikes towards the end of the Fall 2020 semester at Purdue. During this period, the cumulative confirmed cases exceed $5,000$. However, when the isolation rate for asymptomatic cases surpasses 50% per week, no significant outbreak is observed throughout the Fall 2020 semester at Purdue. Furthermore, increasing the weekly isolation rate does not substantially decrease the total number of cumulative cases. In the reconstructed hypothetical spreading process over the Purdue campus, if the isolation rate for asymptomatic cases remains below 50% per week, the cumulative confirmed cases range from $5,000$ to $10,000$ during Fall 2020. Yet, when the rate exceeds 50%, an increase in the isolation rate leads to a larger decrease in the cumulative confirmed cases. In this situation, the cumulative confirmed cases range from 500 to $2,500$ during Fall 2020. This pattern could be explained by considering the potential threshold conditions of the epidemic spreading process over the Purdue campus. It is hypothesized that an isolation rate under the surveillance testing rate of 50% serves as the threshold condition, determining whether there will be an outbreak at the end of the Fall 2020 semester. The implemented surveillance testing-for-isolation by Purdue, which caught approximately 30% of asymptomatic cases (as discussed in S17 Fig in SI and reconstructed in S21 Fig in SI), is lower than the 50% weekly isolation rate threshold condition. Hence, based on the real-world confirmed cases at Purdue in S8 Fig in SI, an outbreak is observed in the middle and at the end of the Fall 2020 semester. From the analysis in this example, it can be inferred that testing and then isolating more than 50% of asymptomatic cases per week during Fall 2020 at Purdue could have helped avoid the significant spikes observed on campus.

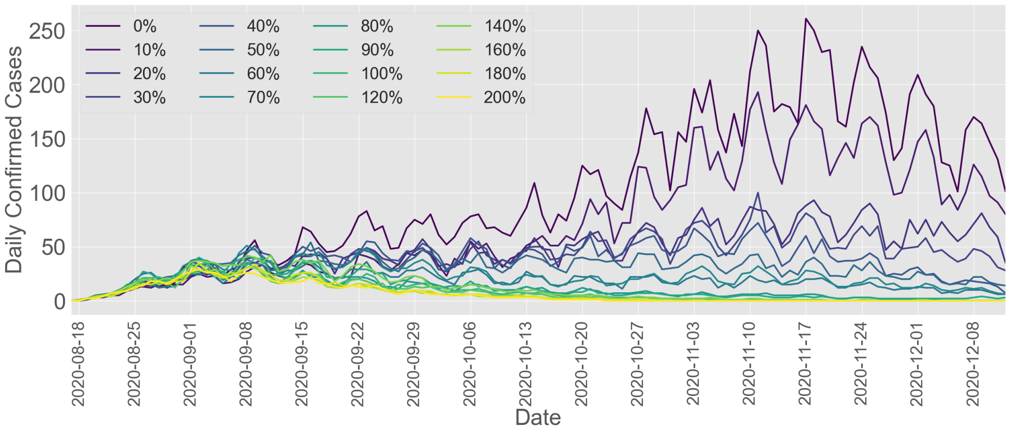

Fig S42. Daily confirmed cases of the hypothetical outbreaks over the Purdue campus with alternative isolation rates under surveillance testing. The daily confirmed cases of the hypothetical outbreak with alternative weekly isolation rates for asymptomatic infections at the Purdue campus are notably lower than those at UIUC due to the existence of voluntary testing-for-isolation. Higher isolation rates under surveillance testing result in smoother and flatter curves in terms of confirmed cases.

This section employs the spread data from the UIUC and Purdue campuses to validate the reverse engineering of the effective reproduction number for evaluating the implemented mitigation strategies in the real-world outbreaks. The goal is to conduct counterfactual analysis to refine outcomes by adjusting the strength of these strategies. Examining the potential outcomes at both UIUC and Purdue under varying fixed

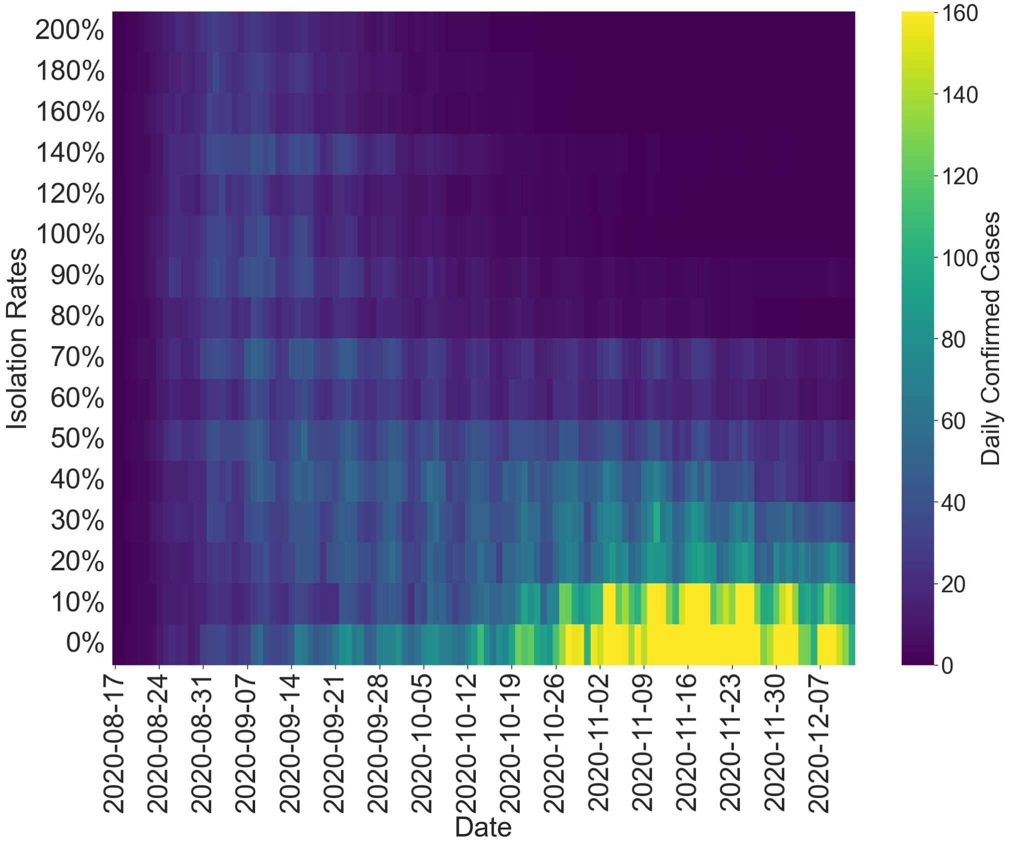

Fig S43. Daily confirmed cases of the hypothetical outbreaks over the Purdue campus with alternative weekly isolation rates under surveillance testing. Higher isolation rates under surveillance testing result in smoother and flatter curves in terms of confirmed cases.

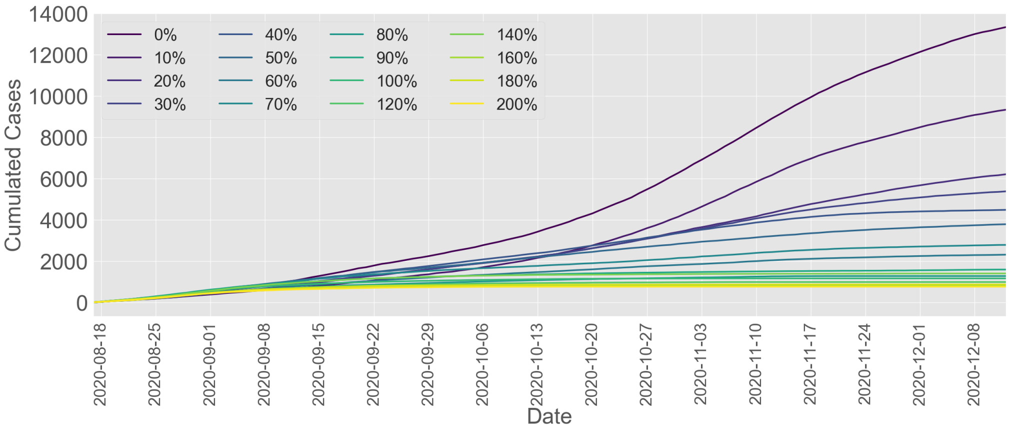

Fig S44. Cumulative confirmed cases of the hypothetical outbreaks over the Purdue campus with alternative weekly isolation rates under surveillance testing. The cumulative confirmed cases suggest that higher isolation rates in the hypothetical outbreaks generally lead to fewer cumulative confirmed cases.

isolation rates reveals threshold conditions associated with isolation rates, which might have prevented possible outbreaks. Computational results are contingent on the

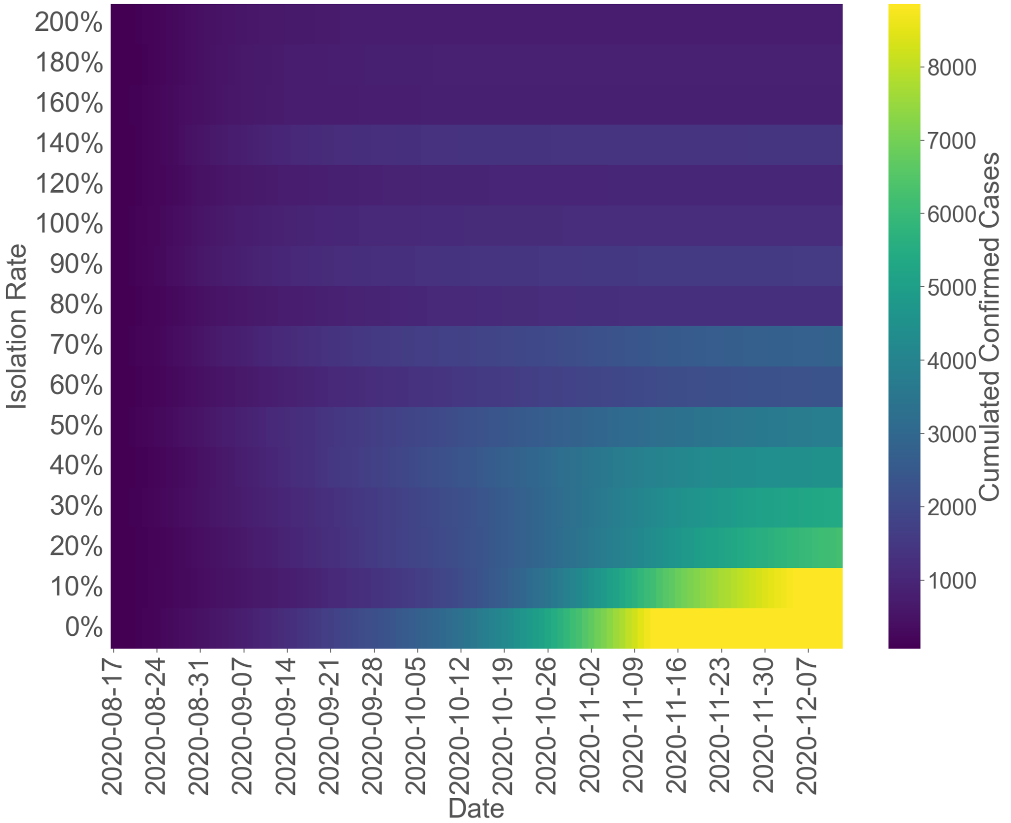

Fig S45. Cumulative confirmed cases of the hypothetical outbreaks over the Purdue campus with alternative weekly isolation rates under surveillance testing. A marked difference is observed between implementing isolation rates for asymptomatic cases below 50% per week and rates above 50% per week. A notable outbreak occurs with large spikes towards the end of the Fall 2020 semester in the hypothetical spreading scenarios, when the weekly isolation rate under surveillance testing for asymptomatic cases is set at 50% or lower per week.

environmental conditions and assumptions during the reconstruction of the testing setup. Nonetheless, with precise information about the spread, the proposed methods offer valuable insights into the isolation rates and their threshold conditions. The next section delves into the prospect of altering isolation rates during the semester based on the epidemic's severity, introducing a closed-loop feedback control algorithm.

SI-2-G. A Closed-Loop Feedback Control Algorithm

In Section SI-2-F, we study the impact of implementing different fixed isolation rates, i.e., open-loop control strategies, on the hypothetical outbreaks at the UIUC and Purdue campuses. When the isolation rate exceeds a certain threshold, significant reductions in peak infections and cumulative cases can be achieved. Therefore, it is reasonable to implement a relatively higher isolation rate on the campus to ensure safe operations. The success of implementing higher isolation rates to mitigate the spread of the virus has been demonstrated by both UIUC and Purdue University. While Purdue conducted 5000 tests for surveillance testing (S5 Fig in SI), they also performed a significant number of voluntary tests on symptomatic and suspected infection cases (S4 Fig in SI). However, for other settings like industrial parks, implementing high testing

and/or isolation rates poses challenges due to resource constraints. Maintaining a high testing and/or isolation rate over an extended period, such as several months, can be costly in terms of testing kits, human resources, testing sites, and other resources.

Therefore, it becomes crucial to design epidemic mitigation strategies that can potentially save resources while still keeping the infection level below a safe threshold. To achieve this goal, feedback control design methodologies can be leveraged. Feedback control systems are prevalent in both natural and engineered systems. In natural systems, such as the human body, complex feedback control mechanisms work to maintain various levels within specific ranges, such as body temperature, blood pressure, and blood sugar. Similarly, well-designed feedback control engineering systems (e.g., air conditioning systems) monitor systems and their states (room temperature) and adjust system inputs to maintain the states (temperature) at a desired level. Utilizing feedback control policies enhances robustness against disturbances in the spreading environment. Thus, even in the presence of uncertainties or inaccuracies in assessing the current situation [37], the feedback control system can dynamically adjust epidemic mitigation strategies to effectively respond to changing conditions and maintain the spread of the virus within manageable levels.

Effective mitigation strategies are crucial during epidemics like the recent COVID-19 pandemic. Researchers have applied systems and control theory to address various epidemic mitigation challenges [38–47, 47–50, 50–53], including modeling, estimating, and controlling the spread of epidemics, particularly in the context of the COVID-19 pandemic [41, 45, 51, 52, 54–57]. Some studies have investigated policies, such as the "on-off" policy [44], which assesses the social and economic costs of strict social distancing measures, and calibrated epidemic models to examine the impact of social distancing restrictions [58]. Other strategies have optimized epidemic mitigation by combining molecular and serology testing [51] and addressing the vulnerabilities of optimal approaches [41]. In addition to optimal control methods, control scientists and engineers have explored model predictive control frameworks [44, 45, 45–48, 48–50, 56, 57] and other strategies [59–61] to develop optimal or suboptimal epidemic mitigation policies. For instance, one study identified vaccination targets using transmission network structures [60]. Furthermore, there are other studies that consider epidemic mitigation and resource allocation [39, 53, 62–68]. Regarding epidemic state observation problems, researchers proposed a comprehensive closed-loop framework that includes a nonlinear observer for estimating system states, forecasting the spread, and controlling outbreaks [69]. Furthermore, recognizing uncertainties in implementing theoretical control designs in real-world scenarios is crucial [37, 52]. However, current control analyses and designs are largely reliant on epidemic compartmental models. Few control designs directly employ the effective reproduction number as the control goal and feedback information to adjust the intensity of the intervention strategy.

In order to introduce and validate our proposed closed-loop feedback control algorithm, we first present the optimal resource allocation problem for testing-for-isolation as the following control design challenge. This issue serves as inspiration for our closed-loop feedback control algorithm. Our goal is to mitigate the epidemic by minimizing the total number of tests (usually proportional to the isolation rate) during each period of the epidemic, as demonstrated in the following cost function

$$J(u(t)) = \int_{t_0}^{t_0+T} u(\tau) d\tau, \tag{22}$$

where $[t_0, t_0 + T]$ is the period of consideration and $u(t)$ denotes the isolation rate at time step t. Further, to simplify the formulation, we assume the testing rate is equal to the isolation rate. For instance, conducting random daily tests on 20% of the total population allows the identification and isolation of $20\% \times 7$ of the infectious cases each

week, evenly split across the week. Furthermore, $u(t) \in [\underline{u}, \overline{u}]$, where $\underline{u}$ and $\overline{u}$ represent the lower and upper bounds on isolation rates, respectively. To derive the testing-for-isolation strategy that minimizes the accumulated isolation rates during the period and simultaneously ensures that the number of infected individuals remains below a desired threshold $\bar{I}$, we formulate the following optimization problem,

$$\min_{u(t), t_0 \leq t \leq t_0+T} \; J(u(t)) \tag{23a}$$

$$\text{s.t. } \dot{\boldsymbol{x}}(t) = f(\boldsymbol{x}(t), u(t)), \tag{23b}$$

$$0 \leq I(t) \leq \bar{I}, \underline{u} \leq u(t) \leq \overline{u}, \tag{23c}$$

where $\dot{\boldsymbol{x}}(t) = f(\boldsymbol{x}(t), u(t))$ denotes the unknown spreading dynamics. We use $\boldsymbol{x}(t)$ to represent all possible spreading states and $I(t)$ to represent infected cases at any given time t. The upper bound $\bar{I}$ captures factors on the infection threshold like available public resources, such as hospital capacity. As we lack direct access to the model $\dot{\boldsymbol{x}}(t) = f(\boldsymbol{x}(t), u(t))$, we cannot solve the optimization problem directly. Nevertheless, we will establish a connection between the solution for the optimization problem and the effective reproduction number.

Based on previous analyses [51, 70], when T is sufficiently large to ensure the epidemic fades away by T in Eq (23), the optimal solution is to adjust the isolation rate to maintain the effective reproduction number at one when the infected population hits the infection threshold $\bar{I}$ (assuming we have the ability to adjust the isolation rate arbitrarily). Theoretically, when the effective reproduction number is sustained at one and the infected population equals the infection threshold $\bar{I}$, the number of new infections will stabilize at the infection threshold $\bar{I}$. Under these conditions, based on Eq (1), a higher isolation rate will consume additional testing resources but will result in a decrease in the number of daily new infections. However, since the primary objective is to prevent the number of new infections from surpassing the infection threshold $\bar{I}$, it becomes unnecessary to allocate additional resources if we can maintain the reproduction number at one through a lower isolation rate. In practice, due to uncertainties arising from modeling, estimation, and computation, maintaining the effective reproduction number at one is not robust against these disturbances, which may lead to the effective reproduction number easily exceeding one.

When the isolation rate maintains the effective reproduction number at one, any lower isolation rate will result in an increase in the infected population, surpassing the infection threshold, as the effective reproduction number will exceed one. Furthermore, observations from the successful implementation of testing-for-isolation strategies at both UIUC and Purdue revealed that the estimated effective reproduction number fluctuated around one, as shown in S16 and S17 Figs in SI. Therefore, drawing inspiration from both theoretical analyses and real-world testing-for-isolation results, we aim to simplify the control problem formulated in Eq (23) to the goal of maintaining the effective reproduction number at a certain value $\mathcal{R}_t^*$ ($\mathcal{R}_t^* \in (0, 1]$) during the outbreak. We validate this idea in the reconstructed hypothetical spreading environments for UIUC (S22 Fig in SI) and Purdue (S24 Fig in SI). Additionally, we aim to compare the effectiveness of our proposed closed-loop feedback control algorithm with the fixed isolation rates that were implemented at UIUC and Purdue.

In order to implement the concept of controlling the effective reproduction number at $\mathcal{R}_t^*$, we need to 1) propose the closed-loop feedback control algorithm, and 2) design the method to update the control input, i.e., the isolation rate, based on the feedback information (the estimated effective reproduction number). We will illustrate the control algorithm and how it is applied in practice with an example. Consider a scenario where the isolation rate can be adjusted bi-weekly. We implement a reasonable weekly isolation rate $u_1(t)$ for the first two weeks (the daily isolation rate is then given

by $u_1(t)/7$), as shown in the top plot of S46 Fig in SI. After the first two-week period, at the beginning of the third week, we leverage the confirmed cases from the first and second weeks to estimate the effective reproduction number $\mathcal{R}_t(u_1(t)/7)$ during weeks one and two. Using the default serial interval distribution w, the scaling factor computed through Eq (17), and the estimation method outlined in Section SI-2-D, we can obtain the average estimated effective reproduction number for weeks one and two. If the average estimated effective reproduction number matches our target value, $\mathcal{R}_t^*$, we maintain the same isolation rate, setting $u_2(t) = u_1(t)$ for week three and week four. Hence, the daily isolation rate for the next two weeks is $u_2(t)/7$. However, if the estimated effective reproduction number does not align with $\mathcal{R}_t^*$, we compute a new isolation rate $u_2(t)$ to regulate the effective reproduction number at $\mathcal{R}_t^*$ for the following two weeks. This process is repeated iteratively, adapting the isolation rate based on the estimated effective reproduction number, thereby establishing a closed-loop feedback control framework.

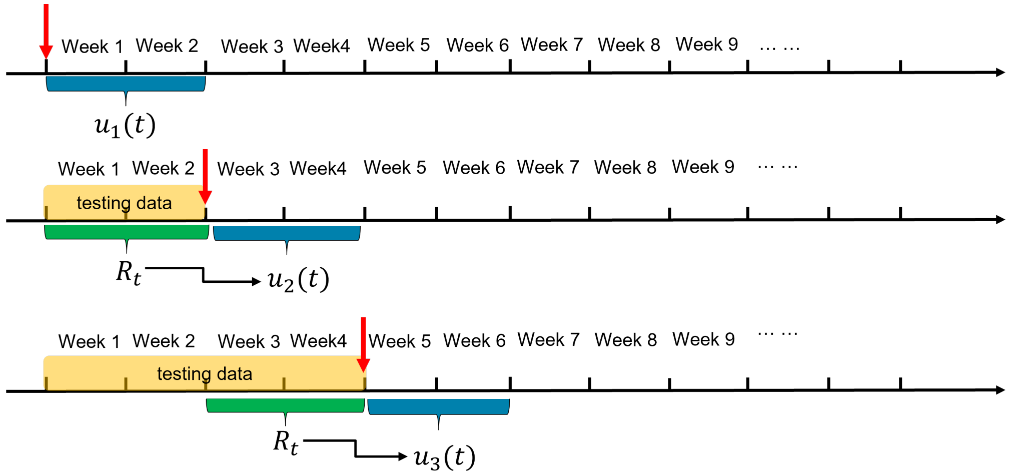

Fig S46. Illustration of the closed-loop feedback control framework. The framework updates the weekly isolation rate based on the effective reproduction number estimated from past data. By comparing the estimated effective reproduction number with the target effective reproduction number ($\mathcal{R}_t^*$), the framework adjusts the isolation rate for the next control period based on the severity of the pandemic.

After introducing the general feedback control framework, we now explain how to update the isolation rate at each iteration. For simplicity, we do not differentiate between symptomatic and asymptomatic cases. Therefore, we use $v \in \mathbb{R}^n_{\geq 0}$ to represent the initial infection profile and $w \in \mathbb{R}^n_{\geq 0}$ to represent the corresponding serial interval distribution of the spreading process. However, the developed techniques can be applied to compute isolation rates for infections that involve a mix of symptomatic and asymptomatic cases using the mechanisms proposed in Eqs (2) and (12).

Consider an epidemic spreading process with a known time-invariant serial interval distribution (e.g., Eq (6)). At a specific step $k > 1$, that captures the first two weeks in the previous example, we can estimate the effective reproduction number $\mathcal{R}_k(\alpha_k)$ under the implemented daily isolation rate α_k during the first two weeks, using the method proposed in Section SI-2-D. Based on this estimated effective reproduction number $\mathcal{R}_k(\alpha_k)$, we define the following algorithm to update the daily isolation rate α_{k+1} for the next time step $k+1$. From the effective reproduction number $\mathcal{R}_k(\alpha_k)$, we first

compute the infection profile that captures the spread at step k, given by

$$v'(\alpha_k) = \sum_{i=1}^{n} w(\alpha_k)\mathcal{R}_k(\alpha_k),$$

where α_k is the fixed daily isolation rate at step k. The serial interval distribution w under the daily isolation rate α_k is denoted as $w(\alpha_k)$ in Eq (16). If the estimated effective reproduction number under the daily isolation rate α_k at step k is not equal to the target effective reproduction number $\mathcal{R}_t^*$, i.e., $\mathcal{R}_k(\alpha_k) \neq \mathcal{R}_t^*$, we compute a new daily isolation rate α_{k+1} for the next step $k+1$ (e.g., the following two weeks in the previous example).

Therefore, we propose the following algorithm to update the daily isolation rate α_{k+1} for the next time step $k+1$:

$$\frac{1}{\mathcal{F}(\alpha_k)} \sum_{i=1}^{n} v_i'(\alpha_k)(1-\alpha_{k+1})^i = \frac{\mathcal{R}_k(\alpha_k)}{\mathcal{F}(\alpha_k)} \sum_{i=1}^{n} w_i(\alpha_k)(1-\alpha_{k+1})^i = \mathcal{R}_t^*. \quad (24)$$

The only unknown variable in Eq (24) is the daily isolation rate for the subsequent step, α_{k+1}. Additionally, Eq (24) is a polynomial equation for the isolation rate to be updated, which is not difficult to solve. The term $(1-\alpha_{k+1})^i$ is a scaling factor for $v_i'(\alpha_k)$, $i \in \{1, 2, \ldots, n\}$. Hence, Eq (24) is a monotonic decreasing function of α_{k+1}, when $\alpha_{k+1} \in [0, 1]$. Therefore, there must be only one solution to the equation when $\alpha_{k+1} \in [0, 1]$. By solving Eq (24), we can directly compute the daily isolation rate for the next step using the feedback information derived from the estimated effective reproduction number $\mathcal{R}_k(\alpha_k)$ and the pre-defined serial interval distribution w, under the implemented daily isolation rate α_k at step k. The algorithm proposed in Eq (24) relies on the estimated effective reproduction number to generate the new daily isolation rate, making it a closed-loop feedback control approach for epidemic control problems. As a feedback control mechanism, Eq (24) is more robust to uncertainties compared to generating the isolation rate through an open-loop mechanism.

We use the reconstructed hypothetical spreading environment at UIUC and Purdue to validate the closed-loop feedback control algorithm in Eq (24). Although the algorithm is based on the daily isolation rate, we discuss the weekly isolation rate by multiplying the daily isolation rate by seven in the simulations, as UIUC and Purdue implemented the testing-for-isolation strategy on a weekly basis. As a result, our control design goal is to maintain the effective reproduction number at $\mathcal{R}_t^*$, which is slightly less than one [37]. We initially implement the feedback control framework in the reconstructed hypothetical spreading environment at the UIUC campus during Fall 2020. This setting allows us to compare the feedback control algorithm with the fixed weekly isolation rate implemented at UIUC, involving testing the entire campus twice a week.

Consider the same worst-case hypothetical spreading scenario and assumptions for the UIUC campus as discussed in Sections SI-2-C and SI-2-F, we implement the feedback control framework proposed in this section. We then compare it to UIUC's testing-for-isolation strategy implemented in the same environment, which assumes a weekly isolation rate of 200%, i.e., 2/7 daily isolation rate. Our objective is to control the effective reproduction number at $\mathcal{R}_t^* = 0.95$. The simulation results are shown in S47 Fig in SI. Using the hypothetical worst-case spreading scenario at UIUC, as captured by S22 Fig in SI, UIUC's strategy is to test everyone the isolate all positive cases twice a week, and our strategy is generated by the proposed feedback control algorithm from Eq (24).

S47 Fig in SI demonstrates that the feedback control framework we proposed performs similarly to the testing-for-isolation policy implemented by UIUC in terms of total confirmed cases, for a total of around 5000. Both approaches maintain the

infection level below 150 daily confirmed cases. Furthermore, through simulation, under the condition that the weekly isolation rate is proportional to the number of tests, we find that the implemented testing-for-isolation strategy by UIUC will result in a total of 32 tests per individual, whereas our proposed feedback control strategy will only require 28 tests per individual. Throughout most of the Fall 2020 semester, the feedback control algorithm utilizes a lower daily thus weekly isolation rate compared to UIUC's 200% weekly isolation rate. However, in October, the feedback control framework applies higher weekly isolation rates than the weekly rates implemented by UIUC. Recall that real-world confirmed data (S3 Fig in SI) and the estimated effective reproduction number (S16 Fig in SI) at UIUC during Fall 2020 reveal a significant spike in confirmed cases due to the return of the football season. Consequently, the feedback control framework raises the daily (weekly) isolation rate to mitigate the potential outbreak. In summary, the feedback control framework implements fewer isolation thus tests when there is a lower risk of outbreaks and increases the isolation rate when there is a potential spike. This example effectively illustrates the core concept of the closed-loop feedback control algorithm proposed for the pandemic mitigation framework, where we can leverage the connection between the strength of the intervention strategy and the effective reproduction number to design a feedback control mechanism.

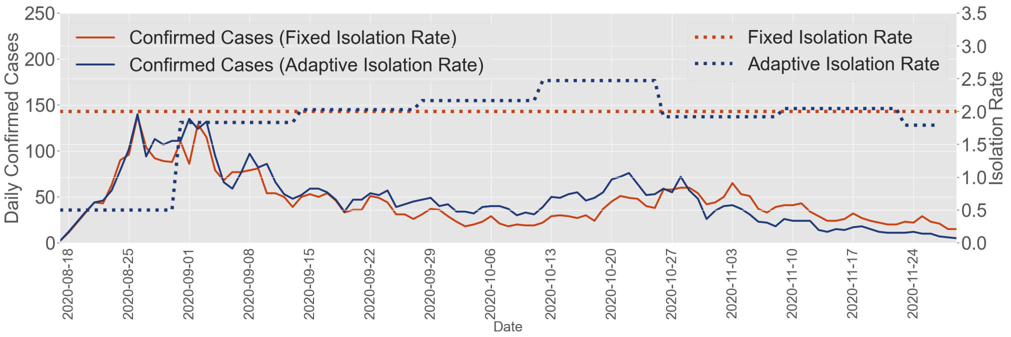

Fig S47. Comparison between the fixed and feedback testing-for-isolation strategies at UIUC. When controlling the effective reproduction number at 0.95, the feedback control algorithm we proposed performs similarly to the testing-for-isolation policy implemented by UIUC in terms of total confirmed cases. Under the condition that the isolation rate is proportional to the number of tests, the implemented testing-for-isolation strategy by UIUC will result in a total of 32 tests per individual, whereas our proposed feedback control strategy will only require 28 tests per individual.

In addition to aiming to match the confirmed cases under the testing-for-isolation strategy implemented by UIUC (200% weekly testing and isolation rate), it is also interesting to investigate if the feedback control framework can generate more conservative testing-for-isolation strategies with higher weekly isolation rate. In this case, we adjust the goal of controlling the target effective reproduction number at $\mathcal{R}_t^* = 0.90$. The resulting confirmed cases and corresponding weekly isolation rates of this hypothetical outbreak are presented in S48 Fig in SI. When aiming to control the effective reproduction number at $\mathcal{R}_t^* = 0.90$, we observe fewer daily and total confirmed cases during Fall 2020 compared to the 200% weekly isolation rate. While the 200% isolation rate results in around 5000 total confirmed cases, the feedback control framework generates approximately 3500 confirmed cases during the same period.

Although the feedback control framework requires around the same number of tests, specifically 31 tests per person during Fall 2020 compared to the 32 tests under the 200% isolation rate, the total number of confirmed cases is reduced by approximately 45%. The significant reduction in total confirmed cases, with a similar amount of

testing resources, is due to the feedback control framework saving resources when there is no immediate risk of potential outbreaks, allowing for increased isolation rate during potential spikes, as shown in S48 Fig in SI. Specifically, the feedback control algorithm increases the daily and thus weekly isolation rate during a potential outbreak in October. By utilizing the estimated effective reproduction number to identify potential outbreaks, the feedback control framework adjusts the isolation rate accordingly. If we distribute the tests uniformly instead of implementing the feedback control framework to save tests for targeting potential outbreaks, simply adding one test per person to the implemented testing-for-isolation strategy during Fall 2020 might not have mitigated the spread to the same extent achieved by the feedback control framework.

Both S47 and S48 Figs in SI demonstrate the potential value of the proposed closed-loop feedback control algorithm in the context of the testing-for-isolation strategy. Leveraging the estimated effective reproduction number at each iteration to update the isolation rate helps correct uncertainties associated with the method in quantifying the impact of the isolation rate on the serial interval distribution. Moreover, the core idea behind this control algorithm shares similarities with model predictive control techniques, which employ feedback design to learn the model while designing control strategies [42].

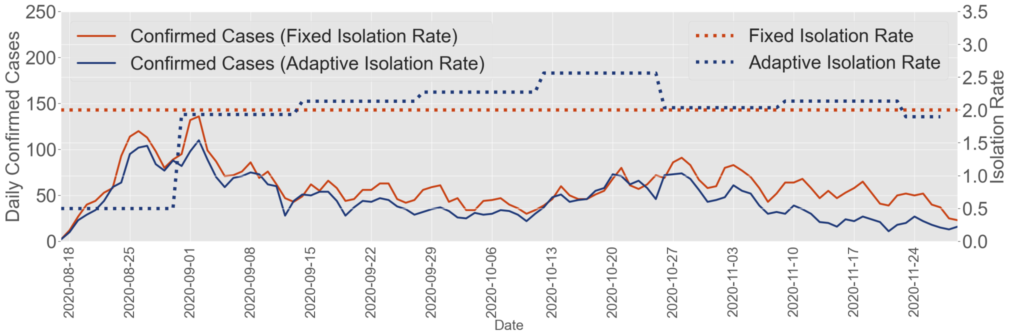

Fig S48. Comparison between the fixed and feedback testing-for-isolation strategies at UIUC. The goal is to control the effective reproduction number at 0.90. Under these circumstances, while the 200% weekly isolation rate results in around 5000 total confirmed cases, the feedback control framework generates approximately 3600 confirmed cases during the same period, using the same amount of testing resources. Specifically, the feedback control framework increases the weekly isolation rate during a potential outbreak in October.

After implementing the control algorithm in the hypothetical spreading scenario at the UIUC campus, we proceed to study the framework on the Purdue campus. As discussed earlier, we can generalize the closed-loop feedback control algorithm in Eq (24) to encompass spreading with both symptomatic and asymptomatic infections. Therefore, we investigate the closed-loop feedback control framework over the Purdue campus, where the hypothetical spreading scenario is based on the specific reconstruction in S24 Fig in SI. The hypothetical spreading scenario at Purdue in S24 Fig in SI allows for a 30% weekly isolation rate for asymptomatic infections and involves a symptomatic infection ratio of $\theta = 55\%$.

Unlike UIUC, where we do not distinguish between symptomatic and asymptomatic infections while implementing the testing-for-isolation strategy, at Purdue, we adjust the daily thus weekly surveillance isolation rate for asymptomatic infections. Similar to the implementation of different fixed isolation rates for the Purdue campus in Section SI-2-F, we assume all symptomatic cases will test and isolate themselves within a week once they become infectious and symptomatic. Thus, we consider that all

symptomatic infections will be detected through voluntary testing-for-isolation strategy, resulting in a 100% weekly isolation rate (1/7 daily isolation rate) for symptomatic infections. Based on the effective reproduction number $\mathcal{R}_k$ under the daily isolation rate for asymptomatic cases $\overline{\alpha}_k$ and the daily isolation rate for symptomatic cases $\underline{\alpha}_k$ at step k, along with the defined initial serial interval distributions $\overline{w}$ and $\underline{w}$ for asymptomatic and symptomatic infections, respectively, the modified infection profile is given by

$$\sum_{i=1}^{n} v_i'(\overline{\alpha}_k) = \theta \sum_{i=1}^{n} \underline{w}_i(\underline{\alpha}_k)\mathcal{R}_k(\alpha_k) + (1-\theta)\sum_{i=1}^{n} \overline{w}_i(\overline{\alpha}_k)\mathcal{R}_k(\alpha_k).$$

We define $\mathcal{R}_k(\alpha_k)$ as the (estimated) effective reproduction number under the overall daily isolation rate ($\alpha_k = \theta\underline{\alpha}_k + (1-\theta)\overline{\alpha}_k$) for symptomatic ($\underline{\alpha}_k$) and asymptomatic ($\overline{\alpha}_k$) infections at step k. The daily isolation rate for symptomatic cases will remain unchanged, i.e., $\underline{\alpha}_k = \underline{\alpha}_{k+1} = 1/7$ in this example. If $\mathcal{R}_k(\alpha_k) \neq \mathcal{R}_t^*$, we compute a new daily isolation rate for asymptomatic cases $\overline{\alpha}_{k+1}$ during the next step $k+1$. Based on the proposed feedback control algorithm in Eq (24), we can further compute the updated daily isolation rate for asymptomatic cases $\overline{\alpha}_{k+1}$ by solving the equation:

$$\frac{\mathcal{R}_k(\alpha_k)}{\mathcal{F}(\alpha_k)} \sum_{i=1}^{n} \theta \underline{w}_i(\underline{\alpha}_k)(1-\underline{\alpha}_k)^i + \frac{\mathcal{R}_k(\alpha_k)}{\mathcal{F}(\alpha_k)} \sum_{i=1}^{n} (1-\theta)\overline{w}_i(\overline{\alpha}_k)(1-\overline{\alpha}_{k+1})^i = \mathcal{R}_t^*. \quad (25)$$

We use the same mechanism in Eq (13) to define $\overline{w}(\overline{\alpha}_k)$ ($\underline{w}(\underline{\alpha}_k)$) under the daily isolation rate $\overline{\alpha}_k$ ($\underline{\alpha}_k$). Thus, the only unknown daily isolation rate we will update in Eq (25) is for the asymptomatic infection, i.e., $\overline{\alpha}_{k+1}$.

Recall that we use the same serial interval distribution for symptomatic and asymptomatic infections, i.e., $\underline{w} = \overline{w} = w$. By solving Eq (25), we can compute the updated daily isolation rate for asymptomatic infections. Furthermore, Eq (25) can be considered as a generalized formulation of Eq (24), where we can substitute different serial interval distributions for symptomatic and asymptomatic infections ($\underline{w}$ and $\overline{w}$), the ratio of the symptomatic infection (θ), and the target effective reproduction number $\mathcal{R}_t^*$. Moreover, if we want to update both symptomatic ($\underline{\alpha}$) and asymptomatic daily isolation rates ($\overline{\alpha}$), we can substitute $\underline{w}_i(\underline{\alpha})$ and $(1-\underline{\alpha})^i$ with $\underline{w}_i(\underline{\alpha}_k)$ and $(1-\underline{\alpha}_{k+1})^i$ in Eq (25), respectively. Then, we can utilize the updated equation to establish a relationship between the isolation rates $\underline{\alpha}_{k+1}$ and $\overline{\alpha}_{k+1}$ to be solved. Based on this relationship and the available testing-for-isolation resources, we can allocate resources to the symptomatic (voluntary) and asymptomatic (surveillance) isolation rates accordingly.

By implementing the proposed closed-loop feedback control algorithm in Eq (25) into the hypothetical spreading scenario at Purdue, as shown in S24 Fig in SI, our goal is to ensure that the feedback control strategy performs similarly to the testing-for-isolation strategy at Purdue in terms of confirmed cases during Fall 2020. To achieve this goal, we set the target effective reproduction number slightly higher than one, specifically at $\mathcal{R}_t^* = 1.05$. Ideally, $\mathcal{R}_t^*$ needs to be smaller than one. We conduct simulations in the reconstructed environment at Purdue during Fall 2020 (S24 Fig in SI), considering the strategies implemented by Purdue and our proposed feedback control algorithm based on Eq (25). Illustrated by the solid lines in S49 Fig in SI, we observe around 5200 total confirmed cases under the testing-for-isolation strategy implemented by Purdue and our closed-loop feedback control algorithm. The feedback control strategy can adjust the isolation rates for asymptomatic cases. In addition, the feedback control strategy generates a similar peak number of confirmed cases compared to the spreading process generated by Purdue's fixed isolation rates.

We plot the weekly isolation rates for both strategies with dotted lines. Comparing the fixed surveillance isolation rate implemented by Purdue (30% per week, represented by the dark grey dotted line) and the isolation rate generated by our feedback control

framework (average 28% per week, represented by the yellow, dotted line) in S49 Fig in SI, we notice that the feedback control framework significantly increases the isolation rate for asymptomatic cases, particularly during October 2020. This outcome is in line with the observation made in Section SI-1, where both UIUC and Purdue experienced substantial spikes in the infected population on campus during that month, primarily due to various gathering events associated with the college football season. Thus, similar to the simulation for UIUC, the feedback control framework adjusts the isolation rates for asymptomatic cases, as indicated by the dark grey, dotted line in S49 Fig in SI, surpassing the fixed isolation rates implemented by Purdue from mid-October 2020 to mid-November. The simulation result suggests that we can allocate testing-for-isolation resources more efficiently to mitigate potential outbreaks.

In addition to evaluating the isolation needs for asymptomatic cases to achieve the same number of confirmed cases as at Purdue, we establish the mitigation problem's objective to control the target effective reproduction number at $\mathcal{R}_t^* = 0.95$. Using the same reconstructed hypothetical spreading scenario as in the prior example to capture the spread across the Purdue campus, we implement both the testing-for-isolation strategies from Purdue and our proposed feedback control algorithm based on Eq (25). We illustrate the confirmed cases with solid lines and the weekly isolation rates with dotted lines in S50 Fig in SI.

Comparing the confirmed cases resulting from Purdue's fixed isolation rate (yellow, solid lines) and our control framework (dark, solid lines), we observe that our control framework effectively prevents potential outbreaks, especially from mid-October 2020 to mid-November, by implementing a much higher isolation rate for surveillance testing. Additionally, while the fixed isolation rate would result in around 5200 confirmed cases during Fall 2020, our isolation rates based on the feedback control algorithm would yield approximately 2800 confirmed cases. To achieve a decrease of about 30% in the total number of confirmed cases, the feedback control strategy would implement an average 59% isolation rate for asymptomatic infections. These resources are predominantly utilized from mid-October 2020 to mid-November 2020. Despite the feedback control strategy requiring a higher isolation rate for asymptomatic cases, it effectively helps prevent potential outbreaks during the middle of the Fall 2020 semester on the Purdue campus.

In this section, we design a closed-loop feedback control algorithm that utilizes the (estimated) effective reproduction number as feedback information to automatically update the isolation rate. This algorithm is based on the proposed feedback control method in Eqs (24) and (25). We validate the application of the method by comparing the implemented testing-for-isolation strategies with the strategies generated by the closed-loop feedback control framework in the hypothetical spreading scenarios at both the UIUC and Purdue campuses. Hence, in this work, we utilize the reconstructed hypothetical spreading environment to execute the feedback control framework. This choice is due to our access solely to historical data, allowing us to illustrate and validate our framework. In real-time scenarios where we have access to current spreading data, we can obtain all the necessary information to directly update the isolation rate in the near future based on the proposed feedback control framework. However, we still recommend using a reconstructed hypothetical spreading environment to evaluate the potential impact of different isolation rates first. As demonstrated in Section SI-2-E, the results generated through the framework depend on conditions related to population behavior and the characteristics of the spreading process. In addition, adjusting pandemic mitigation policies inappropriately for real-world spread may result in large outbreaks.

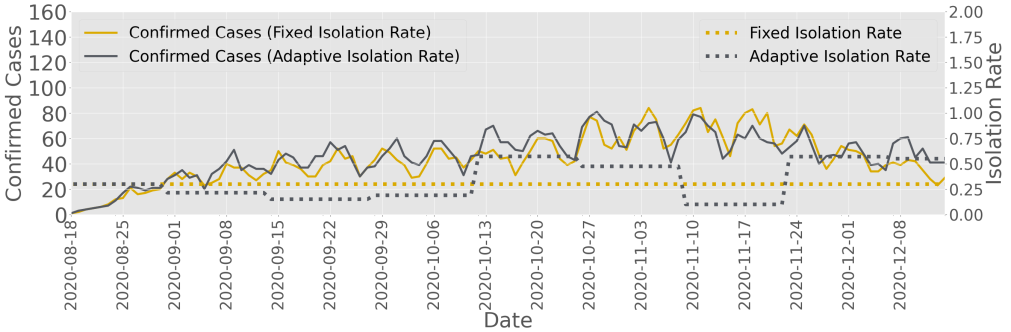

Fig S49. Comparison between the fixed and feedback testing-for-isolation strategies in the hypothetical spreading environment at Purdue. The target effective reproduction number is set at 1.05, slightly higher than one, in order to generate a total number of confirmed cases similar to those observed on the Purdue campus during Fall 2020. Comparing the fixed surveillance isolation rate implemented by Purdue (30% per week, represented by the dark, grey dotted line) with the isolation rate generated by our feedback control framework (average 28% per week, represented by the yellow, dotted line), it is evident that the feedback control framework significantly increases the isolation rate for asymptomatic cases, particularly from mid-October to mid-November 2020.

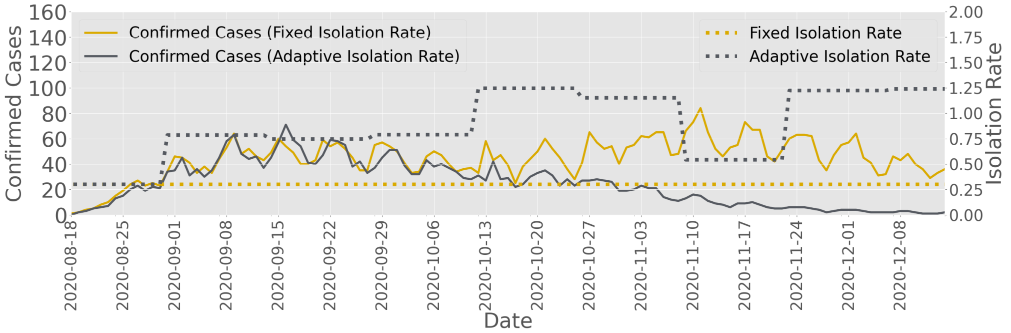

Fig S50. Comparison between the fixed and feedback testing-for-isolation strategies in the hypothetical spreading environment at Purdue. The target effective reproduction number is set at 0.95. While the fixed isolation rate would result in around 5200 confirmed cases during Fall 2020, our feedback isolation rates would yield approximately 2800 confirmed cases. To achieve a decrease of about 30% in the total number of confirmed cases, the feedback control framework would implement an average 59% weekly isolation rate for asymptomatic infections.

SI-3. Discussion

As indicated in S1 Fig in SI and the previous sections of this document, the quantification of the isolation rate's impact on the infection profile, serial interval distribution, and reproduction numbers is key to our framework for counterfactual analysis, strategy evaluation, and feedback control of epidemics. We develop two methods based on this quantification: 1) a method to reverse engineer the effective reproduction number, and 2) the other leverages the effective reproduction number to design a closed-loop feedback control algorithm. In real-world applications, both approaches rely on effective reproduction number estimates from infection data. However, solely relying on the reproduction number estimates for a comprehensive evaluation of a pandemic may pose several limitations and challenges in real-world

application. As indicated by the SHIELD team at UIUC, even with the estimated effective reproduction number slightly less than one, a large number of initial infected cases will still cause an outbreak on campus [4]. Hence, in this section, we delve into potential limitations and challenges of our proposed framework and methods by examining the restrictions associated with relying solely on the reproduction number estimates.

The reproduction number estimates can indicate the trend in the newly infected population. However, relying solely on the reproduction number estimates may result in incorrect assessments of the spread if the precise number of infected or confirmed cases is not considered. For example, consider the estimated effective reproduction numbers for UIUC in S16 Fig in SI and Purdue in S17 Fig in SI during Fall 2020. The highest estimated effective reproduction number (mean) at UIUC is approximately 1.3, while at Purdue, it is around 1.1. Furthermore, the estimated effective reproduction number (along with the 95% confidence interval) for UIUC shows higher variance compared to Purdue. If we are to base our judgment solely on the estimated effective reproduction number, one may infer that Purdue performed better in mitigating the outbreak on campus. However, when comparing the confirmed cases at UIUC in S3 Fig in SI and Purdue in S8 Fig in SI during Fall 2020, it is apparent that UIUC reported significantly fewer daily confirmed cases and lower spikes in terms of peak infections. Additionally, UIUC conducted more tests, potentially leading to more accurate detection of infection numbers compared to Purdue. Therefore, what could be amiss with our analysis? This disparity occurs because the effective reproduction number captures relative changes in infected cases but disregards absolute changes. Since UIUC had fewer confirmed cases than Purdue on most days, even a small increase in confirmed cases at UIUC would result in a larger rise in the effective reproduction number compared to Purdue.

To address this issue, it is critical to integrate the analysis of the effective reproduction number with the assessment of infected or confirmed cases. In our proposed framework, our objective is to regulate the effective reproduction number to the target value $\mathcal{R}_t^*$, as shown in Eqs (24) and (25). Nonetheless, as discussed, if the daily infected cases are notably high, managing the effective reproduction number slightly below one might still yield a high daily infected population, potentially resulting in an outbreak or leading to a slow reduction in the daily infected cases. To prevent such circumstances, we can enhance our framework, particularly the closed-loop feedback control algorithm, by considering both infected or confirmed cases and the effective reproduction number as feedback information. If the daily confirmed cases substantially exceed our target threshold, upon updating the isolation rate, we can select a target reproduction number $\mathcal{R}_t^*$ considerably lower than one. Conversely, if the daily confirmed cases fall far below our target threshold, as per the analyses in Section SI-2-G, the framework may allocate fewer testing-for-isolation resources than necessary. In summary, when deploying the closed-loop feedback control framework for epidemic control, it is critical to consider both the effective reproduction number and the daily infected cases (or related factors) to overcome the limitations of relying solely on the reproduction number estimates. By adapting the target effective reproduction number based on different disease-spreading indicators, we can enhance the precision and effectiveness of the framework.

SI-4. Future Works

We acknowledge several limitations of the proposed framework and methods and provide potential avenues for improvement through future work. First, when estimating the effective reproduction number, we rely on existing infection profiles (serial interval distributions) from the literature. However, different communities may experience

different spreading behavior. Therefore, it is critical to incorporate contact tracing data from the testing-for-isolation strategies to update the infection profile and the serial interval distribution during the pandemic.

Furthermore, in our control strategy design, we assume that the effective reproduction number estimated in the past will remain unchanged in the near future within a short time window, provided the testing-for-isolation strategy is not altered. In reality, the effective reproduction number can vary due to factors such as seasonal changes, breaks or events on campus, changes in student behavior, viral loads, and virus mutations. Thus, incorporating a predictive control mechanism with machine learning techniques to account for these fluctuations is a challenging yet important extension [71].

Additionally, the goal of maintaining the effective reproduction number at $\mathcal{R}_t^*$, slightly smaller than one, aligns with the objective of keeping the infected population at an acceptable level for both universities. However, if the initial infected population is substantial, maintaining the effective reproduction number at a fixed value slightly below one can still lead to a large number of infections. Therefore, adjusting the goal of the control framework becomes highly significant in real-world implementations [72, 73].

While the framework has the potential to save mitigation resources, implementing frequent changes in the testing-for-isolation policy as designed in the control framework (e.g., weekly or bi-weekly adjustments) may be unrealistic in practice. Even in more flexible environments such as universities, UIUC and Purdue maintained their policies constant for an entire semester. In addition, the isolation rate generated by the feedback control strategy may exceed the resource capacity of an authority. Hence, exploring an optimization problem on the developed framework, with constraints on the control input, the isolation rate, is also necessary in the future.

We leverage aggregated data to validate the framework and perform all analyses. We can enhance the framework by incorporating spatial and heterogeneous spreading data. For instance, both UIUC and Purdue have data that can facilitate the construction of contact-tracing networks. We can estimate the effective reproduction number to characterize the spread in sub-regions and adjust intervention strategies for each region separately, based on the severity of their outbreak and their interactions with other sub-regions. By leveraging high-resolution data, future work can focus on studying a distributed strategy evaluation and feedback control framework that allocates varying degrees of intervention strategies based on the severity of the spread in different connected regions. In addition, the connections between different regions can be inferred through machine learning techniques such as graph learning and causal inference.

Another potential limitation in the feedback control framework is the absence of predictions for the effective reproduction number in the near future. We have discussed the drawback of lacking predictions when updating the testing/isolation rates in Section SI-2-G. While the feedback control mechanism helps mitigate the uncertainties introduced by the current framework, it is still important to have predictions of the effective reproduction number to generate and implement more robust testing and isolation rates and to anticipate potential changes in the epidemic spreading processes. Researchers have demonstrated the effectiveness of implementing reproduction number prediction method based on historical estimated effective reproduction number, as showcased in the *Epinow*2 package [3, 74, 75]. In our future work, we will explore the relationship between data sizes and prediction accuracy to further enhance the feedback control framework, by accounting for the uncertainty from the prediction. By incorporating predictive capabilities, we aim to improve the framework's ability to adapt to evolving epidemic dynamics and optimize epidemic mitigation strategies accordingly. Nevertheless, we believe that this work sparks discussions on the role and limitations of counterfactual analysis, strategy evaluation, and feedback control of epidemics using reproduction number estimates, from both analytical and computational perspectives.

Nevertheless, we believe that this work sparks discussions on the role and limitations of counterfactual analysis, strategy evaluation, and feedback control of epidemics using reproduction number estimates, from both analytical and computational perspectives. We believe that leveraging key model information, such as the reproduction number, from epidemic spreading processes can inspire and benefit the design of other modeling and control strategies in various research fields.

References

1. Cori A, Ferguson NM, Fraser C, Cauchemez S. A new framework and software to estimate time-varying reproduction numbers during epidemics. American Journal of Epidemiology. 2013;178(9):1505–1512.

2. Huisman JS, Scire J, Angst DC, Li J, Neher RA, Maathuis MH, et al. Estimation and worldwide monitoring of the effective reproductive number of SARS-CoV-2. Elife. 2022;11:e71345.

3. Gostic KM, McGough L, Baskerville EB, Abbott S, Joshi K, Tedijanto C, et al. Practical considerations for measuring the effective reproductive number, R_t. PLoS Computational Biology. 2020;16(12):e1008409.

4. Ranoa DRE, Holland RL, Alnaji FG, Green KJ, Wang L, Fredrickson RL, et al. Mitigation of SARS-CoV-2 transmission at a large public university. Nature Communications. 2022;13(1):1–16.

5. Goyal R, Hotchkiss J, Schooley RT, De Gruttola V, Martin NK. Evaluation of SARS-CoV-2 transmission mitigation strategies on a university campus using an agent-based network model. Clinical Infectious Diseases: An Official Publication of the Infectious Diseases Society of America. 2021;.

6. Lopman B, Liu CY, Le Guillou A, Handel A, Lash TL, Isakov AP, et al. A model of COVID-19 transmission and control on university campuses. MedRxiv. 2020;.

7. Ghaffarzadegan N, Childs LM, TÄuber UC. Diverse computer simulation models provide unified lessons on university operation during a pandemic. BioScience. 2021;71(2):113–114.

8. Gressman PT, Peck JR. Simulating COVID-19 in a university environment. Mathematical Biosciences. 2020;328:108436.

9. Brown RA. A simple model for control of COVID-19 infections on an urban campus. Proceedings of the National Academy of Sciences. 2021;118(36):e2105292118.

10. Bahl R, Eikmeier N, Fraser A, Junge M, Keesing F, Nakahata K, et al. Modeling COVID-19 spread in small colleges. Plos One. 2021;16(8):e0255654.

11. Borowiak M, Ning F, Pei J, Zhao S, Tung HR, Durrett R. Controlling the spread of COVID-19 on college campuses. arXiv preprint arXiv:200807293. 2020;.

12. Kronbichler A, Kresse D, Yoon S, Lee KH, Effenberger M, Shin JI. Asymptomatic patients as a source of COVID-19 infections: A systematic review and meta-analysis. International Journal of Infectious Diseases. 2020;98:180–186.

13. Keeling MJ, Rohani P. Modeling Infectious Diseases in Humans and Animals. Princeton University Press; 2011.

14. Diekmann O, Heesterbeek JAP. Mathematical Epidemiology of Infectious Diseases: Model building, Analysis and Interpretation. vol. 5. John Wiley & Sons; 2000.

15. Fraser C. Estimating individual and household reproduction numbers in an emerging epidemic. PloS One. 2007;2(8):e758.

16. Delamater PL, Street EJ, Leslie TF, Yang YT, Jacobsen KH. Complexity of the basic reproduction number (R0). Emerging Infectious Diseases. 2019;25(1):1.

17. Goyal A, Reeves DB, Cardozo-Ojeda EF, Schiffer JT, Mayer BT. Viral load and contact heterogeneity predict SARS-CoV-2 transmission and super-spreading events. Elife. 2021;10:e63537.

18. He X, Lau EH, Wu P, Deng X, Wang J, Hao X, et al. Temporal dynamics in viral shedding and transmissibility of COVID-19. Nature Medicine. 2020;26(5):672–675.

19. Hilfiker L, Josi J. Epyestim: Application to COVID-19 data; 2022. `https://github.com/lo-hfk/epyestim`.

20. Lauer SA, Grantz KH, Bi Q, Jones FK, Zheng Q, Meredith HR, et al. The incubation period of coronavirus disease 2019 (COVID-19) from publicly reported confirmed cases: estimation and application. Annals of Internal Medicine. 2020;172(9):577–582.

21. Brauner JM, Mindermann S, Sharma M, Johnston D, Salvatier J, Gavenčiak T, et al. Inferring the effectiveness of government interventions against COVID-19. Science. 2021;371(6531):eabd9338.

22. Brauner JM, Mindermann S, Sharma M, Stephenson AB, Gavenčiak T, Johnston D, et al. The effectiveness and perceived burden of nonpharmaceutical interventions against COVID-19 transmission: A modelling study with 41 countries. medRxiv. 2020; p. 2020–05.

23. Tariq A, Lee Y, Roosa K, Blumberg S, Yan P, Ma S, et al. Real-time monitoring the transmission potential of COVID-19 in Singapore, March 2020. BMC Medicine. 2020;18(1):1–14.

24. Ali ST, Wang L, Lau EH, Xu XK, Du Z, Wu Y, et al. Serial interval of SARS-CoV-2 was shortened over time by nonpharmaceutical interventions. Science. 2020;369(6507):1106–1109.

25. Cori A, Kamvar Z, Stockwin J, Jombart T, Dahlqwist E, FitzJohn R, et al.. EpiEstim v2.2-4: A tool to estimate time varying instantaneous reproduction number during epidemics; 2022. `https://github.com/mrc-ide/EpiEstim`.

26. Thompson RN, Stockwin JE, van Gaalen RD, Polonsky JA, Kamvar ZN, Demarsh PA, et al. Improved inference of time-varying reproduction numbers during infectious disease outbreaks. Epidemics. 2019;29:100356.

27. Nash RK, Nouvellet P, Cori A. Real-time estimation of the epidemic reproduction number: Scoping review of the applications and challenges. PLOS Digital Health. 2022;1(6):e0000052.

28. Bhatia S, Wardle J, Nash RK, Nouvellet P, Cori A. A generic method and software to estimate the transmission advantage of pathogen variants in real-time: SARS-CoV-2 as a case-study. medRxiv. 2021; p. 2021–11.

29. Nash RK, Bhatt S, Cori A, Nouvellet P. Estimating the epidemic reproduction number from temporally aggregated incidence data: A statistical modelling approach and software tool. PLOS Computational Biology. 2023;19(8):e1011439.

30. Cauchemez S, Boëlle PY, Thomas G, Valleron AJ. Estimating in real time the efficacy of measures to control emerging communicable diseases. American Journal of Epidemiology. 2006;164(6):591–597.

31. Richardson WH. Bayesian-based iterative method of image restoration. JoSA. 1972;62(1):55–59.

32. Lucy LB. An iterative technique for the rectification of observed distributions. The Astronomical Journal. 1974;79:745.

33. Parag KV. Improved estimation of time-varying reproduction numbers at low case incidence and between epidemic waves. PLoS Computational Biology. 2021;17(9):e1009347.

34. Linka K, Peirlinck M, Kuhl E. The reproduction number of COVID-19 and its correlation with public health interventions. Computational Mechanics. 2020;66:1035–1050.

35. Sam Abbott, Joel Hellewell, Katharine Sherratt, Katelyn Gostic, Joe Hickson, Hamada S Badr, et al.. EpiNow2: Estimate Real-Time Case Counts and Time-Varying Epidemiological Parameters; 2020.

36. Centers for Disease Control and Prevention. Estimated COVID-19 Infections, Symptomatic Illnesses, Hospitalizations, and Deaths in the United States; 2021.

37. van Heusden K, Stewart GE, Otto SP, Dumont GA. Effective pandemic policy design through feedback does not need accurate predictions. PLOS G P H. 2023;3(2):e0000955.

38. Di Lauro F, Kiss IZ, Rus D, Della Santina C. COVID-19 and flattening the curve: A feedback control perspective. IEEE Control Systems Letters. 2020;5(4):1435–1440.

39. Sharomi O, Malik T. Optimal control in epidemiology. Annals of Operations Research. 2017;251(1-2):55–71.

40. Watkins NJ, Nowzari C, Pappas GJ. Robust economic model predictive control of continuous-time epidemic processes. IEEE Transactions on Automatic Control. 2019;65(3):1116–1131.

41. Morris DH, Rossine FW, Plotkin JB, Levin SA. Optimal, near-optimal, and robust epidemic control. Communications Physics. 2021;4(1):1–8.

42. Hewing L, Wabersich KP, Menner M, Zeilinger MN. Learning-based model predictive control: Toward safe learning in control. Annual Reviews of Control, Robot, and Auto Syst. 2020;3:269–296.

43. Allgöwer F, Zheng A. Nonlinear Model Predictive Control. vol. 26. Birkhäuser; 2012.

44. Tsay C, Lejarza F, Stadtherr MA, Baldea M. Modeling, state estimation, and optimal control for the US COVID-19 outbreak. Scientific Reports. 2020;10(1):1–12.

45. Köhler J, Schwenkel L, Koch A, Berberich J, Pauli P, Allgöwer F. Robust and optimal predictive control of the COVID-19 outbreak. Annual Reviews in Control. 2020;.

46. Morato MM, Bastos SB, Cajueiro DO, Normey-Rico JE. An optimal predictive control strategy for COVID-19 (SARS-CoV-2) social distancing policies in Brazil. Annual Reviews in Control. 2020;50:417–431.

47. Péni T, Csutak B, Szederkényi G, Röst G. Nonlinear Model Predictive Control with logic constraints for COVID-19 management. Nonlinear Dynamics. 2020;102(4):1965–1986.

48. Carli R, Cavone G, Epicoco N, Scarabaggio P, Dotoli M. Model predictive control to mitigate the COVID-19 outbreak in a multi-region scenario. Annual Reviews in Control. 2020;50:373–393.

49. Grundel S, Heyder S, Hotz T, Ritschel TKS, Sauerteig P, Worthmann K. How much testing and social distancing is required to control COVID-19? Some insight based on an age-differentiated compartmental model. arXiv preprint arXiv:201101282. 2020;.

50. Sereno JE, D'Jorge A, Ferramosca A, Hernandez-Vargas EA, Gonzalez AH. Model predictive control for optimal social distancing in a type SIR-switched model. Ifac-papersonline. 2021;54(15):251–256.

51. Acemoglu D, Fallah A, Giometto A, Huttenlocher D, Ozdaglar A, Parise F, et al. Optimal adaptive testing for epidemic control: Combining molecular and serology tests. Automatica. 2024;160:111391.

52. Casella F. Can the COVID-19 epidemic be controlled on the basis of daily test reports? IEEE Control Systems Letters. 2020;5(3):1079–1084.

53. Nowzari C, Preciado VM, Pappas GJ. Analysis and control of epidemics: A survey of spreading processes on complex networks. IEEE Control Systems Magazine. 2016;36(1):26–46.

54. Stewart G, Heusden K, Dumont GA. How control theory can help us control COVID-19. IEEE Spectrum. 2020;57(6):22–29.

55. Richard Q, Alizon S, Choisy M, Sofonea MT, Djidjou-Demasse R. Age-structured non-pharmaceutical interventions for optimal control of COVID-19 epidemic. PLoS Computational Biology. 2021;17(3):e1008776.

56. Zino L, Cao M. Analysis, prediction, and control of epidemics: A survey from scalar to dynamic network models. IEEE Circuits and Systems Magazine. 2021;21(4):4–23.

57. She B, Sundaram S, Paré PE. A Learning-Based Model Predictive Control Framework for Real-Time SIR Epidemic Mitigation. In: Proceedings of the 2022 American Control Conference (ACC). IEEE; 2022. p. 2565–2570.

58. Perkins TA, España G. Optimal control of the COVID-19 pandemic with non-pharmaceutical interventions. Bulletin of Mathematical Biology. 2020;82(9):1–24.

59. Khadilkar H, Ganu T, Seetharam DP. Optimising lockdown policies for epidemic control using reinforcement learning. Transactions of the Indian National Academy of Engineering. 2020;5(2):129–132.

60. Scarabaggio P, Carli R, Cavone G, Epicoco N, Dotoli M. Nonpharmaceutical stochastic optimal control strategies to mitigate the COVID-19 spread. IEEE Transactions on Automation Science and Engineering. 2021;.

61. Mubarak M, Berneburg J, Nowzari C. Individual Non-Pharmaceutical Intervention Strategies for Stochastic Networked Epidemics. In: Proceedings of the 2022 IEEE 61st Conference on Decision and Control (CDC). IEEE; 2022. p. 5627–5632.

62. Bloem M, Alpcan T, Başar T. Optimal and robust epidemic response for multiple networks. Control Engineering Practice. 2009;17(5):525–533.

63. Di Giamberardino P, Iacoviello D. Optimal control of SIR epidemic model with state dependent switching cost index. Biomedical Signal Processing and Control. 2017;31:377–380.

64. Di Giamberardino P, Iacoviello D. Optimal resource allocation to reduce an epidemic spread and its complication. Information. 2019;10(6):213.

65. Liu J, Paré PE, Nedić A, Tang CY, Beck CL, Başar T. Analysis and control of a continuous-time bi-virus model. IEEE Transactions on Automatic Control. 2019;64(12):4891–4906.

66. Dangerfield CE, Vyska M, Gilligan CA. Resource allocation for epidemic control across multiple sub-populations. Bulletin of Mathematical Biology. 2019;81(6):1731–1759.

67. Preciado VM, Zargham M, Enyioha C, Jadbabaie A, Pappas G. Optimal Resource Allocation for Network Protection: A Geometric Programming Approach. IEEE Transactions on Control of Network Systems. 2014;1(1):99–108.

68. Han S, Preciado VM, Nowzari C, Pappas GJ. Data-driven network resource allocation for controlling spreading processes. IEEE Transactions on Network Science and Engineering. 2015;2(4):127–138.

69. Hota AR, Godbole J, Paré PE. A closed-loop framework for inference, prediction, and control of SIR epidemics on networks. IEEE Transactions on Network Science and Engineering. 2021;8(3):2262–2278.

70. She B, Sundaram S, Paré PE. Optimal mitigation of SIR epidemics under model uncertainty. In: Proceedings of the IEEE Conference on Decision and Control (CDC). IEEE; 2022. p. 4333–4338.

71. She B, Xin L, Paré PE, Hale M. Modeling and Predicting Epidemic Spread: A Gaussian Process Regression Approach. arXiv preprint arXiv:231209384. 2023;.

72. Vegvari C, Abbott S, Ball F, Brooks-Pollock E, Challen R, Collyer BS, et al. Commentary on the use of the reproduction number R during the COVID-19 pandemic. Statistical Methods in Medical Research. 2022;31(9):1675–1685.

73. Parag KV, Thompson RN, Donnelly CA. Are epidemic growth rates more informative than reproduction numbers? Journal of the Royal Statistical Society Series A: Statistics in Society. 2022;185(Supplement_1):S5–S15.

74. Abbott S, Hellewell J, Thompson RN, Sherratt K, Gibbs HP, Bosse NI, et al. Estimating the time-varying reproduction number of SARS-CoV-2 using national and subnational case counts. Wellcome Open Research. 2020;5:112.

75. Bracher J, Wolffram D, Deuschel J, Görgen K, Ketterer JL, Ullrich A, et al. A pre-registered short-term forecasting study of COVID-19 in Germany and Poland during the second wave. Nature Communications. 2021;12(1):5173.

List of Figures

S1 A framework for counterfactual analysis, strategy evaluation, and feedback control of epidemics. . . . 2

S2 Daily surveillance testing at UIUC during Fall 2020 and Spring 2021 implemented by the SHIELD team at UIUC. . . . 4

S3 Daily confirmed positive cases at UIUC during Fall 2020 and Spring 2021. 5

S4 Daily voluntary tests at Purdue University during Fall 2020 and Spring 2021. . . . 6

S5 Daily surveillance tests at Purdue University during Fall 2020 and Spring 2021. . . . 6

S6 Daily surveillance tests and confirmed cases through surveillance testing at Purdue University during Fall 2020 and Spring 2021. . . . 6

S7 Daily confirmed positive cases at Purdue University during Fall 2020 and Spring 2021. . . . 7

S8 Total daily confirmed positive cases at Purdue University during Fall 2020 and Spring 2021. . . . 7

S9 Daily isolated cases at Purdue University during Fall 2020 and Spring 2021. 8

S10 Daily quarantined cases at Purdue University during Fall 2020 and Spring 2021. . . . 9

S11 COVID-19 infection profile and serial interval distribution. . . . 11

S12 The simulated effective reproduction number. . . . 13

S13 Simulated daily infected cases. . . . 13

S14 Simulated daily confirmed cases. . . . 14

S15 Infection profiles. . . . 17

S16 The estimated effective reproduction number of the spreading process over the UIUC campus. . . . 20

S17 The estimated effective reproduction number of the spreading process over the Purdue campus. . . . 21

S18 Estimated effective reproduction number of the spreading process over the UIUC campus (7−, 14−, 28−day average data). . . . 21

S19 Estimated effective reproduction number of the spreading process over the UIUC campus ($\mathcal{R}_{t,\tau=14,21,28}$). 22

S20 Reconstructed spreading process over the UIUC campus. 23

S21 Reconstructed spreading process over the Purdue campus. 24

S22 Confirmed cases over the UIUC campus w/ and w/o testing-for-isolation strategies. 27

S23 The effective reproduction number at the UIUC campus w/ and w/o isolation strategies. 28

S24 Confirmed cases at the Purdue campus w/ and w/o isolation under the surveillance testing. 29

S25 The effective reproduction number at the Purdue campus w/ and w/o isolation under the surveillance testing. 29

S26 Daily confirmed cases of the hypothetical outbreak without isolation at the UIUC campus, assuming different proportions follow the isolation rules from the real-world outbreak. 31

S27 Daily confirmed cases of the hypothetical outbreak without isolation at the UIUC campus, assuming different proportions follow the isolation rules from the real-world outbreak. 31

S28 Confirmed cumulative cases of the hypothetical outbreak without isolation at the UIUC campus, assuming different proportions follow the isolation rules from the real-world outbreak. 32

S29 Confirmed cumulative cases of the hypothetical outbreak without isolation at the UIUC campus, assuming different proportions follow the isolation rules from the real-world outbreak. 32

S30 Daily confirmed cases of the hypothetical outbreak with $\underline{\alpha}_P = 1/7$ and $\overline{\alpha}_P = 0.3/7$ at the Purdue campus, assuming different proportions of symptomatic cases during the real-world outbreak. 34

S31 Daily confirmed cases of the hypothetical outbreak with $\underline{\alpha}_P = 1/7$ and $\overline{\alpha}_P = 0.3/7$ at the Purdue campus, assuming different proportions of symptomatic cases during the real-world outbreak. 34

S32 Cumulative confirmed cases of the hypothetical outbreak with $\underline{\alpha}_P = 1/7$ and $\overline{\alpha}_P = 0.3/7$ at the Purdue campus, assuming different proportions of symptomatic cases during the real-world outbreak. 35

S33 Cumulative confirmed cases of the hypothetical outbreak with $\underline{\alpha}_P = 1/7$ and $\overline{\alpha}_P = 0.3/7$ at the Purdue campus, assuming different proportions of symptomatic cases during the real-world outbreak. 35

S34 Daily confirmed cases of the hypothetical spreading scenarios over the Purdue campus, assuming different implemented isolation rates ($\overline{\alpha}_P$) for asymptomatic cases. 36

S35 Daily confirmed cases of the hypothetical spreading scenarios over the Purdue campus, assuming different implemented isolation rates ($\overline{\alpha}_P$) for asymptomatic cases. 37

S36 Cumulative confirmed cases of the hypothetical spreading scenarios over the Purdue campus, assuming different implemented isolation rates ($\overline{\alpha}_P$) for asymptomatic cases. 37

S37 Cumulative confirmed cases of the hypothetical spreading scenarios over the Purdue campus, assuming different implemented isolation rates ($\overline{\alpha}_P$) for asymptomatic cases. 38

S38 Daily confirmed cases of the hypothetical spreading scenarios over the UIUC campus with different isolation rates. 40

S39 Daily confirmed cases of the hypothetical spreading scenarios over the UIUC campus with different isolation rates. 41

S40 Cumulative confirmed cases of the hypothetical spreading scenarios over the UIUC campus with different isolation rates. 41

S41 Cumulative confirmed cases of the hypothetical spreading scenarios over the UIUC campus with different isolation rates. 42

S42 Daily confirmed cases of the hypothetical outbreaks over the Purdue campus with alternative weekly isolation rates under surveillance testing. 43

S43 Daily confirmed cases of the hypothetical outbreaks over the Purdue campus with alternative weekly isolation rates under surveillance testing. 44

S44 Cumulative confirmed cases of the hypothetical outbreaks over the Purdue campus with alternative weekly isolation rates under surveillance testing. 44

S45 Cumulative confirmed cases of the hypothetical outbreaks over the Purdue campus with alternative weekly isolation rates under surveillance testing. 45

S46 Illustration of the closed-loop feedback control framework. 48

S47 Comparison between the fixed and feedback testing-for-isolation strategies at UIUC. 50

S48 Comparison between the fixed and feedback testing-for-isolation strategies at UIUC. 51

S49 Comparison between the fixed and feedback testing-for-isolation strategies in the hypothetical spreading environment at Purdue. 54

S50 Comparison between the fixed and feedback testing-for-isolation strategies in the hypothetical spreading environment at Purdue. 54
